# Exogenous female sex steroid hormones and new-onset asthma in women: a matched case–control study

**DOI:** 10.1186/s12916-023-03038-8

**Published:** 2023-09-04

**Authors:** Guoqiang Zhang, Rani Basna, Maya B. Mathur, Cecilia Lässer, Roxana Mincheva, Linda Ekerljung, Göran Wennergren, Madeleine Rådinger, Bo Lundbäck, Hannu Kankaanranta, Bright I. Nwaru

**Affiliations:** 1 Krefting Research Centre, Department of Internal Medicine and Clinical Nutrition, Institute of Medicine, Sahlgrenska Academy, University of Gothenburg, Gothenburg, Sweden; 2 Quantitative Sciences Unit, Stanford University, Palo Alto, CA USA; 3 Department of Pediatrics, Sahlgrenska Academy, University of Gothenburg, Gothenburg, Sweden; 4 Department of Respiratory Medicine, Seinäjoki Central Hospital, Seinäjoki, Finland; 5 Faculty of Medicine and Health Technology, University of Tampere, Tampere, Finland; 6 Wallenberg Centre for Molecular and Translational Medicine, University of Gothenburg, Gothenburg, Sweden

**Keywords:** Asthma, Bayesian estimation, Case–control, Hormonal contraceptives, Menopausal hormone therapy, Women

## Abstract

**Background:**

Evidence on the role of exogenous female sex steroid hormones in asthma development in women remains conflicting. We sought to quantify the potential causal role of hormonal contraceptives and menopausal hormone therapy (MHT) in the development of asthma in women.

**Methods:**

We conducted a matched case–control study based on the West Sweden Asthma Study, nested in a representative cohort of 15,003 women aged 16–75 years, with 8-year follow-up (2008–2016). Data were analyzed using Frequentist and Bayesian conditional logistic regression models.

**Results:**

We included 114 cases and 717 controls. In Frequentist analysis, the odds ratio (OR) for new-onset asthma with ever use of hormonal contraceptives was 2.13 (95% confidence interval [CI] 1.03–4.38). Subgroup analyses showed that the OR increased consistently with older baseline age. The OR for new-onset asthma with ever MHT use among menopausal women was 1.17 (95% CI 0.49–2.82). In Bayesian analysis, the ORs for ever use of hormonal contraceptives and MHT were, respectively, 1.11 (95% posterior interval [PI] 0.79–1.55) and 1.18 (95% PI 0.92–1.52). The respective probability of each OR being larger than 1 was 72.3% and 90.6%.

**Conclusions:**

Although use of hormonal contraceptives was associated with an increased risk of asthma, this may be explained by selection of women by baseline asthma status, given the upward trend in the effect estimate with older age. This indicates that use of hormonal contraceptives may in fact decrease asthma risk in women. Use of MHT may increase asthma risk in menopausal women.

**Supplementary Information:**

The online version contains supplementary material available at 10.1186/s12916-023-03038-8.

## Background

Asthma is a common heterogeneous respiratory disease affecting 1–18% of the population in different countries [1]. In 2019, asthma was estimated to affect approximately 262 million people and cause 461 thousand deaths worldwide, constituting a major global health burden [2]. For decades, age- and sex-related differences in asthma have been reported across different continents [3]. During childhood, asthma is more common in boys than in girls, while from around puberty onwards, it becomes more common in women than in men [3]. Compared to asthma that occurs in childhood, asthma that occurs in adulthood, which mainly affects women, is often more severe and has a faster decline in lung function and a poorer prognosis, representing a distinct clinical phenotype of asthma [4–6]. Because the switch in asthma occurs around the onset of puberty, sex steroid hormones (estrogens, progestogens and androgens) have been hypothesized to be implicated in the pathogenesis of adult-onset asthma [3, 7].

In parallel with the hypotheses, mechanistic studies suggest that female sex steroid hormones play an important role in asthma pathogenesis [3, 8]. Meanwhile, a number of epidemiologic studies have investigated the relation of hormonal contraceptives and menopausal hormone therapy (MHT) to the risk of new-onset asthma in adult women [3, 9]. Three cohort studies on hormonal contraceptives and new-onset asthma reported conflicting results [10–12]. An umbrella review [13] and a Danish register-based nested case–control study [14] reported an increased risk of new-onset asthma with MHT use in menopausal women, which, however, was contradicted by a UK national cohort study [15]. Overall, the evidence remains uncertain, mostly due to the concerns over potential systematic biases in most existing studies, such as selection bias, inadequate consideration of the full range of potential confounders, and, more essentially, lack of explicit causal reasoning [3, 9, 16, 17]. This makes it challenging, if not impossible, to establish whether the role of exogenous female sex steroid hormones in the development of asthma is causal [17, 18].

In the current study, we sought to determine the association between use of hormonal contraceptives and MHT and new-onset asthma in adult women, accounting for various sources of potential biases, in an attempt to assess whether this association is causal. In doing this, we used causal diagrams to represent and classify potential systematic biases and applied both Frequentist and Bayesian statistical models. The Bayesian model can incorporate our *a priori* background knowledge on the topic into the analysis and, compared to the conventional Frequentist analysis, is generally more robust to the size and the quality of the sample data [19, 20]. Our *a priori* hypotheses [3, 21] were that use of hormonal contraceptives, which suppresses the activities of endogenous female sex hormones, would reduce the risk of new-onset asthma, whereas use of MHT, which enhances the levels of endogenous female sex hormones, would increase the risk in menopausal women. To our knowledge, this is the first study that applies the Bayesian approach to investigate the causal role of exogenous female sex steroid hormones in the development of asthma in adult women.

## Methods

This study was reported according to the Strengthening the Reporting of Observational Studies in Epidemiology (STROBE) statement (Additional file 1) [22]. We formulated the research questions according to the PECOS components (Population, Exposure, Comparator, Outcome, and Study design) [23].

### Study population

**Fig. 1:**
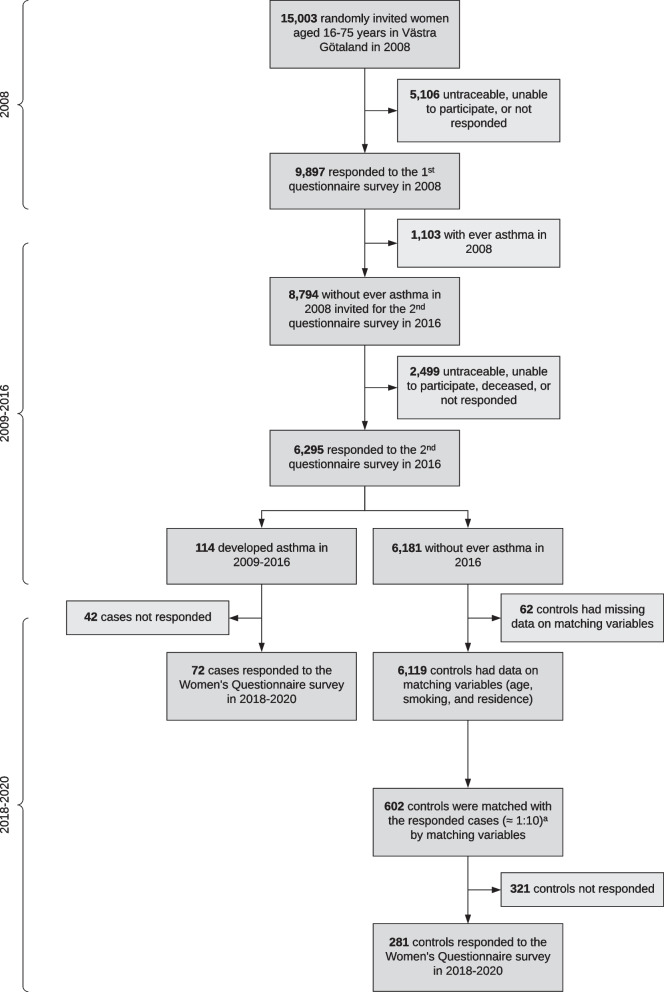
Flow diagram for selection of participants in the study. Superscript lowercase letter a (^a^) indicates the following: in some matched strata, cases had less than 10 controls

The West Sweden Asthma Study (WSAS) is a population-based, longitudinal study established in West Sweden in 2008, which has been described in detail elsewhere [24]. At study baseline in 2008, the first questionnaire survey was sent to 30,000 randomly selected adults aged 16–75 years in western Sweden, of which 15,003 were women (out of which 9897 (66%) responded) (Fig. 1). Of the respondents, we excluded 1103 women who reported ever had asthma or ever diagnosed with asthma by a doctor. In 2016, the second questionnaire survey was sent to the remaining 8794 women, out of which 6295 (72%) responded. Of the respondents, 114 developed asthma during the 8-year follow-up. For use of hormonal contraceptives, the study population was based on all responding women, including 114 who had new-onset asthma and 6181 who had never had asthma by 2016. For use of MHT, the study population was restricted to 3641 women of menopausal age ($\ge$ 45 years) at baseline, including 54 who had new-onset asthma and 3587 who had never had asthma by 2016.

### Study design

In 2018–2020, the GA^2^LEN Women’s Questionnaire survey was sent to the 114 cases that had developed asthma during the 8-year follow-up to obtain information on hormonal exposures, out of which 72 (63%) responded. The 72 responding cases were individually matched to 602 controls, out of which 281 (47%) responded to the survey. The matching variables were exact age in years in 2008, place of residence (in or outside Gothenburg), and smoking status (never smoker, former smoker, or current smoker). The choice of a relatively high number of controls per case was to account for potential non-response among the controls. More details on individual matching are provided in Additional file 2 [3, 6, 9, 13, 17, 19, 24–91].

### Hormonal exposures

The exposures of interest included ever use of hormonal contraceptives or MHT, which were reported by participants in the Women’s Questionnaire survey in 2018–2020. Use of hormonal contraceptives was measured by “Have you ever taken a treatment which contains hormones to stop you from getting pregnant (e.g., tablets, depot injection, hormonal coil)?” Use of MHT was measured by “Have you ever taken a treatment which contains hormones to reduce the symptoms or effects of menopause (e.g., ‘HRT’ tablets, depot injection, patches, gels, but not vaginal creams or pessaries)?” If participants answered “yes” to either question, they were further asked “How old were you when you first took the treatment?” The respective comparator group was never use of hormonal contraceptives or MHT.

### New-onset asthma

Women who reported never having had asthma or doctor-diagnosed asthma during the first questionnaire survey in 2008 but later reported that they had asthma or doctor-diagnosed asthma during the second questionnaire survey in 2016 were considered as having developed new-onset asthma. Women who reported asthma were further asked “How old were you when you got asthma?”

### Directed acyclic graphs

We used causal directed acyclic graphs (DAGs) to represent potential systematic biases in our study [30, 92]. Details on DAGs are provided in Additional file 2. For confounding bias, we identified potential common causes of each hormonal exposure and new-onset asthma based on previous literature (Additional file 2: Table S1); then, we applied the backdoor criterion to determine a sufficient set of adjustment variables required to minimize confounding (Additional file 2: Figs. S1 and S2) [26]. For use of hormonal contraceptives, the adjusted variables included age, place of residence, level of education, age at menarche, gynecological conditions (including endometriosis, polycystic ovarian syndrome, gynecological acne, and hysterectomy with or without oophorectomy), and tobacco smoking. For use of MHT, the adjusted variables included age, place of residence, level of education, body mass index, tobacco smoking, environmental tobacco smoke, age at menopause, physical exercise, and gynecological conditions. The definitions of the adjusted variables are summarized in Additional file 3 [93]. The DAGs for selection bias and measurement bias are respectively presented in Additional file 2: Figs. S3 and S4. Particularly, for many women, hormonal exposures happened before the study had started; if hormonal exposures had a causal effect on new-onset asthma, restricting the study population to those who had never had asthma at baseline would likely result in differential proportion of susceptible individuals after baseline, thereby introducing selection bias [27].

### Statistical analyses

We developed *a priori* an analysis protocol including justifications for the statistical methods applied in this study (Additional file 2). In brief, for each hormonal exposure we applied Frequentist conditional logistic regression model to adjust for the matching sets and measured confounding variables [38, 49, 51]. We used multiple imputation (MI)—full-conditional specification (FCS) [44, 46]—to impute the missing data and fitted Frequentist conditional logistic regression model to the multiply imputed datasets ($m=$ 100). Then, we conducted complete-case analysis as a sensitivity analysis, that is, restricting the analysis to individuals with complete data on all variables included in the model. The results were summarized as odds ratio (OR) with 95% confidence interval (CI). We conducted subgroup analyses by baseline age (above or below an age cut-off) to evaluate potential selection bias [94] and calculated *E*-value [69] to assess the robustness of the estimated causal effects to potential residual confounding.

We applied Bayesian conditional logistic regression model in the multiply imputed datasets (Additional file 2) [64, 66]. The Bayesian model approximated the posterior probability distributions over all possible values of the parameters of interest, conditional on the prior probability distributions, statistical model and observed data [58]. For parameters of hormonal exposure variables, we derived *a priori* original prior distributions (use of hormonal contraceptives: $\mathrm{log}OR\sim N(-0.26,{0.20}^{2})$; use of MHT: $\mathrm{log}OR\sim N(0.17,{0.13}^{2})$), based on our previous review work [3, 9, 13] as well as newly published studies [11, 14, 15]. In addition, we set three alternative prior distributions to represent our uncertainty about the original prior distribution and used a flat prior distribution (which assigns equal prior plausibility to all possible values of a parameter) to understand the influence of our prior knowledge compared to that of the observed data on the model results [63]. The process of deriving the prior distributions is available in Additional file 4 [3, 10–15, 69]. Finally, we used the Markov chain Monte Carlo (MCMC) method to approximate the posterior distributions [64, 66] and calculated the median and the 95% central posterior interval (PI) on OR scale [68]. The 95% central PI means that, given the prior distributions, statistical model, and observed data, the true causal effect has a 95% probability of falling within this range [58]. We estimated the probability that each hormonal exposure would increase the risk of new-onset asthma in women [58].

As asthma is relatively rare in our study population (< 15% by the end of follow-up), the estimated ORs can be approximately interpreted as risk ratios [69]. All statistical analyses were performed using the R software (version 4.0.4) [95]. The R scripts and packages [45, 64, 69, 96–106] are available in Additional files 5 and 6.

## Results

### Characteristics of the study population

In total, 72 cases and 281 controls responded to the Women’s Questionnaire survey, among which 62 cases (86.1%) and 204 controls (72.6%) had ever used hormonal contraceptives. The median age when starting hormonal contraceptives was 18 years (range: 13–49 years). Thirty-five cases and 150 controls were aged 45 years or older at baseline, among which eight cases (22.9%) and 25 controls (16.7%) had ever used MHT. The median age of starting MHT was 52 years (range: 38–64 years). Forty-two of the 114 cases did not respond to the Women’s Questionnaire survey and were matched with additional 115 controls, resulting in a total of 114 cases and 717 controls. Tables 1 and 2 summarize the background characteristics for all cases and matched controls and for hormone users and never users, respectively. The median age of participants at baseline was 44 years (range: 19–74 years). Cases were more likely to have a BMI of $\ge$ 30 kg/m^2^ and a higher level of education (although not statistically significant). Hormonal contraceptive users were more likely to be younger and have a lower BMI and a higher level of education, compared to never user. MHT users were more likely to have a lower BMI, be a former smoker, and live in Gothenburg city, compared to never user. The PECOS components, the results from complete-case analysis, and the MI process are detailed in Additional file 3. Below, we describe the results from both Frequentist and Bayesian analyses based on the multiply imputed datasets.

**Table 1:** Background characteristics of new-onset asthma cases and matched controls in WSAS in 2009–2016

**Characteristics**^a^	**Cases (*N* = 114)** ***n* (%)**	**Controls (*N* = 717)** ***n* (%)**	***P* value**^b^
Age (years), mean (SD)	44.0 (14.7)	44.7 (14.4)	0.632
BMI (kg/m^2^)			0.075
< 25	78 (68.4)	469 (65.4)	
25–29.9	18 (15.8)	174 (24.3)	
≥ 30	15 (13.2)	64 (8.9)	
Missing	3 (2.6)	10 (1.4)	
Smoking status			0.649
Never smoker	63 (55.3)	402 (56.1)	
Former smoker	41 (36.0)	268 (37.4)	
Current smoker	10 (8.8)	47 (6.6)	
Place of residence			0.919
Gothenburg	65 (57.0)	414 (57.7)	
Outside Gothenburg	49 (43.0)	303 (42.3)	
Level of education			0.204
Less than high school	12 (10.5)	122 (17.0)	
High school	31 (27.2)	201 (28.0)	
Tertiary level	68 (59.6)	389 (54.3)	
Missing	3 (2.6)	5 (0.7)	
Use of hormonal contraceptives			0.077
No	10 (8.8)	65 (9.1)	
Yes	62 (54.4)	204 (28.5)	
Missing	42 (36.8)	448 (62.5)	
Use of MHT^c^			0.464
No	27 (50.0)	123 (34.6)	
Yes	8 (14.8)	25 (7.0)	
Missing	19 (35.2)	207 (58.3)	

*Abbreviations*: *BMI* body mass index, *MHT* menopausal hormone therapy, *SD* standard deviation

^a^Age, body mass index and place of residence were based on the 2008 questionnaire survey; smoking status and level of education were based on the 2016 questionnaire survey; the hormonal exposures were based on the Women’s Questionnaire survey in 2018–2020

^b^Student’s *t*-test was used for continuous variables, and Fisher’s exact test for categorical variables

^c^Among 409 women aged $\ge$ 45 years at baseline in 2008

**Table 2:** Background characteristics of hormone users and never users in the study sample

**Characteristics**^a^	**Hormonal contraceptives**		***P* value**^b^	**MHT**^c^		***P* value**^b^
	**Ever use (*N* = 266)** ***n* (%)**	**Never use (*N* = 75)** ***n* (%)**		**Ever use (*N* = 33)** ***n* (%)**	**Never use (*N* = 150)** ***n* (%)**	
Age (years), mean (SD)	43.3 (13.2)	51.3 (13.1)	$<$ 0.001	56.4 (7.3)	55.8 (8.1)	0.664
BMI (kg/m^2^)			0.092			0.149
< 25	185 (69.5)	43 (57.3)		25 (75.8)	83 (55.3)	
25–29.9	52 (19.5)	23 (30.7)		6 (18.2)	43 (28.7)	
≥ 30	25 (9.4)	8 (10.7)		2 (6.1)	20 (13.3)	
Missing	4 (1.5)	1 (1.3)		–	4 (2.7)	
Smoking status			0.415			0.042
Never smoker	149 (56.0)	39 (52.0)		7 (21.2)	57 (38.0)	
Former smoker	105 (39.5)	30 (40.0)		26 (78.8)	84 (56.0)	
Current smoker	12 (4.5)	6 (8.0)		0	9 (6.0)	
Place of residence			0.421			0.055
Gothenburg	167 (62.8)	43 (57.3)		23 (69.7)	76 (50.7)	
Outside Gothenburg	99 (37.2)	32 (42.7)		10 (30.3)	74 (49.3)	
Level of education			0.010			0.696
Less than high school	22 (8.3)	15 (20.0)		5 (15.2)	30 (20.0)	
High school	81 (30.5)	24 (32.0)		13 (39.4)	48 (32.0)	
Tertiary level	163 (61.3)	35 (46.7)		15 (45.5)	72 (48.0)	
Missing	–	1 (1.3)		–	–	

*Abbreviations*: *BMI* body mass index, *MHT* menopausal hormone therapy, *SD* standard deviation

^a^Age, body mass index and place of residence were based on the 2008 questionnaire survey; smoking status and level of education were based on the 2016 questionnaire survey

^b^Student’s *t*-test was used for continuous variables, and Fisher’s exact test for categorical variables

^c^Among 183 women aged $\ge$ 45 years at baseline in 2008

### Use of hormonal contraceptives and new-onset asthma

#### Frequentist analysis

**Fig. 2:**
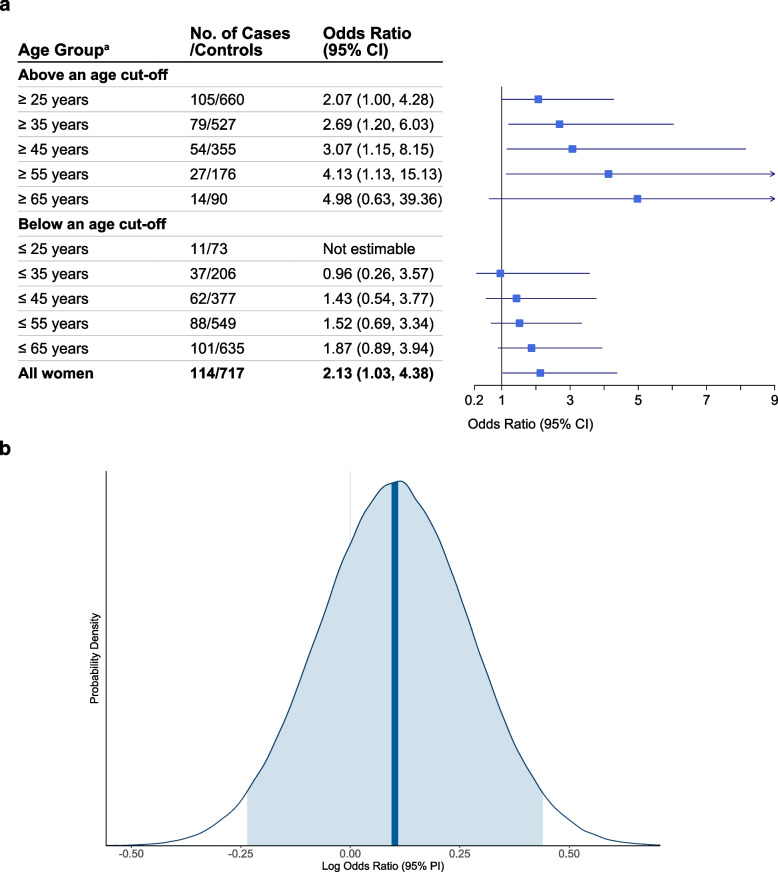
Ever use of hormonal contraceptives (compared to never use) and new-onset asthma in women. **a** The results were from Frequentist conditional logistic regression. **b** Posterior probability distribution estimated from Bayesian conditional logistic regression, based on the original prior probability distribution $\mathrm{log}OR\sim N(-0.26,{0.20}^{2})$ for use of hormonal contraceptives; the vertical bold line indicates the median of the posterior distribution, and the shaded area under the curve indicates the 95% central posterior interval. All analyses were conducted based on the multiply imputed datasets ($m=$ 100), adjusted for age, place of residence, level of education, age at menarche, gynecological conditions and tobacco smoking. Abbreviations: CI, confidence interval; PI, posterior interval. Superscript lowercase letter a (^a^) indicates the following: age at baseline in 2008

The effect estimate for asthma development with ever use of hormonal contraceptives compared to never use was OR 2.13 (95% CI 1.03–4.38) (Fig. 2a). Subgroup analyses that restricted to participants above a series of age cut-offs showed that the point effect estimate increased consistently with increasing baseline age: OR 2.07 (95% CI 1.00–4.28) among women aged $\ge$ 25 years, 2.69 (95% CI 1.20–6.03) among $\ge$ 35 years, 3.07 (95% CI 1.15–8.15) among $\ge$ 45 years, 4.13 (95% CI 1.13–15.13) among $\ge$ 55 years, and 4.98 (95% CI 0.63–39.36) among $\ge$ 65 years. The magnitude of point estimate among women below an age cut-off remained consistently lower than that among those above that cut-off. The *E*-value for the point estimate among all women was 3.68, which meant that the observed OR of 2.13 could be explained away by unmeasured confounder(s) that were associated with both the exposure and the outcome by a risk ratio of 3.68-fold each, above and beyond the measured confounders, but weaker confounding could not do so.

#### Bayesian analysis

The posterior distribution for asthma development with ever use of hormonal contraceptives based on the original prior is presented in Fig. 2b. The median of the posterior distribution on OR scale was 1.11 (95% PI 0.79–1.55), and the probability of OR being larger than 1 was 72.3%. Prior sensitivity analysis using three alternative priors showed an upward shift in the median of the posterior distribution: OR 1.22 (95% PI 0.82–1.80), 1.35 (95% PI 0.86–2.12), and 1.51 (95% PI 0.91–2.51), respectively (Additional file 4: Table S1). The respective probabilities of OR being larger than 1 were 84.0%, 90.7%, and 94.4%. The posterior distribution based on flat priors on OR scale had a median of 2.17 (95% PI 1.08–4.64), and the probability of OR being larger than 1 reached up to 98.5%. Additional file 4: Fig. S5 illustrates and compares the posterior distributions based on the different prior distributions.

### Use of MHT and new-onset asthma

#### Frequentist analysis

**Fig. 3:**
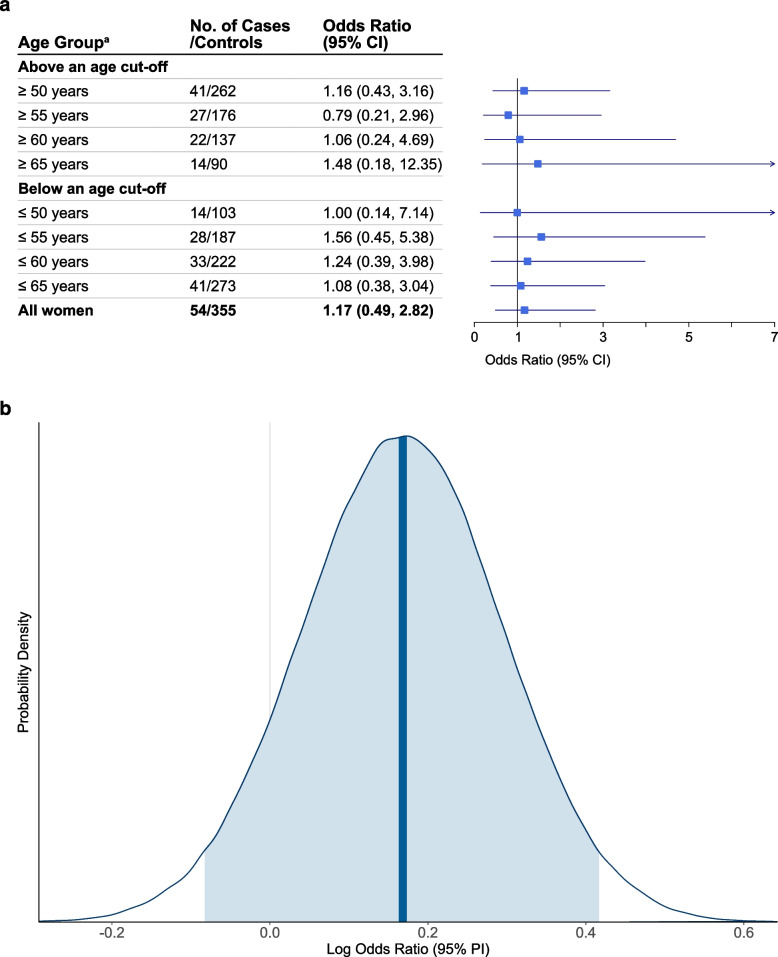
Ever use of menopausal hormone therapy (compared to never use) and new-onset asthma in menopausal women. **a** The results were from Frequentist conditional logistic regression. **b** Posterior probability distribution estimated from Bayesian conditional logistic regression, based on the original prior probability distribution $\mathrm{log}OR\sim N(0.17,{0.13}^{2})$ for use of menopausal hormone therapy; the vertical bold line indicates the median of the posterior distribution, and the shaded area under the curve indicates the 95% central posterior interval. All analyses were conducted based on the multiply imputed datasets ($m=$ 100), adjusted for age, place of residence, level of education, body mass index, tobacco smoking, environmental tobacco smoke, age at menopause, physical exercise and gynecological conditions. Abbreviations: CI, confidence interval; PI, posterior interval. Superscript lowercase letter a (^a^) indicates the following: age at baseline in 2008

Among menopausal women, the effect estimate for asthma development with ever use of MHT compared to never use was OR 1.17 (95% CI 0.49–2.82) (Fig. 3a). Subgroup analyses by baseline age did not show any pattern in the point estimates. The *E*-value for the point estimate among all menopausal women was 1.62, which meant that the observed OR of 1.17 could be explained away by unmeasured confounder(s) that were associated with both the exposure and the outcome by a risk ratio of 1.62-fold each, above and beyond the measured confounders, but weaker confounding could not do so.

#### Bayesian analysis

The posterior distribution for asthma development with ever use of MHT based on the original prior is presented in Fig. 3b. The median of the posterior distribution on OR scale was 1.18 (95% PI 0.92–1.52), and the probability of OR being larger than 1 was 90.6%. Prior sensitivity analysis using three alternative priors showed an increase in the width of the 95% PI: OR 1.18 (95% PI 0.87–1.60), 1.18 (95% PI 0.82–1.70), and 1.18 (95% PI 0.76–1.82), respectively (Additional file 4: Table S2). The respective probabilities of OR being larger than 1 were 86.2%, 81.6%, and 77.2%. The posterior distribution based on flat priors on OR scale had a median of 1.17 (95% PI 0.45–2.81), and the probability of OR being larger than 1 dropped down to 62.8%. Additional file 4: Fig. S10 illustrates and compares the posterior distributions based on the different prior distributions.

## Discussion

### Summary of key findings

In Frequentist analysis, ever use of hormonal contraceptives compared to never use was associated with an increased risk of new-onset asthma in women. Subgroup analyses showed that the association consistently became stronger among women of older age at baseline. Among menopausal women, ever use of MHT compared to never use was statistically non-significantly associated with an increased risk of new-onset asthma. In Bayesian analysis, although the 95% PIs for both use of hormonal contraceptives and MHT included the null value, there was a 72% and 91% probability that the OR was larger than one for hormonal contraceptives and MHT, respectively.

### Strengths and limitations

There are several strengths in our study. First, being a case–control study nested within an ongoing prospective cohort, we could ascertain the temporality between the exposure and the outcome and thereby apply models to allow estimation of potential causal effects of exogenous female sex hormones on asthma risk. Second, we built causal DAGs based on published literature and our *a priori* subject-matter knowledge to identify potential confounding variables for each hormonal exposure [26, 30], which provides an explicit framework to minimize confounding. Third, in order to reduce the potential bias introduced by item non-response, we implemented MI to impute the missing data [39]. Fourth, we applied Bayesian statistical model which incorporated our prior background knowledge into the analysis; in this way, the current analysis provides a solid basis for future analyses. Finally, we adopted open and reproducible research practices [107]—we developed *a priori* statistical analysis protocol, documented in detail the research process, and made R scripts publicly available.

Certain limitations need to be taken into account in the interpretation of our findings. First, in our study, women with ever asthma at baseline were excluded, and for many women, hormonal exposures occurred before the study had started. This could have introduced selection bias, especially among older women, *only* if hormonal exposures had a causal effect on new-onset asthma (Additional file 2: Fig. S3) [27]. For example, *if* hormonal contraceptives increased the risk of new-onset asthma, the more susceptible individuals would have developed asthma before baseline in the exposed group than in the unexposed group; consequently, restricting to individuals who had not developed asthma by baseline would likely result in less susceptible individuals after baseline in the exposed group than in the unexposed group, thereby attenuating the magnitude of the effect estimate for the true harmful effect or even biasing the effect estimate towards the opposite direction [27]. Contrary to the hypothetical example, in our study, we found that when the age at baseline increased, the magnitude of point estimate for use of hormonal contraceptives consistently increased. We suspect that selection bias due to selection of women based on baseline asthma status may likely be the main explanation for the increase of the point estimate with increasing baseline age. This suggests that use of hormonal contraceptives may in fact have a protective effect on new-onset asthma, as opposed to a harmful effect indicated by our results. Notably, this type of selection bias may arise in any study that attempts to estimate the causal effect of an (lifetime) exposure that occurs before the study has started [27, 94, 108, 109]. Second, because some identified confounding variables were not available in our dataset, we had to rely on proxy variables (e.g., for socioeconomic status), or could not adjust for them at all (e.g., diet, alcohol) for use of MHT and new-onset asthma. Third, the information on hormonal exposures and asthma status was obtained retrospectively by questionnaire survey. Thus, an individual’s ability to recall their medical history may affect the measurement of both hormonal exposures and asthma (Additional file 2: Fig. S4) [92]. In addition, because hormonal exposures were ascertained by recall after asthma had occurred, asthma status might affect the recall of hormonal exposures. However, we expect that the influence was likely to be minimal (if present), because the causal relationship between asthma and hormonal exposures had not been well established. Fourth, more than half of the cases did not report age at asthma onset, which could potentially affect estimation of the temporal relationship between hormonal exposures and new-onset asthma. However, for the cases who reported age at asthma onset, asthma occurred after use of hormonal contraceptives or MHT. Fifth, although WSAS is a population-based study of a representative sample in western Sweden, given the baseline participation rate of 66% and the follow-up rate of 72%, potential selection bias (i.e., bias due to unit non-response) may have arisen, which could potentially affect the estimation of causal effects of exogenous female sex hormones on asthma risk as well as the generalization of our results to the source population. Unfortunately, we were unable to account for this type of bias in our study. Sixth, we could not investigate the potential varying causal effects of use of hormonal contraceptives and MHT by subtypes, doses, durations of use, and routes of administration, because the information on these factors could not be reliably determined from the questionnaire survey. Seventh, the study population for MHT use included women aged $\ge$ 45 years at baseline, which was used as a proxy measure to identify menopausal women. Since it was not based on the actual menopausal status, this may affect the generalization of our results to the menopausal group of women. Finally, due to data unavailability, we were unable to address the potential modifying effect of BMI for the effects of exogenous female sex hormones on asthma risk.

### Comparison to previous studies

To our knowledge [3], three cohort studies have investigated the causal effects of use of hormonal contraceptives on the risk of new-onset asthma, which excluded women with ever asthma at baseline to form the study population [10–12]. Interestingly, a similar upward trend existed in the effect estimate with increasing baseline age across or within studies (Additional file 7: Fig. S1 [10–12]). Likewise, we suspect that selection bias may likely explain the increase and the reversal of the relative risk with increasing baseline age.

Recently, an umbrella review including five cohort studies [13] and a nested case–control study based on the Danish registers [14] reported that use of MHT was associated with an increased risk of new-onset asthma in menopausal women. In contrast, a UK national cohort study [15], the largest longitudinal study on the topic to date, found that use of MHT was associated with a decreased risk of new-onset asthma and that longer duration of use was associated with a lower asthma risk than shorter duration. It is unclear whether different subtypes of MHT, population characteristics, or asthma phenotypes could explain the heterogeneity in these results.

### Implications

First, despite intensive investigations, the epidemiologic evidence on female sex hormones and asthma risk remains equivocal [3, 9, 16]. Future longitudinal studies that account for systematic biases will help advance the evidence. This will benefit from making explicit the causal goals of research [17], and using causal diagrams (e.g., DAGs) to represent and classify systematic biases [25], which formulates a clearer framework for evaluating the proposed causal structures and analytical approaches, thus facilitating explicit causal reasoning of the results. Second, in studies of hormonal exposures that occur early in life (e.g., use of hormonal contraceptives), special attention needs to be paid to potential selection bias [27]. Indeed, for the upward trend in the effect estimate with increasing baseline age observed in our study and across previous studies, other explanations may include effect modification by age (i.e., use of hormonal contraceptives beneficial at younger ages but harmful at older ages) or residual confounding (i.e., the strength of residual confounding varied systematically across different age groups). Future studies that account for different systematic biases will hopefully provide further insights. Third, longitudinal studies that investigate the potential varying causal effects of use of hormonal contraceptives and MHT by subtypes, doses, durations of use, and routes of administration on various phenotypes of asthma are warranted. This can help generate evidence for more individualized asthma prevention strategies for women. Fourth, future studies are needed to investigate the potential modifying effect of BMI for the effects of exogenous female sex hormones on asthma risk. Notably, because BMI can arguably be a mediator along the causal pathway between hormonal contraceptives and new-onset asthma [80–82], future studies that carefully collect data on relevant key variables are warranted (e.g., physical activity, alcohol, stress, diet) (Additional file 7: Fig. S2).

### Bayesian inference

The Bayesian framework naturally incorporates the investigators’ prior background knowledge about the parameters of interest before observing the data (i.e., prior beliefs) and updates these beliefs about the parameters after observing the data (i.e., posterior beliefs) [19]. This can help improve precision in effect estimate when the sample data is relatively small. For example, the incorporation of our prior knowledge from previous studies of use of MHT and asthma produced an effect estimate with substantially more certainty than that from only relying on the sample data. On the other hand, when the quality of the sample data is not optimal, the Bayesian method can help to some degree mitigate the influence of potential systematic biases on the results; in contrast, the Frequentist method relies only on the sample data and thus is highly dependent on data quality [19]. For example, the inclusion of our prior knowledge on use of hormonal contraceptives and asthma yielded a conservative result as opposed to that from Frequentist analysis; the wide spread of the posterior distribution means that more evidence is needed, which favorably kept us from making an overconfident claim that use of hormonal contraceptives would increase asthma risk in women. Furthermore, Bayesian analysis allows us to make intuitive probabilistic statements about the parameters [19, 110]. We can say that, for example, there was a 91% probability that ever use of MHT would increase asthma risk in menopausal women, conditional on the priors, statistical model and observed data. However, Frequentist estimates are often misinterpreted as Bayesian estimates [19]. A criticism of Bayesian analysis is that the priors are subjective [19]. However, it is noteworthy that this subjectivity creeps into both Frequentist and Bayesian analyses, where in Frequentist analysis, flat priors are implicitly assumed and may not always realistically capture *a priori* knowledge [19, 20]. The Bayesian framework makes explicit this aspect of subjectivity and uses different distributions to quantify this subjectivity [20].

## Conclusions

Although use of hormonal contraceptives was associated with an increased risk of asthma, this may be explained by selection of women by baseline asthma status, given the upward trend in the effect estimate with older age. This indicates that use of hormonal contraceptives may in fact decrease asthma risk in women. Use of MHT may increase asthma risk in menopausal women.

### Supplementary Information

**Additional file 1.** STROBE checklist for reports of case–control studies.

**Additional file 2:** **Table S1.** Potential common causes of use of hormonal contraceptives or menopausal hormone therapy and new-onset asthma in women. **Figure S1.** A causal directed acyclic graph for potential common causes of use of hormonal contraceptives and new-onset asthma in women. **Figure S2.** A causal directed acyclic graph for potential common causes of use of menopausal hormone therapy and new-onset asthma in menopausal women. **Figure S3.** A causal directed acyclic graph for potential selection bias of hormonal exposures and new-onset asthma in women. **Figure S4.** A causal directed acyclic graph for potential measurement bias of hormonal exposures and new-onset asthma in women.

**Additional file 3:** **Section 1.** Supplementary methods and results for the Frequentist analysis of hormonal contraceptives and new-onset asthma in women. **Table S1.** Background characteristics of the responded new-onset asthma cases and matched controls in WSAS in 2009–2016. **Figure S1.** Age distribution at baseline in 2008 among the responded women (*N* = 353). **Figure S2.** Age at first use of hormonal contraceptives in 261 women. **Figure S3.** Age at asthma diagnosis among 32 cases. **Figure S4.** Ever use of hormonal contraceptives (compared to never use) and new-onset asthma in women. **Figure S5.** Comparison between women who had data on the incomplete variables and those who did not with respect to auxiliary variables. **Figure S6.** Healthy convergence for the incomplete variables. **Figure S7.** The distribution of use of hormonal contraceptives in the observed data and in the m = 100 imputed datasets. **Figure S8.** Kernel density estimates for the marginal distributions of the observed data for age at menarche and the m = 100 densities calculated from the imputed data. **Figure S9.** The distribution of gynecological condition in the observed data and in the m = 100 imputed datasets. **Figure S10.** The distribution of level of education in the observed data and in the m = 100 imputed datasets. **Figure S11.** Odds ratios for ever use of hormonal contraceptives (compared to never use) and new-onset asthma among all women (*N* = 831) across the m = 100 imputed datasets. **Section 2.** Supplementary methods and results for the Frequentist analysis of menopausal hormone therapy and new-onset asthma in menopausal women. **Figure S12.** Age at first use of menopausal hormone therapy in 32 women. **Figure S13.** Age at asthma diagnosis among 13 cases. **Figure S14.** Ever use of menopausal hormone therapy (compared to never use) and new-onset asthma in menopausal women. **Figure S15.** Comparison between women who had data on the incomplete variables and those who did not with respect to auxiliary variables. **Figure S16.** Healthy convergence for the incomplete variables. **Figure S17.** The distribution of use of menopausal hormone therapy in the observed data and in the m = 100 imputed datasets. **Figure S18.** Kernel density estimates for the marginal distributions of the observed data for age at menopause and the m = 100 densities calculated from the imputed data. **Figure S19.** The distribution of physical exercise in the observed data and in the m = 100 imputed datasets. **Figure S20.** Kernel density estimates for the marginal distributions of the observed data for body mass index and the m = 100 densities calculated from the imputed data. **Figure S21.** The distribution of level of education in the observed data and in the m = 100 imputed datasets. **Figure S22.** The distribution of environmental smoke in the observed data and in the m = 100 imputed datasets. **Figure S23.** The distribution of gynecological condition in the observed data and in the m = 100 imputed datasets. **Figure S24.** Odds ratios for ever use of menopausal hormone therapy (compared to never use) and new-onset asthma among all menopausal women (*N* = 409) across the m = 100 imputed datasets.

**Additional file 4:** **Section 1.** Supplementary methods and results for the Bayesian analysis of hormonal contraceptives and new-onset asthma in women. **Table S1.** Posterior estimates for the original and alternative priors for use of hormonal contraceptives and new-onset asthma in women. **Figure S1.** The original and alternative prior distributions. **Figure S2.** Posterior distributions estimated from each multiply imputed dataset (m = 100). **Figure S3.** Posterior distributions estimated from each Markov chain in the first imputed dataset. **Figure S4.** Trace plot from the first imputed dataset. **Figure S5.** Posterior distributions for the original and alternative priors. **Section 2**: Supplementary methods and results for the Bayesian analysis of menopausal hormone therapy and new-onset asthma in menopausal women. **Table S2.** Posterior estimates for the original and alternative priors for use of menopausal hormone therapy and new-onset asthma in menopausal women. **Figure S6.** The original and alternative prior distributions. **Figure S7.** Posterior distributions estimated from each multiply imputed dataset (m = 100). **Figure S8.** Posterior distributions estimated from each Markov chain in the first imputed dataset. **Figure S9.** Trace plot from the first imputed dataset. **Figure S10.** Posterior distributions for the original and alternative priors.

**Additional file 5.** R scripts for use of hormonal contraceptives and new-onset asthma in women.

**Additional file 6.** R scripts for use of menopausal hormone therapy and new-onset asthma in menopausal women.

**Additional file 7:** **Figure S1.** Previous cohort studies on use of hormonal contraceptives and new-onset asthma in women. **Figure S2.** Simplified causal directed acyclic graphs to represent the role of body mass index for the effect of use of hormonal contraceptives on new-onset asthma in women.

## Data Availability

The datasets generated and/or analyzed during the current study are not publicly available due to participant consent.

## Abbreviations

CI: Confidence interval
DAGs: Directed acyclic graphs
FCS: Full-conditional specification
MCMC: Markov chain Monte Carlo
MHT: Menopausal hormone therapy
MI: Multiple imputation
OR: Odds ratio
PECOS: Population, Exposure, Comparator, Outcome, and Study design
PI: Posterior interval
STROBE: The Strengthening the Reporting of Observational Studies in Epidemiology
WSAS: West Sweden Asthma Study
