# Supplementary material for: Exogenous female sex steroid hormones and new-onset asthma in women: a matched case–control study
Source: BMC Med. 2023 Sep 4;21:337. doi: 10.1186/s12916-023-03038-8 (PMC10478448; doi:10.1186/s12916-023-03038-8)
Supplement: Supplementary file 5 — Additional file 5. R scripts for use of hormonal contraceptives and new-onset asthma in women. [file 12916_2023_3038_MOESM5_ESM.pdf]

Hormonal contraceptives and new-onset asthma in women

Guo-Qiang Zhang (guo-qiang.zhang@gu.se), Rani Basna, Maya B. Mathur

1. Load Packages

```r
library(readxl)
library(haven)
library(dplyr)
library(ggplot2)
library(naniar)
library(Epi)
library(finalfit)
library(mice)
library(rstan)
library(rstanarm)
library(bayesplot)
library(bayesrules)
library(data.table)
library(EValue)
```

2. Import Dataset

```r
# matched dataset
d1 <- read_xlsx("/Users/xzhagu/Library/Mobile Documents/com~apple~CloudDocs/Documents/Sex Hormone and Asthma in Women/Sex hormones and adult-onset asthma in women/R_sex hormones and adult-onset asthma in women/Matched_dataset_sex_hormone_adult_onset_asthma_in_women/Matched_dataset_GQ.xlsx")
table(d1$incident_asthma) # 72 incident asthma cases and 602 matched controls

# WSAS I sex hormone data
d2 <- read_sav("/Users/xzhagu/Library/Mobile Documents/com~apple~CloudDocs/Documents/Sex Hormone and Asthma in Women/WSAS/WSAS Datasets/Sex hormone questionnaire data in women/WSAS_I_sex_hormone_questionnaire_data.sav")

# other data
d3 <- read_sav("/Users/xzhagu/Library/Mobile Documents/com~apple~CloudDocs/Documents/Sex Hormone and Asthma in Women/WSAS/WSAS Datasets/Full datasets_WSAS I & II/Postal questionnaire 2008 WSAS I/probably-full-raw-questionnaire-data-wsas-2008.sav") # postal questionnaire data in 2008
d4 <- read_sav("/Users/xzhagu/Library/Mobile Documents/com~apple~CloudDocs/Documents/Sex Hormone and Asthma in Women/WSAS/WSAS Datasets/Full datasets_WSAS I & II/Postal questionnaire 2016 WSAS I & II/probably-full-raw-questionnaire-data-wsas-2016.sav") # postal questionnaire data in 2016

# merge datasets
d5 <- merge(d1, d2[,c(1,4,26,27,29:32,7,59,64,67)], by.x = "person_id", by.y = "ID")
table(d5$incident_asthma) # 72 cases and 281 matched controls answered the Women's Questionnaire survey
d6 <- merge(d5, d3[,c(1,143)], by.x = "person_id", by.y = "ID")
d7 <- merge(d6, d4[,c(1,12,88)], by.x = "person_id", by.y = "ID")

# create/rename variables
str(d7)
d7$subclass <- as.factor(d7$subclass) # matched set

d7$asthma <- factor(d7$incident_asthma, levels = c("non-asthmatic", "asthmatic")) # asthma
d7$age_at_asthma_diagnosis <- as.numeric(ifelse(d7$incident_asthma=="asthmatic", d7$F3B, 0)) # age at asthma diagnosis

d7$hormonal_contraceptives <- d7$Q9 # ever use of hormonal contraceptives
d7$hormonal_contraceptives[d7$hormonal_contraceptives==-99] <- NA
d7$hormonal_contraceptives <- as.factor(d7$hormonal_contraceptives)
d7$age_of_starting_contraceptives <- ifelse(d7$hormonal_contraceptives==2, d7$Q9_1,
                                      ifelse(d7$hormonal_contraceptives==1, 0,
                                             NA)) # age when starting hormonal contraceptives

d7$pill <- as.factor(ifelse(d7$Q9_3_1>0, 2, ifelse(is.na(d7$hormonal_contraceptives), NA, 1))) # pill
d7$injection <- as.factor(ifelse(d7$Q9_3_2>0, 2, ifelse(is.na(d7$hormonal_contraceptives), NA, 1))) # injection
d7$vaginal_ring <- as.factor(ifelse(d7$Q9_3_3>0, 2, ifelse(is.na(d7$hormonal_contraceptives), NA, 1))) # vaginal ring
d7$implant <- as.factor(ifelse(d7$Q9_3_4>0, 2, ifelse(is.na(d7$hormonal_contraceptives), NA, 1))) # implant under the skin

d7$age_2008 <- d7$age_2016-8 # age at enrolment in 2008

d7$menarche <- as.numeric(d7$Q2) # age at menarche

d7$Q17 <- as.numeric(d7$Q17) # level of education in 2016
d7$education_2016 <- as.factor(ifelse(d7$Q17 <= 3, "less than high school",
                               ifelse(d7$Q17 <= 5, "high school", "tertiary level")))


d7$gynecological_condition <- as.factor(ifelse(d7$Q5 == 2 | d7$Q12 == 2 | d7$Q13_2 == 2 | d7$Q14 ==2, 2, 1)) # gynecological conditions

d8 <- subset(d7, select = -c(person_id, Q2, Q9, Q9_1, Q9_3_1, Q9_3_2, Q9_3_3, Q9_3_4, Ålder, F3B, Q17, Q5, Q12, Q13_2, Q14))
str(d8)
```

3. Visualize Data

```r
# age at enrolment in 2008
summary(d8$age_2008)
{
  barplot(table(d8$age_2008),
          xlab = "Age at baseline (year)",
          ylab = "Number of women",
          ylim = c(0, 20),
          axes = FALSE
          )
  axis(2, at = seq( 0, 20, 5), las = 1)
}

# age of starting hormonal contraceptives
summary(d8[d8$hormonal_contraceptives==2 & !is.na(d8$hormonal_contraceptives),]$age_of_starting_contraceptives) # 5 missing
{
  barplot(table(d8[d8$hormonal_contraceptives==2 & !is.na(d8$hormonal_contraceptives),]$age_of_starting_contraceptives),
          xlab = "Age when starting hormonal contraceptives (year)",
          ylab = "Number of women",
          ylim = c(0, 40),
          axes = FALSE
          )
  axis(2, at = seq( 0, 40, 10), las = 1)
}

# age at asthma diagnosis
summary(d8[d8$incident_asthma == "asthmatic",]$age_at_asthma_diagnosis) # 40 missing
{
  barplot(table(d8[d8$incident_asthma == "asthmatic",]$age_at_asthma_diagnosis),
          xlab = "Age at asthma diagnosis (year)",
          ylab = "Number of women",
          axes = FALSE
          )
  axis(2, at = seq(0, 3, 1), las = 1)
}
```

4. Complete-Case Analysis

4.1. Frequentist conditional logistic regression

```r
# among all women
m1 <- clogistic(asthma ~ hormonal_contraceptives + menarche + education_2016 + gynecological_condition, strata = subclass, data = d8)
m1
round(exp(m1$coefficients), 2)
round(exp(confint(m1)), 2)

# exploratory subgroup analysis by age at baseline in 2008
# example R script for women aged >= 25 years
m2 <- clogistic(asthma ~ hormonal_contraceptives + menarche + education_2016 + gynecological_condition, strata = subclass, data = d8[d8$age_2008>=25,])
m2
round(exp(m2$coefficients), 2)
round(exp(confint(m2)), 2)
```

5. Multiple Imputation Analysis

5.1. Obtain incomplete dataset

```r
d_incomplete <- read_xlsx("/Users/xzhagu/Library/Mobile Documents/com~apple~CloudDocs/Documents/Sex Hormone and Asthma in Women/Sex hormones and adult-onset asthma in women/R_sex hormones and adult-onset asthma in women/Matched_dataset_sex_hormone_adult_onset_asthma_in_women/all cases with matched controls.xlsx")
table(d_incomplete$incident_asthma) # 114 cases with 717 matched controls
n_distinct(d_incomplete$subclass) # 89 matched strata

# obtain relevant variables
d9 <- merge(
  merge(
    merge(
      d_incomplete, d2[,c(1,4,26,7,59,64,67,48)], by.x = "person_id", by.y = "ID", all.x = TRUE),
    d3[,c(1,108,111,112,141,143)], by.x = "person_id", by.y = "ID", all.x = TRUE),
  d4[,c(1,88)], by.x = "person_id", by.y = "ID", all.x = TRUE)

str(d9)
d9$subclass <- as.factor(d9$subclass)
d9$age_2008 <- d9$age_2016-8 # age at baseline in 2008
d9$residence_2008 <- as.factor(d9$residence) # place of residence in 2008
d9$smoking_2016 <- as.factor(d9$smoking_2016) # smoking status

d9$asthma <- factor(d9$incident_asthma, levels = c("non-asthmatic", "asthmatic")) # asthma

d9$hormonal_contraceptives <- d9$Q9 # ever use of hormonal contraceptives
d9$hormonal_contraceptives[d9$hormonal_contraceptives==-99] <- NA
d9$hormonal_contraceptives <- as.factor(d9$hormonal_contraceptives)

d9$age_at_menarche <- as.numeric(d9$Q2) # age at menarche

d9$gynecological_condition <- as.factor(ifelse(d9$Q5 == 2 | d9$Q12 == 2 | d9$Q13_2 == 2 | d9$Q14 ==2, 2, 1)) # gynecological conditions

d9$number_of_live_births <- as.numeric(d9$Q11_1) # number of live births

d9$occupation <- as.factor(ifelse(d9$Q17.x=="None", NA, d9$Q17.x)) # occupation

d9$height <- as.numeric(d9$Q19) # height (cm)
d9$weight <- as.numeric(d9$Q20) # weight (kg)
d9$bmi <- d9$weight/(d9$height/100)^2 # body mass index

d9$exercise <- factor(d9$Q39, levels = c(1:6)) # physical exercise

d9$education_2016 <- factor(d9$Q17.y, levels = c(1:7)) # level of education in 2016

str(d9)
d10 <- d9 %>% select(subclass, residence_2008, smoking_2016, age_2016, age_2008, asthma, hormonal_contraceptives, age_at_menarche, gynecological_condition, education_2016, number_of_live_births, occupation, height, weight, bmi, exercise)
str(d10)
```

5.2. Visualize missing pattern and select predictors

```r
# identify missing variables
vis_miss(d10)
n_miss(d10)
miss_var_summary(d10)
md.pattern(d10)

# select auxiliary variables for the incomplete variables in the analysis model (use of hormonal contraceptives, age at menarche, gynecological conditions, and level of education)
# check whether potential auxiliary variables are correlated with the incomplete variables
plot(d10$hormonal_contraceptives, d10$number_of_live_births) # uncorrelated
plot(d10$hormonal_contraceptives, d10$occupation)
plot(d10$hormonal_contraceptives, d10$bmi)
plot(d10$hormonal_contraceptives, d10$exercise)

cor(d10$age_at_menarche, d10$number_of_live_births, use = "pair") # Pearson correlation -0.02
plot(d10$occupation, d10$age_at_menarche)
cor(d10$bmi, d10$age_at_menarche, use = "pair") # Pearson correlation -0.26
plot(d10$exercise, d10$age_at_menarche) # uncorrelated

plot(d10$gynecological_condition, d10$number_of_live_births) # uncorrelated
plot(d10$gynecological_condition, d10$occupation)
plot(d10$gynecological_condition, d10$bmi)
plot(d10$gynecological_condition, d10$exercise)

plot(d10$education_2016, d10$number_of_live_births)
plot(d10$occupation, d10$education_2016)
plot(d10$education_2016, d10$bmi)
plot(d10$exercise, d10$education_2016)

# check whether potential auxiliary variables are related to missingness in the incomplete variables
str(d10)
{
  explanatory =  c("age_at_menarche", "gynecological_condition", "education_2016", "number_of_live_births", "occupation", "bmi", "exercise")
  dependent = "hormonal_contraceptives"
  d10 %>%  missing_pairs(dependent, explanatory, position = "fill", showYAxisPlotLabels = TRUE)
}

# finally, include body mass index, occupation and physical exercise as auxiliary variables for the incomplete variables
d11 <- d10 %>% select(subclass, residence_2008, smoking_2016, age_2016, age_2008, asthma, hormonal_contraceptives, age_at_menarche, gynecological_condition, education_2016, occupation, height, weight, bmi, exercise)
```

5.3. Generate multiple imputation

```r
# perform a dry run
{
  ini <- mice(d11,
              defaultMethod = c("pmm", "logreg", "polyreg", "polr"),
              maxit = 0,
              print = FALSE)
  meth <- ini$meth
  meth["bmi"] <- "~I(weight/(height/100)^2)" # bmi is imputed by passive imputation, so that the imputed values are consistent with (imputed) values of height and weight
  meth
  pred <- ini$pred
  pred[c("subclass", "residence_2008", "smoking_2016", "age_2016", "age_2008", "asthma"),] <- 0 # no missing values in these variables
  pred[, c("subclass", "age_2008")] <- 0
  pred[c("weight", "height"), "bmi"] <- 0 # avoid weight and height are imputed from bmi
  pred[c("hormonal_contraceptives", "age_at_menarche", "gynecological_condition", "education_2016", "occupation", "exercise"), c("weight", "height")] <- 0 # make sure variables use either weight/height or bmi as predictor
  pred
  vis <- ini$vis
  vis <- append(vis, vis[14], 12) # bmi is visited both after height and after weight
  vis <- c(vis[10], vis[11], vis[12], vis[13], vis[14], vis[15], vis[16], vis[7], vis[8], vis[9] ) # add age at menarche to the end of the visiting sequence
  vis
  ini$nmis # shows number of missing values per variable
}

# multiple imputation
imp <- mice(d11,
            m = 100, # age at menarche has the most missingness (≈ 60%)
            pred = pred,
            vis = vis,
            meth = meth,
            maxit = 10,
            seed = 123,
            print = FALSE)
```

5.4. Validate imputations

```r
plot(imp) # check convergence
plot(imp, c("hormonal_contraceptives", "age_at_menarche", "gynecological_condition", "education_2016"))

# visualize the distributions of imputed data
com <- complete(imp, action = 'long', include = TRUE)
str(com)
com$education <- factor(ifelse(as.numeric(com$education_2016) <= 3, "less than high school",
                               ifelse(as.numeric(com$education_2016) <= 5, "high school", "tertiary level")),
                        levels = c("less than high school", "high school", "tertiary level"))

ggplot(data = com[!is.na(com$hormonal_contraceptives),], # hormonal contraceptives
       aes(fill = hormonal_contraceptives, x = .imp )) +
  geom_bar(position = "fill") +
  scale_y_continuous( labels = scales::percent ) +
  xlab("Imputation number") +
  ylab("Percentage") +
  scale_fill_discrete(name = "Use of hormonal contraceptives", labels = c("No", "Yes")) +
  theme(legend.justification = "bottom")

densityplot(imp, ~age_at_menarche, thicker = 8) # age at menarche

ggplot(data = com[!is.na(com$gynecological_condition),], # gynecological condition
       aes(fill = gynecological_condition, x = .imp )) +
  geom_bar(position = "fill") +
  scale_y_continuous(labels = scales::percent) +
  xlab("Imputation number") +
  ylab("Percentage") +
  scale_fill_discrete(name = "Gynecological condition", labels = c("No", "Yes")) +
  theme(legend.justification = "bottom")

ggplot(data = com[!is.na(com$education),], # level of education
       aes(fill = education, x = .imp )) +
  geom_bar(position = "fill") +
  scale_y_continuous(labels = scales::percent) +
  xlab("Imputation number") +
  ylab("Percentage") +
  scale_fill_discrete(name = "Level of education", labels = c("Less than high school", "High school", "Tertiary level")) +
  theme(legend.justification = "bottom")

setDT(com) # check for the distributions of auxiliary variables (BMI as an example)
com[com$.imp!=0, as.list(summary(bmi)), by = .imp]
```

5.5. Frequentist conditional logistic regression

```r
# prepare multiply imputed datasets for analysis
head(com)
str(com)

imp.final <- as.mids(com)

# analyze multiply imputed datasets
fit <- with(imp.final, clogistic(asthma ~ hormonal_contraceptives + age_at_menarche + education + gynecological_condition, strata = subclass))
print(fit$analyses[[1]]) # insepect the analysis results, for example, of the first imputed dataset

# combine estimates using Rubin's rules
{
  coef <- matrix(nrow = 100, ncol = 5)
  var <- matrix(nrow = 100, ncol = 5)

  for(k in 1:100){
    coef[k,]<-fit$analyses[[k]]$coef
    var[k,]<-diag(fit$analyses[[k]]$var)
    }
}

summary(com[com$.imp==1,]$asthma)
pool <- pool.scalar(coef[,1],var[,1], n = nrow(com[com$.imp==1,]), k = 2)

pool$qbar # pooled coefficient
sqrt(pool$t) # standard error of pooled coefficient

matrix( round(exp(data.frame(pool$qbar, pool$qbar-sqrt(pool$t)*1.96, pool$qbar+sqrt(pool$t)*1.96)), 2),
        nrow = 1,
        ncol = 3,
        dimnames = list( "", c( "Pooled.odds.ratio", "95%.CI.lower", "95%.CI.upper" ) ) )

# plot the range of odds ratios across imputed datasets
plot(exp(coef[,1]),
     type = "l",
     col = "steelblue3",
     lwd = 1.5,
     xlab = "Imputation number",
     ylab = "Odds ratio")

# subgroup analysis by age at baseline in 2008
# R script example for age group >= 25 years
{
  x <- com[com$age_2008 >= 25,] # indicate age group
  fit_1 <- with(as.mids(x),
                clogistic(asthma ~ hormonal_contraceptives + age_at_menarche + education + gynecological_condition, strata = subclass))

  coef_1 <- matrix(nrow = 100, ncol = 5)
  var_1 <- matrix(nrow = 100, ncol = 5)

  for(k in 1:100){
  coef_1[k,]<-fit_1$analyses[[k]]$coef
  var_1[k,]<-diag(fit_1$analyses[[k]]$var)
  }
}

summary(x[x$.imp==1,]$asthma)
pool_1 <- pool.scalar(coef_1[,1],var_1[,1], n = nrow(x[x$.imp==1,]), k = 2)

matrix( round(exp(data.frame(pool_1$qbar, pool_1$qbar-sqrt(pool_1$t)*1.96, pool_1$qbar+sqrt(pool_1$t)*1.96)), 2),
        nrow = 1,
        ncol = 3,
        dimnames = list( ">=25 years", c( "Pooled.odds.ratio", "95%.CI.lower", "95%.CI.upper" ) ) )
```

5.6. Bayesian conditional logistic regression

5.6.1. Specify prior probability distribution

```r
# prior distribution for the effect of use of hormonal contraceptives on new-onset asthma in women
mean <- (log(0.6) + log(1.0))/2 # mean (on log odds ratio scale)
sd_original <- (log(1.0) - log(0.6))/(1.28*2) # standard deviation (on log odds ratio scale)
sd_1 <- (log(1.0) - log(0.6))/(1.04*2)
sd_2 <- (log(1.0) - log(0.6))/(0.84*2)
sd_3 <- (log(1.0) - log(0.6))/(0.67*2)

# plot prior distributions
{
  base <- ggplot() + xlim(-1.5, 1)
  base +
    stat_function(aes(colour = "Origional Prior"), fun = dnorm, n = 1001, args = list(mean = mean, sd = sd_original),
                  geom = "area", fill = "steelblue", alpha = 0.8) +
    stat_function(aes(colour = "Alternative Prior 1"), fun = dnorm, n = 1001, args = list(mean = mean, sd = sd_1),
                  geom = "area", fill = "lightblue", alpha = 0.6) +
    stat_function(aes(colour = "Alternative Prior 2"), fun = dnorm, n = 1001, args = list(mean = mean, sd = sd_2),
                  geom = "area", fill = "lightyellow", alpha = 0.6) +
    stat_function(aes(colour = "Alternative Prior 3"), fun = dnorm, n = 1001, args = list(mean = mean, sd = sd_3),
                  geom = "area", fill = "grey", alpha = 0.6) +
    labs(x = "Log Odds Ratio", y = "Probability Density", title = "Prior Probability Distribution",
         subtitle = "For the effect of use of hormonal contraceptives on new-onset asthma") +
    scale_color_manual(values = c("black", "black", "black", "black")) +
    guides(color = guide_legend(override.aes = list(fill = c("lightblue", "lightyellow", "grey", "steelblue"),
                                                    alpha = c(0.6, 0.6, 0.6, 0.8), color = NA))) +
    theme(legend.position = "bottom",
          legend.title = element_blank(),
          panel.background = element_blank(),
          panel.grid.major = element_blank(),
          panel.grid.minor = element_blank(),
          panel.border = element_blank(),
          plot.title = element_text(size = 18, face = "bold"),
          plot.subtitle = element_text(size = 18),
          axis.title.x = element_text(size = 14),
          axis.title.y = element_text(size = 14),
          axis.text = element_text(size = 14),
          legend.text = element_text(size = 12),
          text = element_text(family = "Arial"),
          axis.line = element_line(color='black')
    )
}
```

5.6.2. Bayesian conditional logistic regression model

```r
com$asthma_numeric <- ifelse(com$asthma=="asthmatic", 1, 0)

# obtain weakly informative priors for adjustment variables
post_ini <- stan_clogit(asthma_numeric ~ hormonal_contraceptives + age_at_menarche + education + gynecological_condition,
                        data = arrange(com[com$.imp==1,], subclass),
                        strata = subclass,
                        algorithm = "sampling",
                        chains = 1,
                        iter = 10*2,
                        refresh = 0)

prior_summary(post_ini)
prior_original <- normal(location = c(mean, 0, 0, 0, 0), scale = c(sd_original, 1.71, 5.56, 5.03, 5.04), autoscale = FALSE)

# fit Bayesian conditional logistic regression model to multiply imputed datasets
{
  n_dataset <- 100

  # extract MCMC draws from multiply imputed datasets
  posterior <- matrix(nrow = 4000, ncol = n_dataset)
  for(k in 1:n_dataset){
    posterior[,k] <- as.matrix(stan_clogit(
      asthma_numeric ~ hormonal_contraceptives + age_at_menarche + education + gynecological_condition,
      data = arrange(com[com$.imp==k,], subclass),
      strata = subclass,
      prior = prior_original,
      algorithm = "sampling",
      chains = 4,
      iter = 1000*2,
      refresh = 0,
      seed = 123),
      pars = "hormonal_contraceptives2")
    }
  } # It took more than one hour to run!

dim(posterior)

{
  # convert matrix into array
  r.names <- c(1:4000)
  m.names <- "hormonal contraceptives"
  c.names <- matrix(nrow = 100, ncol = 1)
  for(k in 1:ncol(posterior)){
    c.names[k,] <- paste("chain:", k, sep = "")
    }

  posterior_array <- array(posterior,
                           dim = c(4000, 100, 1),
                           dimnames = list(r.names, c.names, m.names))
}

# plot posterior distribution
{
  color_scheme_set("blue")
  mcmc_areas(posterior_array, point_est = "median", prob = 0.95) +
    scale_y_discrete(labels = c("hormonal contraceptives" = ""), expand = expansion(add = c(0, 0.8))) +
    labs(x = "Log Odds Ratio (95% PI)", y = "Probability Density") +
    theme(axis.title.x = element_text(size = 12),
          axis.title.y = element_text(size = 12),
          axis.text = element_text(size = 12),
          legend.text = element_text(size = 10),
          text = element_text(family = "Arial"),
          axis.line = element_line(color='black'),
          axis.ticks.y = element_blank())
}

# calculate mean and SD
round(mean(posterior), 2)
round(sd(posterior), 2)

# calculate median and 95% posterior interval (Equal-Tailed Interval)
round(exp(quantile(posterior, probs = c(.025, 0.500, 0.975))), 2) # on odds ratio scale

# calculate the probability of regression coefficient >0 (i.e., odds ratio >1)
round(mean(posterior > 0), 3)

# plot posterior distribution from each imputed dataset
mcmc_dens_overlay(posterior_array) +
  labs(x = "Log Odds Ratio", y = "Probability Density",
       title = "Posterior Probability Distribution from Each Imputed Dataset",
       subtitle = "For the effect of use of hormonal contraceptives on new-onset asthma") +
  theme(plot.title = element_text(size = 18, face = "bold"),
        plot.subtitle = element_text(size = 18),
        axis.title.x = element_text(size = 14),
        axis.title.y = element_text(size = 14),
        axis.text = element_text(size = 12),
        legend.position = "none",
        text = element_text(family = "Arial"))
```

5.6.3. Check sampling quality

```r
# check for the first imputated dataset
post_1 <- stan_clogit(asthma_numeric ~ hormonal_contraceptives + age_at_menarche + education + gynecological_condition,
                      data = arrange(com[com$.imp==1,], subclass),
                      strata = subclass,
                      prior = prior_original,
                      algorithm = "sampling",
                      chains = 4,
                      iter = 1000*2,
                      refresh = 0,
                      seed = 123) # fit the Bayesian model

prior_summary(post_1) # check priors

posterior_1 <- as.array(post_1, pars = "hormonal_contraceptives2") # extract MCMC draws

# posterior approximations from each chain
{
  color_scheme_set("mix-brightblue-gray")
  mcmc_dens_overlay(posterior_1) +
    labs(x = "Log Odds Ratio",
         y = "Probability Density",
         title = "Posterior Probability Distribution from the First Imputed Dataset",
         subtitle = "For the effect of use of hormonal contraceptives on new-onset asthma") +
    theme(plot.title = element_text(size = 18, face = "bold"),
          plot.subtitle = element_text(size = 18),
          axis.title.x = element_text(size = 14),
          axis.title.y = element_text(size = 14),
          axis.text = element_text(size = 12),
          text = element_text(family = "Arial"))
}

# trace plot
{
  color_scheme_set("mix-brightblue-gray")
  mcmc_trace(posterior_1) +
    labs(x = "Post-Warmup Iteration",
         y = "Log Odds Ratio",
         title = "Trace Plot from the First Imputed Dataset",
         subtitle = "For the effect of use of hormonal contraceptives on new-onset asthma") +
    theme(plot.title = element_text(size = 18, face = "bold"),
          plot.subtitle = element_text(size = 18),
          axis.title.x = element_text(size = 14),
          axis.title.y = element_text(size = 14),
          axis.text = element_text(size = 12),
          text = element_text(family = "Arial"))
}


# Rhat
summary(post_1)[, "Rhat"]
any(summary(post_1)[, "Rhat"] >= 1.1)

# effective sample size
summary(post_1)[, "n_eff"]
any(summary(post_1)[, "n_eff"] < 1000)

# more detailed results can be viewed by
# launch_shinystan(post_1)
```

5.6.4. Prior sensitivity analysis

```r
########## Alternative Prior 1 ##########
prior_alternative_1 <- normal(location = c(mean, 0, 0, 0, 0), scale = c(sd_1, 1.71, 5.56, 5.03, 5.04), autoscale = FALSE)

{
# extract MCMC draws from multiply imputed datasets
  posterior_alternative_1 <- matrix(nrow = 4000, ncol = n_dataset)
  for(k in 1:n_dataset){
    posterior_alternative_1[,k] <- as.matrix(stan_clogit(
      asthma_numeric ~ hormonal_contraceptives + age_at_menarche + education + gynecological_condition,
      data = arrange(com[com$.imp==k,], subclass),
      strata = subclass,
      prior = prior_alternative_1,
      algorithm = "sampling",
      chains = 4,
      iter = 1000*2,
      refresh = 0,
      seed = 123),
      pars = "hormonal_contraceptives2")
    }
  }

# calculate mean and SD
round(mean(posterior_alternative_1), 2)
round(sd(posterior_alternative_1), 2)

# calculate median and 95% posterior interval (Equal-Tailed Interval)
round(exp(quantile(posterior_alternative_1, probs = c(.025, 0.500, 0.975))), 2) # on odds ratio scale

# calculate the probability of regression coefficient >0 (i.e., odds ratio >1)
round(mean(posterior_alternative_1 > 0), 3)


########## Alternative Prior 2 ##########
prior_alternative_2 <- normal(location = c(mean, 0, 0, 0, 0), scale = c(sd_2, 1.71, 5.56, 5.03, 5.04), autoscale = FALSE)

{
# extract MCMC draws from multiply imputed datasets
  posterior_alternative_2 <- matrix(nrow = 4000, ncol = n_dataset)
  for(k in 1:n_dataset){
    posterior_alternative_2[,k] <- as.matrix(stan_clogit(
      asthma_numeric ~ hormonal_contraceptives + age_at_menarche + education + gynecological_condition,
      data = arrange(com[com$.imp==k,], subclass),
      strata = subclass,
      prior = prior_alternative_2,
      algorithm = "sampling",
      chains = 4,
      iter = 1000*2,
      refresh = 0,
      seed = 123),
      pars = "hormonal_contraceptives2")
    }
  }

# calculate mean and SD
round(mean(posterior_alternative_2), 2)
round(sd(posterior_alternative_2), 2)

# calculate median and 95% posterior interval (Equal-Tailed Interval)
round(exp(quantile(posterior_alternative_2, probs = c(.025, 0.500, 0.975))), 2) # on odds ratio scale

# calculate the probability of regression coefficient >0 (i.e., odds ratio >1)
round(mean(posterior_alternative_2 > 0), 3)


########## Alternative Prior 3 ##########
prior_alternative_3 <- normal(location = c(mean, 0, 0, 0, 0), scale = c(sd_3, 1.71, 5.56, 5.03, 5.04), autoscale = FALSE)

{
# extract MCMC draws from multiply imputed datasets
  posterior_alternative_3 <- matrix(nrow = 4000, ncol = n_dataset)
  for(k in 1:n_dataset){
    posterior_alternative_3[,k] <- as.matrix(stan_clogit(
      asthma_numeric ~ hormonal_contraceptives + age_at_menarche + education + gynecological_condition,
      data = arrange(com[com$.imp==k,], subclass),
      strata = subclass,
      prior = prior_alternative_3,
      algorithm = "sampling",
      chains = 4,
      iter = 1000*2,
      refresh = 0,
      seed = 123),
      pars = "hormonal_contraceptives2")
    }
  }

# calculate mean and SD
round(mean(posterior_alternative_3), 2)
round(sd(posterior_alternative_3), 2)

# calculate median and 95% posterior interval (Equal-Tailed Interval)
round(exp(quantile(posterior_alternative_3, probs = c(.025, 0.500, 0.975))), 2) # on odds ratio scale

# calculate the probability of regression coefficient >0 (i.e., odds ratio >1)
round(mean(posterior_alternative_3 > 0), 3)


########## Flat Prior ##########
{
# extract MCMC draws from multiply imputed datasets
  posterior_flat <- matrix(nrow = 4000, ncol = n_dataset)
  for(k in 1:n_dataset){
    posterior_flat[,k] <- as.matrix(stan_clogit(
      asthma_numeric ~ hormonal_contraceptives + age_at_menarche + education + gynecological_condition,
      data = arrange(com[com$.imp==k,], subclass),
      strata = subclass,
      prior = NULL,
      algorithm = "sampling",
      chains = 4,
      iter = 1000*2,
      refresh = 0,
      seed = 123),
      pars = "hormonal_contraceptives2")
    }
  }

# calculate mean and SD
round(mean(posterior_flat), 2)
round(sd(posterior_flat), 2)

# calculate median and 95% posterior interval (Equal-Tailed Interval)
round(exp(quantile(posterior_flat, probs = c(.025, 0.500, 0.975))), 2) # on odds ratio scale

# calculate the probability of regression coefficient >0 (i.e., odds ratio >1)
round(mean(posterior_flat > 0), 3)
```

5.6.5. Plot for prior sensitivity analysis

```r
posterior_all <- as.data.frame(unlist(cbind(
  cbind(
    cbind(
      cbind(data.frame(unlist(as.data.frame(posterior))),
            data.frame(unlist(as.data.frame(posterior_alternative_1)))),
      data.frame(unlist(as.data.frame(posterior_alternative_2)))),
    data.frame(unlist(as.data.frame(posterior_alternative_3)))),
  data.frame(unlist(as.data.frame(posterior_flat))))))

posterior_all$row_number <- 1:nrow(posterior_all)
posterior_all$cat <- ifelse(posterior_all$row_number<=400000*1, "original_prior",
                            ifelse(posterior_all$row_number<=400000*2, "alternative_prior_1",
                                   ifelse(posterior_all$row_number<=400000*3, "alternative_prior_2",
                                          ifelse(posterior_all$row_number<=400000*4, "alternative_prior_3",
                                                 "flat_prior"))))
colnames(posterior_all)[1] <- "value"
str(posterior_all)
posterior_all$cat <- factor(posterior_all$cat, levels = c("flat_prior", "original_prior", "alternative_prior_1", "alternative_prior_2",
                                                          "alternative_prior_3"))
table(posterior_all$cat)

ggplot(posterior_all, aes(x = value, fill = cat)) +
  geom_density(alpha=0.6) +
  scale_fill_manual(values=c("original_prior"="steelblue",
                             "alternative_prior_1"="lightblue",
                             "alternative_prior_2"="lightyellow",
                             "alternative_prior_3"="grey",
                             "flat_prior"="darkcyan"),
                    labels = c("Original Prior", "Alternative Prior 1", "Alternative Prior 2", "Alternative Prior 3", "Flat Prior")) +
  geom_vline(xintercept = 0, colour = "gray") +
  coord_cartesian(xlim = c(-1.5, 1.5)) +
  scale_y_discrete(expand = expansion(add = c(0, 0.1))) +
  labs(x = "Log Odds Ratio", y = "Probability Density", title = "Prior Sensitivity Analysis",
       subtitle = "For the effect of use of hormonal contraceptives on new-onset asthma") +
  theme(legend.position = "bottom",
        legend.title = element_blank(),
        panel.background = element_blank(),
        panel.grid.major = element_blank(),
```

```
        panel.grid.minor = element_blank(),
        panel.border = element_blank(),
        plot.title = element_text(size = 18, face = "bold"),
        plot.subtitle = element_text(size = 18),
        axis.title.x = element_text(size = 14),
        axis.title.y = element_text(size = 14),
        axis.text = element_text(size = 14),
        legend.text = element_text(size = 12),
        text = element_text(family = "Arial"),
        axis.line = element_line(color='black'))
```

6. Sensitivity Analysis for Residual Confounding

Hide

```
effect.estimate <-  # on odds ratio scale

# if the outcome is relatively rare (< 15% at the end of follow-up)
evalues.OR(est = effect.estimate, rare = TRUE)

# if the outcome is common (>= 15% at the end of follow-up)
evalues.OR(est = effect.estimate, rare = FALSE)
```

Hide

```
# some helper scripts
save.image(file = "Global_Environment_HC.RData") # save work environment
load(file = "Global_Environment_HC.RData") # load work environment

# record the versions of R packages used
library(renv)
renv::init()
renv::snapshot() # update renv.lock lockfile
```
