# Supplementary material for: Exogenous female sex steroid hormones and new-onset asthma in women: a matched case–control study
Source: BMC Med. 2023 Sep 4;21:337. doi: 10.1186/s12916-023-03038-8 (PMC10478448; doi:10.1186/s12916-023-03038-8)
Supplement: Supplementary file 4 — Additional file 4: Section 1. Supplementary methods and results for the Bayesian analysis of hormonal contraceptives and new-onset asthma in women. Table S1. Posterior estimates for the original and alternative priors for use of hormonal contraceptives and new-onset asthma in women. Figure S1. The original and alternative prior distributions. Figure S2. Posterior distributions estimated from each multiply imputed dataset (m = 100). Figure S3. Posterior distributions estimated from each Markov chain in the first imputed dataset. Figure S4. Trace plot from the first imputed dataset. Figure S5. Posterior distributions for the original and alternative priors. Section 2: Supplementary methods and results for the Bayesian analysis of menopausal hormone therapy and new-onset asthma in menopausal women. Table S2. Posterior estimates for the original and alternative priors for use of menopausal hormone therapy and new-onset asthma in menopausal women. Figure S6. The original and alternative prior distributions. Figure S7. Posterior distributions estimated from each multiply imputed dataset (m = 100). Figure S8. Posterior distributions estimated from each Markov chain in the first imputed dataset. Figure S9. Trace plot from the first imputed dataset. Figure S10. Posterior distributions for the original and alternative priors. [file 12916_2023_3038_MOESM4_ESM.pdf]

Additional File 4
Supplementary Methods and Results
# – the *Bayesian* approach

The Additional file 4 contains the supplementary methods and results from the Bayesian analysis for:

Guo-Qiang Zhang*, Rani Basna, Maya B. Mathur, Cecilia Lässer, Roxana Mincheva, Linda Ekerljung, Göran Wennergren, Madeleine Rådinger, Bo Lundbäck, Hannu Kankaanranta, Bright I. Nwaru. Exogenous female sex steroid hormones and new-onset asthma in women: a matched case-control study. 2023.

* Corresponding author
Krefting Research Centre, Department of Internal Medicine and Clinical Nutrition, Institute of Medicine, Sahlgrenska Academy, University of Gothenburg, Gothenburg, Sweden
E-mail: guo-qiang.zhang@gu.se

[The supplementary Tables and Figures are attached at the end of ***each section****, and hyperlinked within the text to allow for easy navigation between the text and the supplementary Tables and Figures.]*

Table of Contents

HORMONAL CONTRACEPTIVES AND NEW-ONSET ASTHMA IN WOMEN 3

1. PRIOR PROBABILITY DISTRIBUTION 3
2. POSTERIOR PROBABILITY DISTRIBUTION 3
3. SAMPLING QUALITY CHECK 3
4. PRIOR SENSITIVITY ANALYSIS 4

MENOPAUSAL HORMONE THERAPY AND NEW-ONSET ASTHMA IN MENOPAUSAL WOMEN 8

1. PRIOR PROBABILITY DISTRIBUTION 8
2. POSTERIOR PROBABILITY DISTRIBUTION 8
3. SAMPLING QUALITY CHECK 8
4. PRIOR SENSITIVITY ANALYSIS 9

EREFERENCES 13

Hormonal Contraceptives and New-Onset Asthma in Women

1. Prior Probability Distribution

To our knowledge,[1] three cohort studies[2–4] have investigated the effect of use of hormonal contraceptives on the risk of developing new-onset asthma in women, but reported conflicting results: as illustrated in Additional file 7: Figure S1, an upward trend existed in the effect estimate with increasing baseline age across or within studies; the relative risk increased from 0.27 to 1.57 when the age at baseline increased. As explained in Additional file 2: Sections 2 (paragraph 2) and 5.2, we suspect that selection bias due to selection of women based on baseline asthma status in these studies may be the main explanation for the increase and the reversal of the relative risk with increasing baseline age. This suggests that use of hormonal contraceptives may in fact have a protective effect on new-onset asthma, as opposed to a harmful effect reported in the Nurses' Health Study.[4]

Thus, we chose our *original* prior distribution as follows: based on the national UK cohort study,[3] the largest longitudinal study to date, in which the majority of the reported hazard ratios (HRs) lay between 0.60 and 1.00 depending on the types of hormonal contraceptives and durations of its use, in our study population we set that the *true average effect* of ever use of hormonal contraceptives compared to never use on asthma risk has an 80% probability of lying between 0.60 and 1.00 on odds ratio (OR) scale. Because asthma was relatively uncommon in the UK cohort study ($<$ 15% by the end of follow-up), we approximated ORs by OR $\approx$ HR.[5] Therefore, the original prior normal distribution on log OR scale has a mean of $(\log(0.60) + \log(1.00))/2 \approx -0.26$ and a standard deviation (SD) of $(\log(1.00) - \log(0.60))/(1.28 \times 2) \approx 0.20$.

In addition, we specified *a priori* a set of *alternative* prior distributions, on log OR scale, of the same mean value (≈ -0.26), but a different SD ($\approx 0.25, 0.30\ or\ 0.38$), which respectively corresponds to a normal distribution with a 70%, 60% or 50% probability that the true average effect of use of hormonal contraceptives on asthma risk in our study population lies between 0.60 and 1.00 on OR scale. We also included a flat prior distribution for all parameters in the model. The original and alternative prior distributions are presented in Figure S1.

2. Posterior Probability Distribution

The posterior distribution based on the original prior is presented in **Figure 2B**. The median of the posterior distribution was 1.11 on OR scale, with 95% posterior interval (PI) 0.79–1.55. The probability of OR being greater than 1 was 72.3%. Figure S2 presents the posterior distributions approximated from each multiply imputed dataset.

3. Sampling Quality Check

For the first imputed dataset, the posterior distribution approximated from each Markov chain is presented in Figure S3. For all parameters in the model, the $\hat{R}$ statistic was less than 1.1, and the effective sample size (ESS) was more than 1,000. The trace plot is shown in Figure S4.

4. Prior Sensitivity Analysis

Figure S5 illustrates and compares the posterior distributions across models with different prior specifications. Table S1 summarizes the posterior estimates of each posterior distribution.

Table S1. Posterior estimates for the original and alternative priors for use of hormonal contraceptives and new-onset asthma in women

Prior	Mean[a]	SD[a]	2.5%[b]	50%[b]	97.5%[b]	$P(>0)$
Original prior	0.10	0.17	0.79	1.11	1.55	72.3%
Alternative prior 1	0.20	0.20	0.82	1.22	1.80	84.0%
Alternative prior 2	0.30	0.23	0.86	1.35	2.12	90.7%
Alternative prior 3	0.41	0.26	0.91	1.51	2.51	94.4%
Flat prior[c]	0.78	0.37	1.08	2.17	4.64	98.5%

Mean = mean of posterior distribution; SD = standard deviation of posterior distribution; 2.5%–97.5% = 95% posterior central interval; 50% = median of posterior distribution; $P\ (>0)$ = probability that the regression coefficient for use of hormonal contraceptives was greater than 0 (i.e., odds ratio >1). All the analyses were conducted based on the multiply imputed datasets, adjusted for age, place of residence, level of education, age at menarche, gynecological conditions, and tobacco smoking.

[a] On log odds ratio scale.

[b] On odds ratio scale.

[c] Flat priors for all parameters in the model.

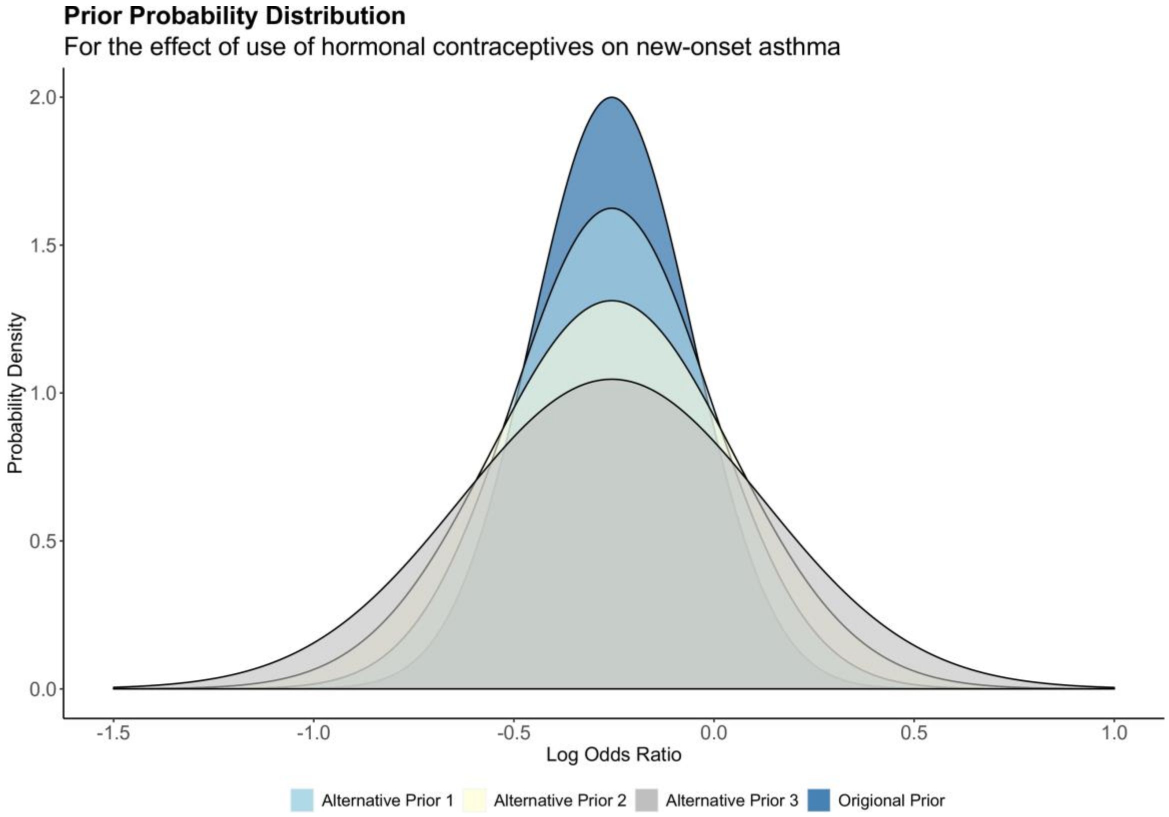

Figure S1. The original and alternative prior distributions.

Original Prior = $N(-0.26, 0.20^2)$; Alternative Prior 1 = $N(-0.26, 0.25^2)$; Alternative Prior 2 = $N(-0.26, 0.30^2)$; Alternative Prior 3 = $N(-0.26, 0.38^2)$.

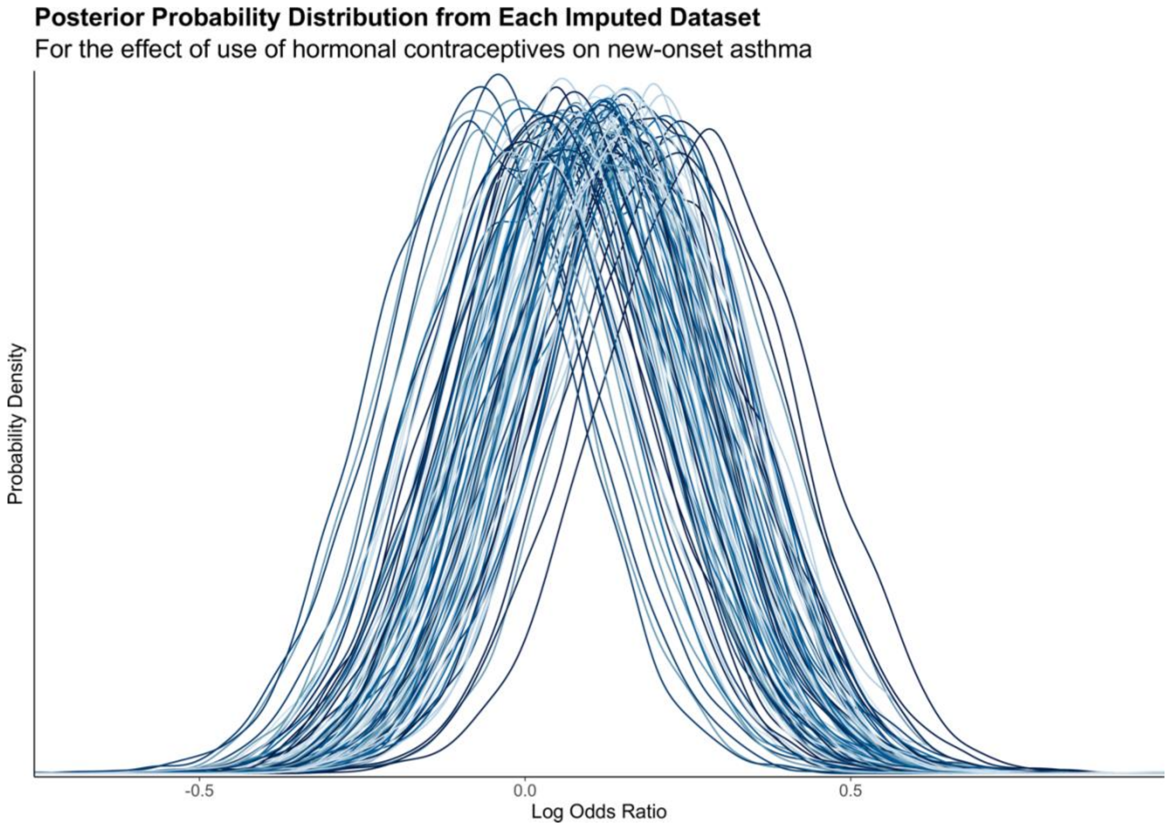

Figure S2. Posterior distributions estimated from each multiply imputed dataset (m = 100).

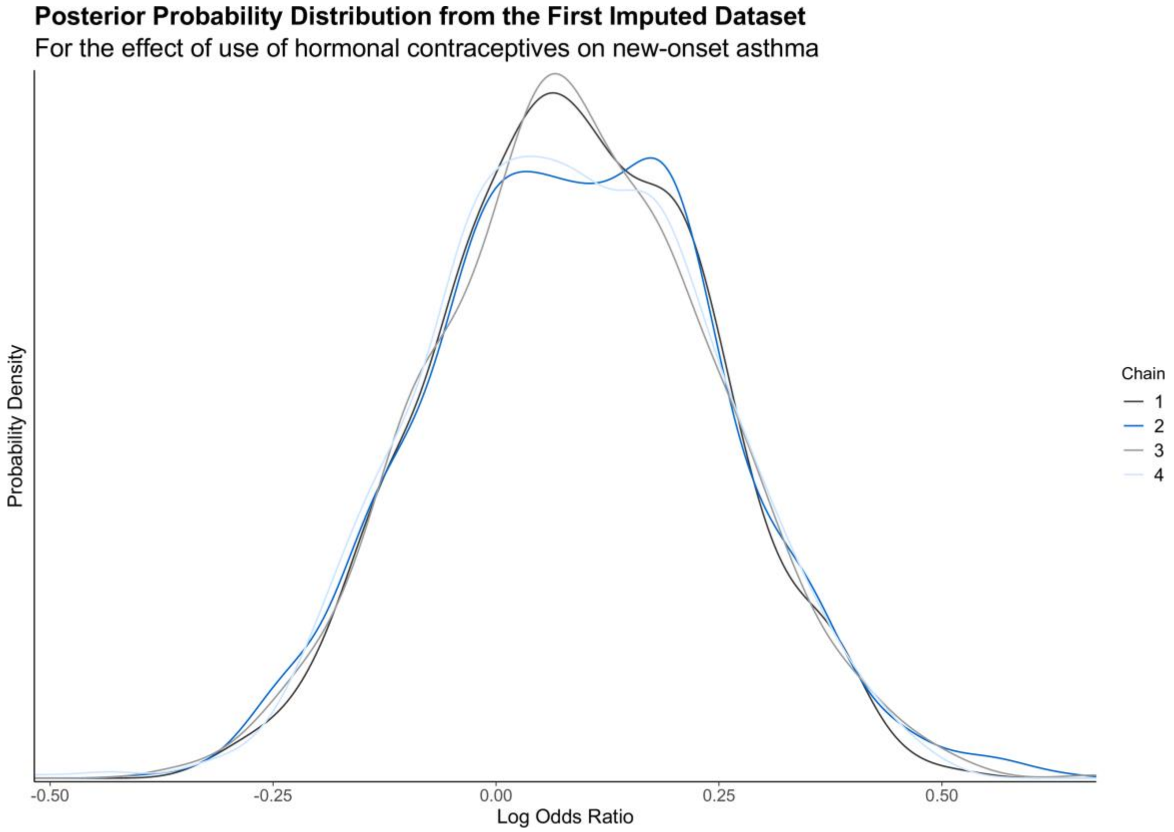

Figure S3. Posterior distributions estimated from each Markov chain in the first imputed dataset.

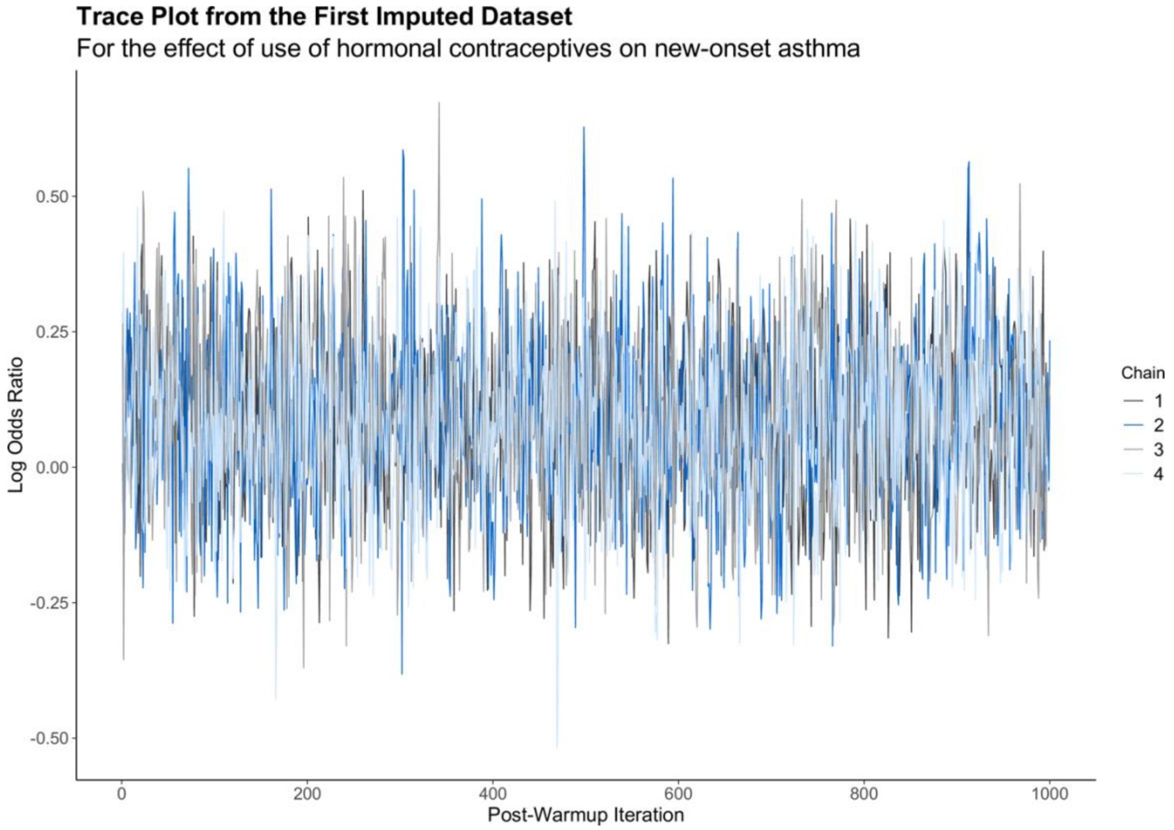

Figure S4. Trace plot from the first imputed dataset.

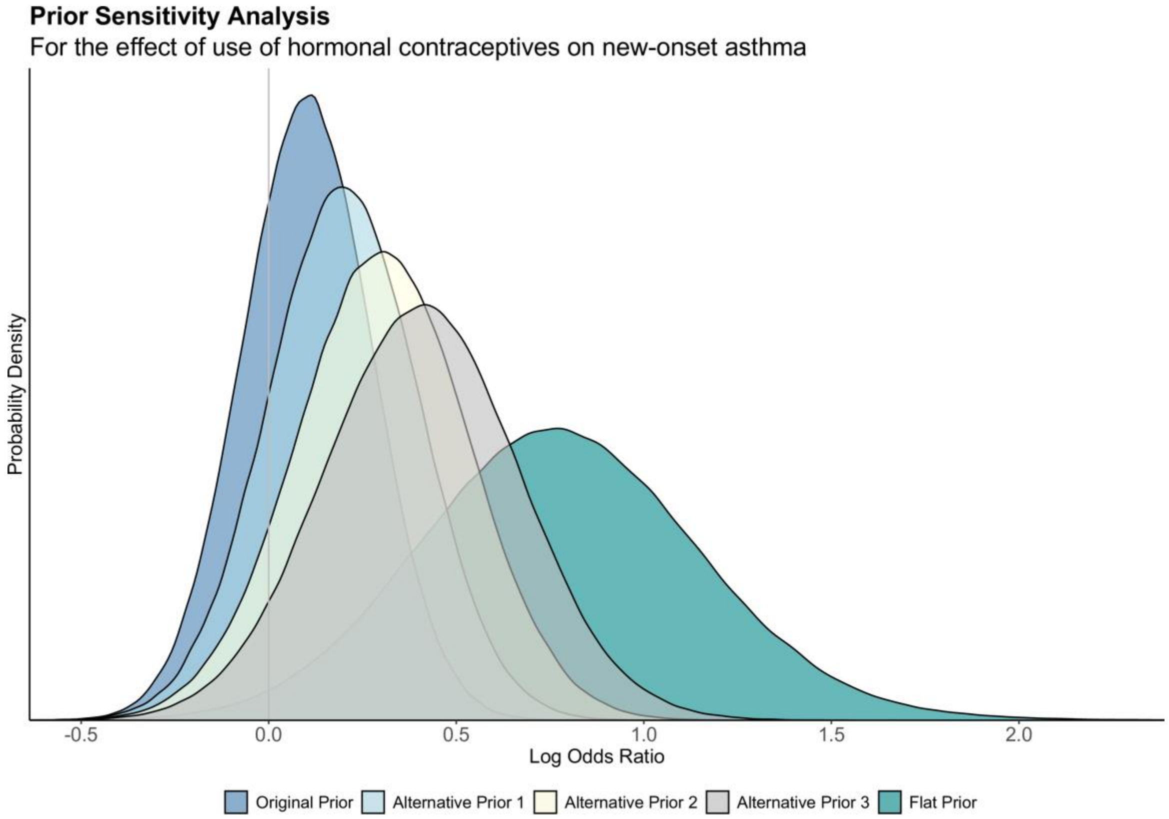

Figure S5. Posterior distributions for the original and alternative priors.
Original Prior = $N(-0.26, 0.20^2)$; Alternative Prior 1 = $N(-0.26, 0.25^2)$; Alternative Prior 2 = $N(-0.26, 0.30^2)$; Alternative Prior 3 = $N(-0.26, 0.38^2)$.

Menopausal Hormone Therapy and New-Onset Asthma in Menopausal Women

1. Prior Probability Distribution

Our recent umbrella review[6] including five prospective cohort studies with 163,161 women found that use of menopausal hormone therapy (MHT) compared to non-use was associated on average with a 1.41-fold (95% confidence interval 1.09–1.81) increased risk of new-onset asthma. Similarly, a nested case-control study based on the Danish registers,[7] including 34,533 women with asthma and 345,116 without asthma aged 40–65 years, reported that use of MHT was associated with an increased risk of new-onset asthma, with HRs ranging from 1.18 to 1.63. In contrast, a UK national retrospective cohort study,[8] including 353,173 women aged 46–70 years, found that use of MHT was associated with a decreased risk of new-onset asthma, with HRs ranging from 0.78 to 0.89.

Given that most of the epidemiologic studies reported an increased risk of developing asthma with use of MHT, we chose our *original* prior distribution as follows: we set that the *true average effect* of ever use of MHT compared to never use on asthma risk in our study population has an 80% probability of lying between 1.00 and 1.40 on OR scale, such that the original prior normal distribution on log OR scale has a mean of $(\log(1.00) + \log(1.40))/2 \approx 0.17$ and a SD of $(\log(1.40) - \log(1.00))/(1.28 \times 2) \approx 0.13$.

In addition, we specified *a priori* a set of *alternative* prior distributions, on log OR scale, of the same mean value (≈ 0.17), but a different SD ($\approx 0.16, 0.20\ or\ 0.25$), which respectively corresponds to a normal distribution with a 70%, 60% or 50% probability that the true average effect of use of MHT on asthma risk in our study population lies between 1.00 and 1.40 on OR scale. We also included a flat prior distribution for all parameters in the model. The original and alternative prior distributions are presented in Figure S6.

2. Posterior Probability Distribution

The posterior distribution based on the original prior is presented in **Figure 3B**. The median of the posterior distribution was 1.18 on OR scale, with 95% PI 0.92–1.52. The probability of OR being greater than 1 was 90.6%. Figure S7 presents the posterior distributions approximated from each multiply imputed dataset.

3. Sampling Quality Check

For the first imputed dataset, the posterior distribution approximated from each Markov chain is presented in Figure S8. For all parameters in the model, the $\hat{R}$ statistic was less than 1.1, and the ESS was more than 1,000. The trace plot is shown in Figure S9.

4. Prior Sensitivity Analysis

Figure S10 illustrates and compares the posterior distributions across models with different prior specifications. Table S2 summarizes the posterior estimates of each posterior distribution.

Table S2. Posterior estimates for the original and alternative priors for use of menopausal hormone therapy and new-onset asthma in menopausal women

Prior	Mean[a]	SD[a]	2.5%[b]	50%[b]	97.5%[b]	$P(>0)$
Original prior	0.17	0.13	0.92	1.18	1.52	90.6%
Alternative prior 1	0.17	0.15	0.87	1.18	1.60	86.2%
Alternative prior 2	0.17	0.19	0.82	1.18	1.70	81.6%
Alternative prior 3	0.17	0.22	0.76	1.18	1.82	77.2%
Flat prior[c]	0.14	0.47	0.45	1.17	2.81	62.8%

Mean = mean of posterior distribution; SD = standard deviation of posterior distribution; 2.5%–97.5% = 95% posterior central interval; 50% = median of posterior distribution; $P\ (>0)$ = probability that the regression coefficient for use of menopausal hormone therapy was greater than 0 (i.e., odds ratio >1). All the analyses were conducted based on the multiply imputed datasets, adjusted for age, place of residence, level of education, body mass index, tobacco smoking, environmental tobacco smoke, age at menopause, physical exercise, and gynecological conditions.

[a] On log odds ratio scale.

[b] On odds ratio scale.

[c] Flat priors for all parameters in the model.

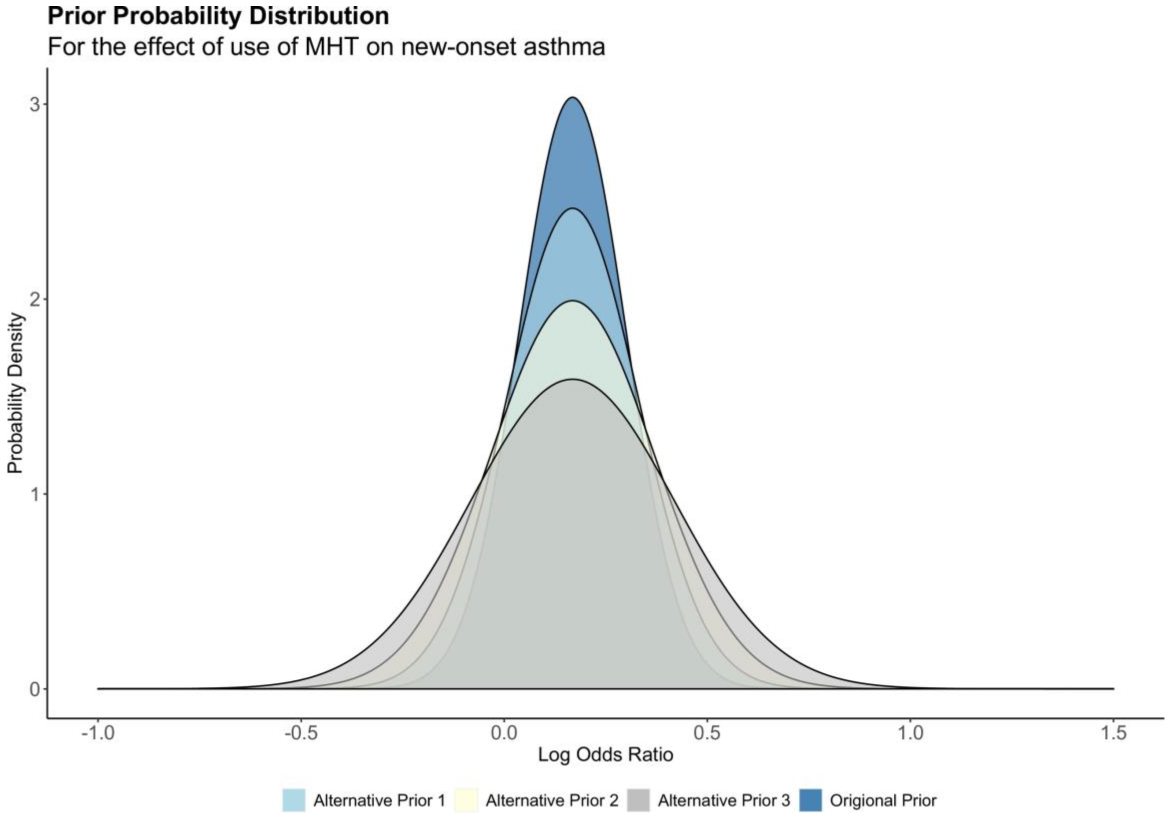

Figure S6. The original and alternative prior distributions.
Original Prior = $N(0.17, 0.13^2)$; Alternative Prior 1 = $N(0.17, 0.16^2)$; Alternative Prior 2 = $N(0.17, 0.20^2)$; Alternative Prior 3 = $N(0.17, 0.25^2)$. MHT, menopausal hormone therapy.

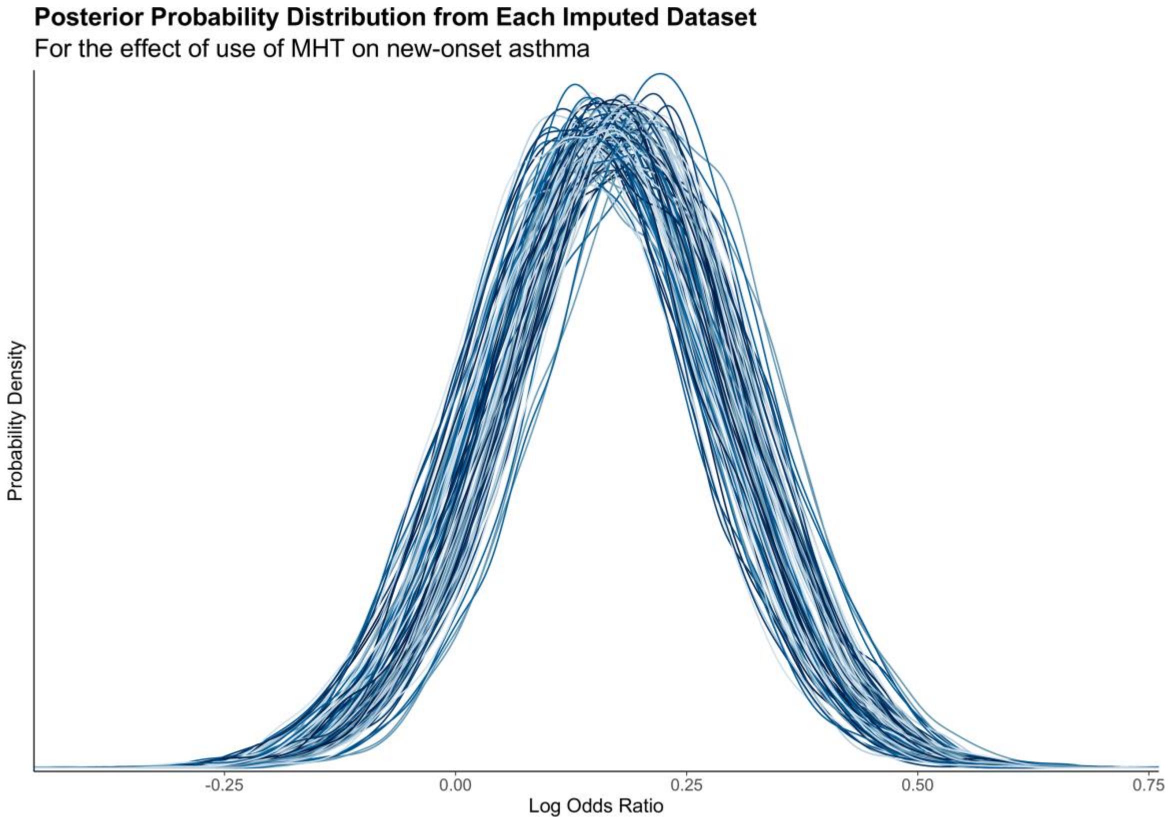

Figure S7. Posterior distributions estimated from each multiply imputed dataset (m = 100).
MHT, menopausal hormone therapy.

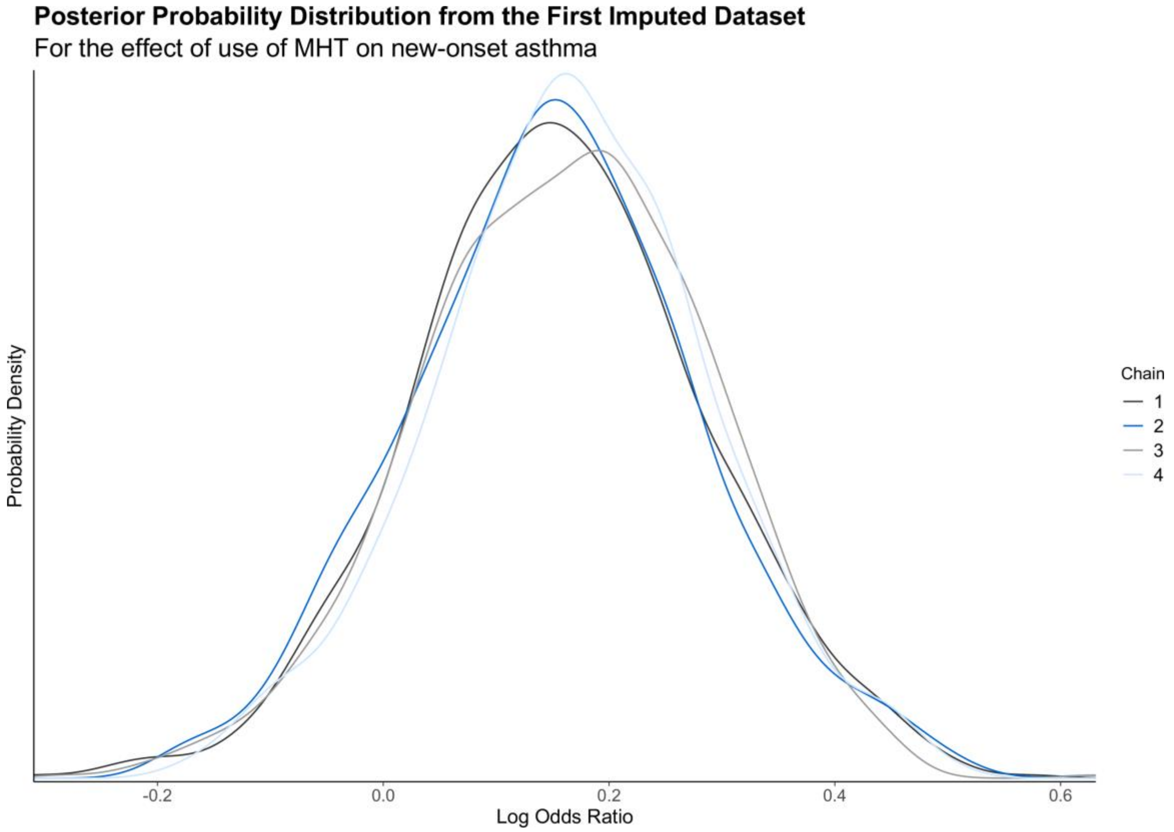

Figure S8. Posterior distributions estimated from each Markov chain in the first imputed dataset.
MHT, menopausal hormone therapy.

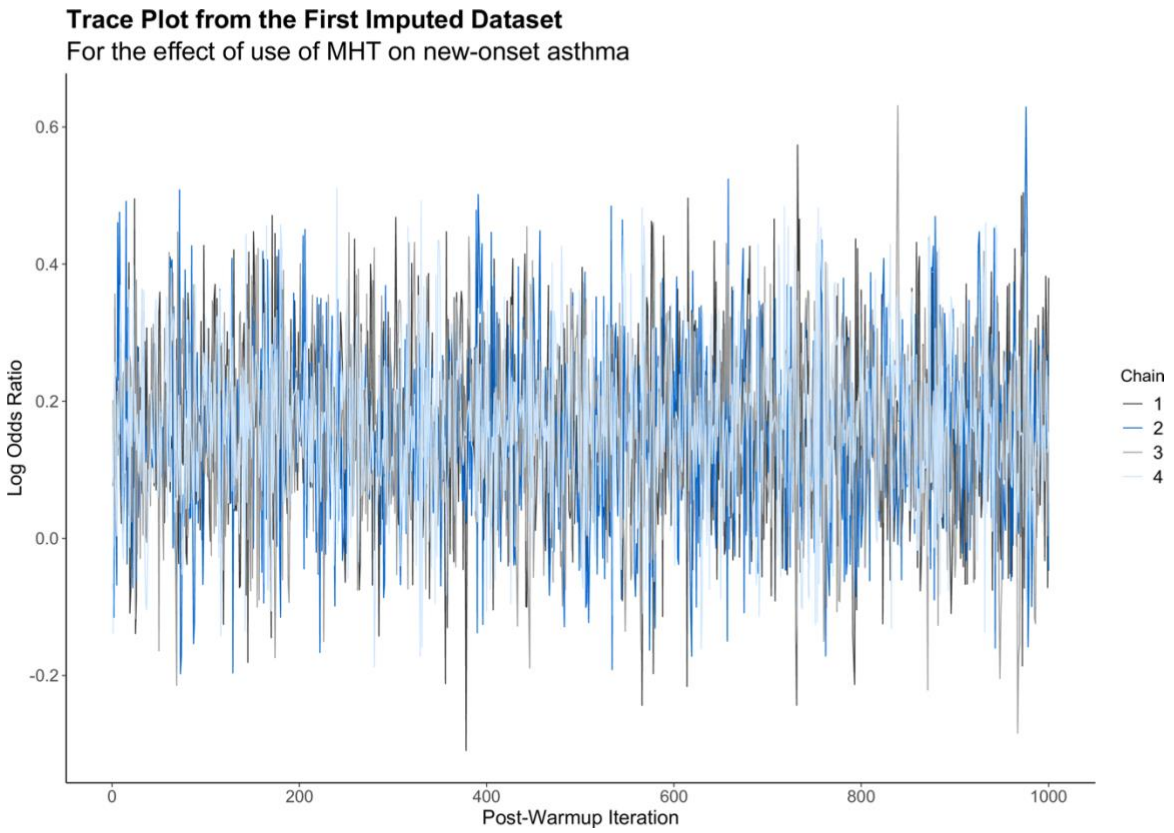

Figure S9. Trace plot from the first imputed dataset.
MHT, menopausal hormone therapy.

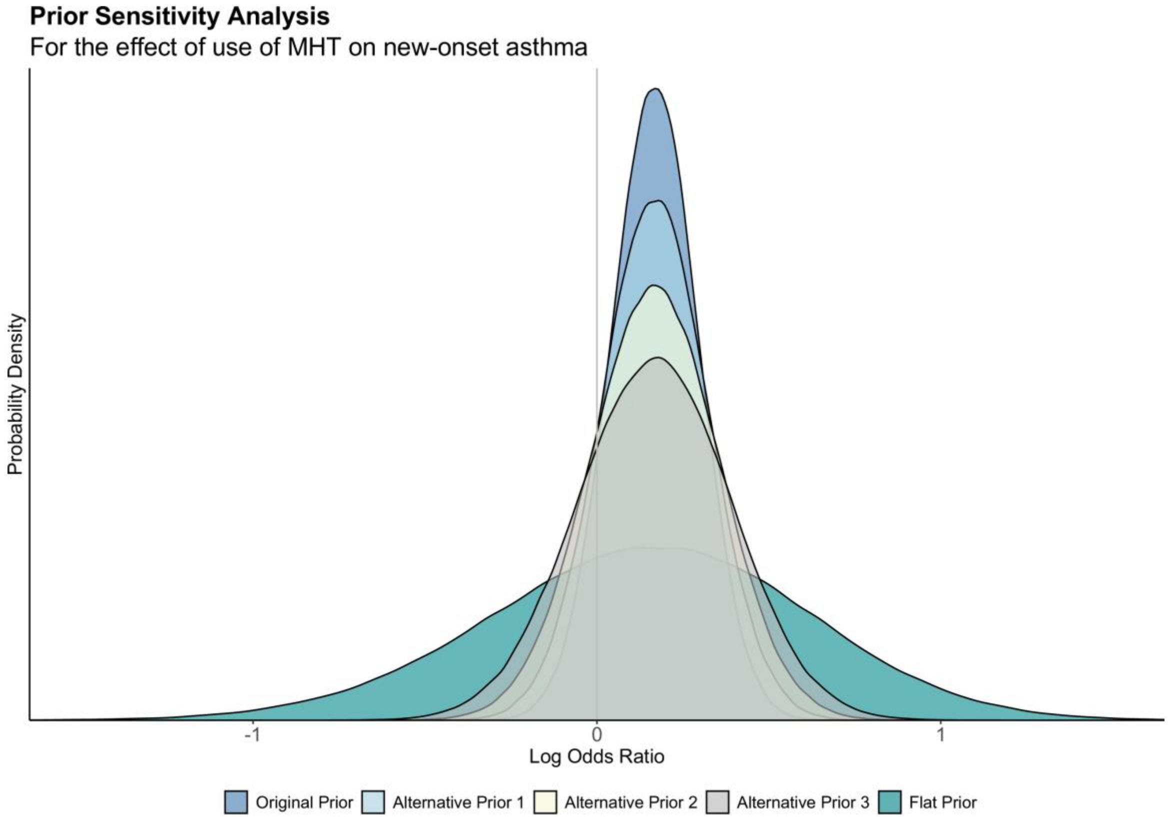

Figure S10. Posterior distributions for the original and alternative priors.
Original Prior = $N(0.17, 0.13^2)$; Alternative Prior 1 = $N(0.17, 0.16^2)$; Alternative Prior 2 = $N(0.17, 0.20^2)$; Alternative Prior 3 = $N(0.17, 0.25^2)$. MHT, menopausal hormone therapy.

eReferences

1. Zhang GQ, Özuygur Ermis SS, Rådinger M, Bossios A, Kankaanranta H, Nwaru B. Sex disparities in asthma development and clinical outcomes: implications for treatment strategies. J Asthma Allergy. 2022;15:231-47.
2. Wei J, Gerlich J, Genuneit J, Nowak D, Vogelberg C, von Mutius E, et al. Hormonal factors and incident asthma and allergic rhinitis during puberty in girls. Ann Allergy Asthma Immunol. 2015;115(1):21-7.
3. Nwaru BI, Pillinger R, Tibble H, Shah SA, Ryan D, Critchley H, et al. Hormonal contraceptives and onset of asthma in reproductive-age women: population-based cohort study. J Allergy Clin Immunol. 2020;146(2):438-46.
4. Troisi RJ, Speizer FE, Willett WC, Trichopoulos D, Rosner B. Menopause, postmenopausal estrogen preparations, and the risk of adult-onset asthma. A prospective cohort study. Am J Respir Crit Care Med. 1995;152(4 Pt 1):1183-8.
5. VanderWeele TJ, Ding P. Sensitivity analysis in observational research: introducing the E-value. Ann Intern Med. 2017;167(4):268-74.
6. Zhang GQ, Chen JL, Luo Y, Mathur MB, Anagnostis P, Nurmatov U, et al. Menopausal hormone therapy and women's health: an umbrella review. PLoS Med. 2021;18(8):e1003731.
7. Hansen ESH, Aasbjerg K, Moeller AL, Gade EJ, Torp-Pedersen C, Backer V. Hormone replacement therapy and development of new asthma. Chest. 2021;160(1):45-52.
8. Shah SA, Tibble H, Pillinger R, McLean S, Ryan D, Critchley H, et al. Hormone replacement therapy and asthma onset in menopausal women: national cohort study. J Allergy Clin Immunol. 2021;147(5):1662-70.
