# Supplementary material for: Exogenous female sex steroid hormones and new-onset asthma in women: a matched case–control study
Source: BMC Med. 2023 Sep 4;21:337. doi: 10.1186/s12916-023-03038-8 (PMC10478448; doi:10.1186/s12916-023-03038-8)
Supplement: Supplementary file 7 — Additional file 7: Figure S1. Previous cohort studies on use of hormonal contraceptives and new-onset asthma in women. Figure S2. Simplified causal directed acyclic graphs to represent the role of body mass index for the effect of use of hormonal contraceptives on new-onset asthma in women. [file 12916_2023_3038_MOESM7_ESM.pdf]

Additional File 7
Additional Information

The Additional file 7 contains additional information for:

Guo-Qiang Zhang*, Rani Basna, Maya B. Mathur, Cecilia Lässer, Roxana Mincheva, Linda Ekerljung, Göran Wennergren, Madeleine Rådinger, Bo Lundbäck, Hannu Kankaanranta, Bright I. Nwaru. Exogenous female sex steroid hormones and new-onset asthma in women: a matched case-control study. 2023.

* Corresponding author
Krefting Research Centre, Department of Internal Medicine and Clinical Nutrition, Institute of Medicine, Sahlgrenska Academy, University of Gothenburg, Gothenburg, Sweden
E-mail: guo-qiang.zhang@gu.se

Study	Age at Baseline	No. of Population	Type of Estimate	Relative Risk (95% CI)
**Wei et al 2015**[1]				
Ever use	9-11 years	1,191	OR	0.27 (0.12, 0.58)
**Nwaru et al 2020**[2]				
Current use	16-45 years	564,896	HR	0.63 (0.61, 0.65)
Past use	16-45 years	564,896	HR	0.70 (0.68, 0.72)
**Troisi et al 1995**[3,a]				
All women	34-68 years	64,237	HR	1.25 (1.07, 1.47)
Menopausal women	NA	NA	HR	1.57 (1.16, 2.12)

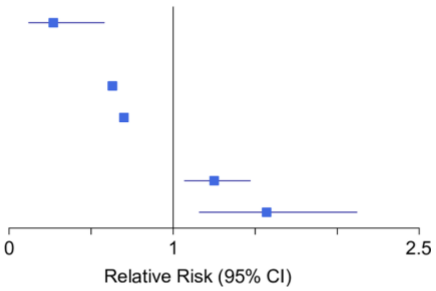

Figure S1. Previous cohort studies on use of hormonal contraceptives and new-onset asthma in women.

In a German prospective community-based cohort study[1] of 1,191 girls aged 9–11 years followed into early adulthood (19–24 years old), ever use of hormonal contraceptives was associated with decreased odds of new-onset asthma (odds ratio [OR] 0.27, 95% confidence interval [CI] 0.12–0.58); in a national UK cohort study[2] of 564,896 women aged 16–45 years, past or current use of hormonal contraceptives was associated with a decreased risk of new-onset asthma, with hazard ratios (HRs) ranging from 0.59 to 0.70 for different types of hormonal contraceptives; the Nurses' Health Study[3] including 64,237 women aged 34–68 years found that past use of hormonal contraceptives was associated with an increased risk of new-onset asthma (HR 1.25, 95% CI 1.07–1.47), and the effect estimate became even larger among naturally menopausal women (HR 1.57, 95% CI 1.16–2.12).

Abbreviations: CI, confidence interval; HR, hazard ratio; NA, not available; OR, odds ratio.

[a] Past use of hormonal contraceptives.

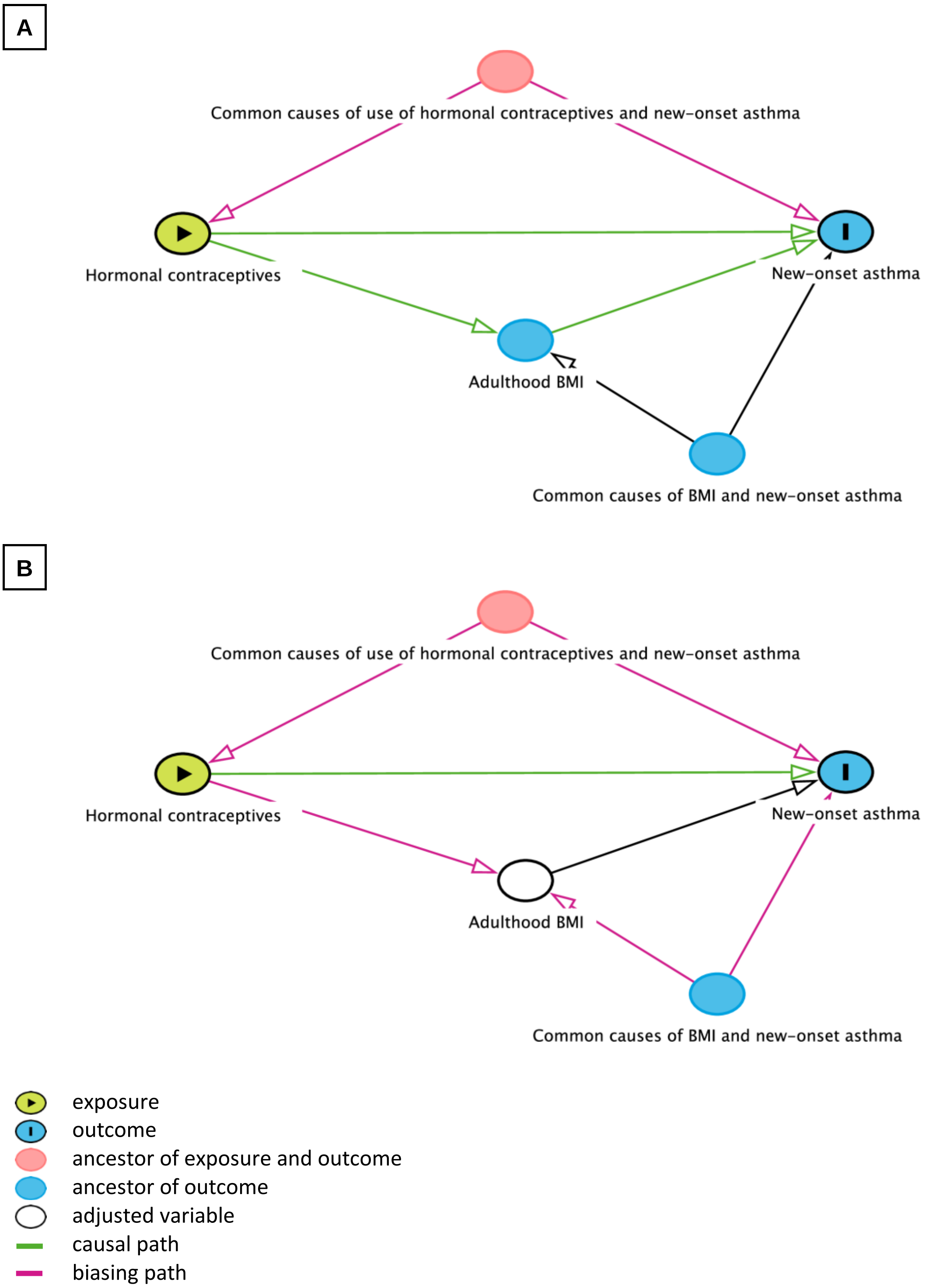

Figure S2. Simplified causal directed acyclic graphs to represent the role of body mass index for the effect of use of hormonal contraceptives on new-onset asthma in women.
A. When one is interested in the overall causal effect of use of hormonal contraceptives on new-onset asthma, adjustment for body mass index (BMI) is unwarranted. B. When one is interested in the modifying effect of BMI, stratification by BMI is justified, but would introduce a spurious association between hormonal contraceptives and new-onset asthma, in the presence of common causes of BMI and new-onset asthma (e.g., physical activity, alcohol, stress, diet). Abbreviations: BMI, body mass index. The directed acyclic graphs were drawn using DAGitty (http://dagitty.net).

eReferences

1. Wei J, Gerlich J, Genuneit J, Nowak D, Vogelberg C, von Mutius E, et al. Hormonal factors and incident asthma and allergic rhinitis during puberty in girls. Ann Allergy Asthma Immunol. 2015;115(1):21-7.
2. Nwaru BI, Pillinger R, Tibble H, Shah SA, Ryan D, Critchley H, et al. Hormonal contraceptives and onset of asthma in reproductive-age women: population-based cohort study. J Allergy Clin Immunol. 2020;146(2):438-46.
3. Troisi RJ, Speizer FE, Willett WC, Trichopoulos D, Rosner B. Menopause, postmenopausal estrogen preparations, and the risk of adult-onset asthma. A prospective cohort study. Am J Respir Crit Care Med. 1995;152(4 Pt 1):1183-8.
