# Supplementary material for: Exogenous female sex steroid hormones and new-onset asthma in women: a matched case–control study
Source: BMC Med. 2023 Sep 4;21:337. doi: 10.1186/s12916-023-03038-8 (PMC10478448; doi:10.1186/s12916-023-03038-8)
Supplement: Supplementary file 2 — Additional file 2: Table S1. Potential common causes of use of hormonal contraceptives or menopausal hormone therapy and new-onset asthma in women. Figure S1. A causal directed acyclic graph for potential common causes of use of hormonal contraceptives and new-onset asthma in women. Figure S2. A causal directed acyclic graph for potential common causes of use of menopausal hormone therapy and new-onset asthma in menopausal women. Figure S3. A causal directed acyclic graph for potential selection bias of hormonal exposures and new-onset asthma in women. Figure S4. A causal directed acyclic graph for potential measurement bias of hormonal exposures and new-onset asthma in women. [file 12916_2023_3038_MOESM2_ESM.pdf]

Additional File 2
Statistical Analysis Protocol (August 23rd, 2022)

The Additional file 2 contains the statistical analysis protocol for:

Guo-Qiang Zhang*, Rani Basna, Maya B. Mathur, Cecilia Lässer, Roxana Mincheva, Linda Ekerljung, Göran Wennergren, Madeleine Rådinger, Bo Lundbäck, Hannu Kankaanranta, Bright I. Nwaru. Exogenous female sex steroid hormones and new-onset asthma in women: a matched case-control study. 2023.

* Corresponding author
Krefting Research Centre, Department of Internal Medicine and Clinical Nutrition, Institute of Medicine, Sahlgrenska Academy, University of Gothenburg, Gothenburg, Sweden
E-mail: guo-qiang.zhang@gu.se

[The supplementary Tables and Figures are attached at the end of the document, and hyperlinked within the text to allow for easy navigation between the text and the supplementary Tables and Figures.]

Table of Contents

1. RESEARCH QUESTIONS 3
2. DIRECTED ACYCLIC GRAPHS 3
3. INDIVIDUAL MATCHING (*R*-TO-1 MATCHING) 4
4. STATISTICAL ANALYSES 4
4.1. MULTIPLE IMPUTATION 4
4.2. FREQUENTIST CONDITIONAL LOGISTIC REGRESSION 5
4.3. BAYESIAN CONDITIONAL LOGISTIC REGRESSION 6
5. ADDITIONAL ANALYSES 8
5.1. SENSITIVITY ANALYSIS FOR RESIDUAL CONFOUNDING 8
5.2. EXPLORATORY SUBGROUP ANALYSES 8
6. SUPPLEMENTARY TABLES AND FIGURES 9
EREFERENCES 14

1. Research Questions

- Does ever use of hormonal contraceptives influence the risk of new-onset asthma in women?
- Does ever use of menopausal hormone therapy (MHT) influence the risk of new-onset asthma in menopausal women?

2. Directed Acyclic Graphs

In our study, the goal was to estimate the causal effect of use of hormonal contraceptives or MHT on the risk of developing new-onset asthma in women, using observational data from the West Sweden Asthma Study (WSAS) cohort.[1,2] In order to make valid causal inference from observational analysis, it is crucial that potential systematic biases, including confounding bias, selection bias and measurement bias, are identified and accounted for in the analysis.[3] Confounding bias is defined as the bias caused by common causes of the exposure and the outcome.[4] Selection bias refers to the bias resulting from conditioning on the common effect of two variables, one of which is either the exposure or associated with the exposure, and the other is either the outcome or associated with the outcome.[5,6] Confounding bias and selection bias can lead to lack of *exchangeability* (or comparability) between the exposed and the unexposed.[3] A number of investigators have emphasized that causal *directed acyclic graphs* (DAGs) can be used as a visual aid to help represent and classify systematic biases and further guide data analyses.[3,7–11] DAGs intuitively encode the investigators' qualitative subject-matter knowledge and *a priori* assumptions about the causal structure of interest, and are increasingly used in modern epidemiology.[3,8,9,12] Briefly, a causal DAG consists of nodes (variables) and directed edges (arrows). The presence of an arrow between two variables indicates that there is a causal effect of one variable on the other for at least one individual in the population. The absence of an arrow indicates that there is no causal effect of one variable on the other for any individual in the population. When a DAG is drawn, the *backdoor criterion* can be applied to determine *a sufficient set of adjustment variables* required to achieve (approximate) conditional exchangeability between the exposed and the unexposed.[3,4]

Therefore, for each research question in our study, we built causal DAGs to represent our *a priori* subject-matter knowledge and assumptions about the underlying causal structure. Table S1 presents detailed justifications for the common causes of the exposures of interest and new-onset asthma among women (i.e., confounding bias). In our study, there were potential sources of selection bias, such as non-response, loss of follow-up, and missing data (Figure 1).[5] Furthermore, the fact that the study population was restricted to women who had never had asthma at baseline (the year 2008) would likely introduce selection bias (if the hormonal exposures had a causal effect on new-onset asthma in women). This is because for some (or most) women, the hormonal exposures occurred before the study was initiated in the year 2008. For example, women aged 30 years at baseline might have started using hormonal contraceptives at 18 years old. Similarly, women aged 60 years at baseline might have started MHT at 50 years old. That is, *if* the hormonal exposures increased the risk of new-onset asthma, the more susceptible individuals would have developed asthma before baseline in the exposed group than in the unexposed group. As a result, restricting to individuals who had not had asthma at baseline would likely result in differential proportion of

susceptible individuals after baseline, thereby introducing selection bias.[5,13] To well illustrate different potential sources of bias in our study, we presented confounding bias (Figure S1 and Figure S2), selection bias (Figure S3) and measurement bias (Figure S4) in separate DAGs. The DAGs were drawn using DAGitty (http://dagitty.net).[14]

Notably, there is no guarantee that the proposed causal DAGs are exactly correct.[4,9] In other words, our attempts to identify a sufficient set of adjustment variables to achieve (approximate) conditional exchangeability would not necessarily be successful. However, the structural approach to systematic biases makes our assumptions about the causal network explicit and helps ensure consistency between our beliefs and analytic models.[3,4] Importantly, it enhances communication among investigators.

3. Individual Matching (*R*-to-1 Matching)

- The variables used for matching cases with controls were exact age in years at baseline in 2008, place of residence (in Gothenburg or outside Gothenburg), and smoking status (never smoker, past smoker, or current smoker).
- We used *category matching* to match cases with controls[15]: firstly, we categorized each of the three matching variables separately; secondly, for each case, we determined her/their age-residence-smoking combination and found all the controls with the same age-residence-smoking combination (each combination is a matched set or stratum); finally, we drew a random sample ($n = 10$) from the control population in each stratum; in strata where the number of controls in the population was equal to or less than 10, all the controls were selected.
- The potential advantage of matching in case-control studies is that it can lead to a balanced number of cases and controls across the levels of the matching variables, which *may* reduce the variance in estimating the parameters of interest and therefore improve *statistical efficiency*.[15,16]

4. Statistical Analyses

4.1. Multiple imputation

In our study population, some individuals have missing values for the exposure and/or adjustment variables. First, we will conduct *complete-case analysis*, that is, restricting the analysis to individuals with complete data on all variables included in the analytic model (see page 5 for more details). However, complete-case analysis will cause loss of information and may lead to biased estimates.[17] Second, we will apply *multiple imputation* (MI) to impute the missing values in the incomplete variables, which can account for the statistical uncertainty in the imputed values.[18,19] In brief, MI uses the distribution of the observed data to impute multiple versions of plausible values for the missing data, fits an identical analytic model to each imputed dataset, and finally combines the analytic results to obtain overall estimates (e.g., using Rubin's rules).[19–21]

We will use *full-conditional specification* (FCS) MI, also known as MI by chained equations (MICE).[22–24] FCS MI multiply imputes missing data from conditional distribution of each incomplete variable given other variables (including the matching variables in a matched case-control design).[22] In other words, the matching is broken to impute missing values of incomplete variables, and later

restored for data analysis (called “*MI using matching variables*”).[22] FCS MI can impute missing values for both continuous and categorical variables; and in a matched case-control design, it can allow for situations where there are multiple matched controls per case or the number of matched controls varies between cases.[22,23]

The imputation process in our study consists of the following seven main steps[23]:

- **Missing data assumption**[19]: We assume that data are missing at random (MAR), that is, “the probability of data being missing does not depend on the unobserved data, conditional on the observed data”.
- **Specify imputation model**[19]: We will use predictive mean matching (PMM) to impute continuous variables, logistic regression for binary variables, and multinomial logistic regression for categorical variables with ≥ 3 levels; the main virtues of PMM are that it requires neither linearity assumption between dependent and independent variables, nor normality assumption in dependent variables, and the imputed values are restricted to the observed ones.
- **Select predictors for imputation model**[19,24]: We will select all variables that are included in the final analytic model as predictors in the imputation model; particularly, the outcome variable must be included[25]; in addition, for incomplete variables in the analysis model, we will identify *auxiliary variables* (i.e., variables that either correlate with the incomplete variable or predict the missingness of the incomplete variable, but are not included in the analytic model[23,26]) and include them into the imputation model, so as to make the MAR assumption more plausible and thus reduce bias.
- **Impute transformed variables**[24]: For transformed variables (e.g., body mass index) derived from original (incomplete) variables (e.g., height and weight), if the original variables need to be included in the imputation model (as predictors for the transformed variables and/or other incomplete ones), we will use *passive imputation* to ensure that the imputed values of the original and transformed variables are consistent; otherwise, we will first impute the original variables and then transform the completed.
- **Number of iterations**[23]: Simulation studies suggest that for datasets with moderate amounts of missing data, five or 10 iterations would give satisfactory performance; therefore, we will use 10 iterations for imputations.
- **Number of imputations**[19]: The rule of thumb is that the number of imputations should be more than or equal to the percentage of incomplete cases in the variable being imputed, though this may not be universally appropriate; we will impute 100 datasets, each of which will comprise the observed data and the imputed values for the missing data.
- **Validate imputations**[23]: We will check the convergence of the imputations, visualize the distributions of imputed data, and compare the distributions of observed data with those of imputed data.

4.2. Frequentist conditional logistic regression

- As the matching criteria are relatively fine (cases and controls were exactly matched on three variables), when adjusting for the matching variables, there would be a number of dummy variables in the model; in this case where the

number of parameters is *large* relative to the number of participants, we will use conditional logistic regression.[15,27–29]

- In a matched case-control design, although matching ensures that cases and controls have similar distributions across the matching variables, matching in itself does not eliminate confounding by the matching variables, but in fact can *introduce* selection bias[27,30]; in order to remove this bias as well as to control for confounding by the matching variables, the matching variables need to be adjusted for in the analysis[16,27]; therefore, we will adjust for the matching variables in the analysis.
- We will identify the matching strata that have identical values for the matching variables (i.e., *exchangeable* matched sets), and combine these strata into a single stratum before analysis; the advantage of this approach is that fewer data may be discarded during analysis (e.g., because strata where the case and the control have the same exposure status will be discarded in conditional logistic regression) and statistical precision may be improved.[15,27,31]
- In order to achieve (approximate) conditional exchangeability between the exposed and the unexposed, we will adjust for the sufficient set of confounding variables identified using causal DAGs; for each research question, Figure S1 and Figure S2 present the full list of variables that will need to be adjusted for in the regression model; for continuous confounding variables, we will include them as linear terms (instead of as categories) in the regression model.[32,33]
- We will apply the Frequentist conditional logistic regression model both in the original incomplete dataset and in the multiply imputed datasets.
- It is worth noting that logistic regression can estimate only the *conditional* causal odds ratio, which is different from the *marginal* causal odds ratio in the population.[16,34]

4.3. Bayesian conditional logistic regression

The Bayesian statistical framework applies formal probability models to describe our uncertainty about the unknown parameters of interest.[35] It can naturally accommodate our *prior* beliefs (or uncertainty) about the parameters before observing the data, and update these beliefs about the parameters after observing the data (i.e., our *posterior* beliefs).[35] The relationship between our prior and posterior beliefs about the parameters can be presented by Bayes' theorem[36]:

$$P(parameters|data) = \frac{P(parameters) \times P(data|parameters)}{P(data)}$$

Or more generally,

$$posterior \propto prior \times likelihood$$

where $\propto$ means "is proportional to". That is, the Bayesian framework computes the *posterior probability distributions* over all possible values of the parameters, conditional on the *prior probability distributions*, statistical model and observed data.[37] In contrast, the Frequentist statistical framework is concerned with the probability of the *observed data* given the unknown parameters.[37] Thus, the

Bayesian framework allows us to easily make intuitive probabilistic statements about the parameters of interest. For example, we can apply a Bayesian logistic regression model to answer questions like "*What is the probability that odds ratio is larger than 1?*". In recent years, the Bayesian statistical methods have become more popular in epidemiologic research.[38]

The Bayesian modelling in our study consists of the following five main steps[37]:

- **Specify data model**: Given the study design, we will use conditional logistic regression model (see page 5 for justification).
- **Specify prior probability distribution**: The prior probability distribution represents our prior beliefs about the parameters of interest before seeing the data, which specifies the possible values of the parameters and the relative plausibility of each value; we will use normal distributions for the prior distributions; for each hormonal exposure, we plan to estimate a predictive distribution from previous meta-analyses as the prior distribution[39]; however, it is recommended that at least 10 studies are needed for reasonable estimation of predictive distribution[40,41]; if there are less than 10 studies, we will refer to our previous review work[42–44] as well as newly published studies, while taking into account potential systematic biases in existing studies,[36,39] to inform the priors, known as *informative prior distributions*; then we will conduct prior sensitivity analysis[45] to examine the robustness of results under different prior specifications, and the results will be compared through a series of visual and statistical comparisons (see Additional file 4 for more details); the point is to use different possible priors to represent our uncertainty around the prior; for adjustment variables, we will use the default priors (*weakly informative priors*) in R package 'rstanarm' (version 2.21.1)[46]; weakly informative priors can provide moderate regularization and help stabilize computation, while still allowing for extreme values when warranted by the data.[37,46,47]
- **Approximate posterior probability distribution**: The posterior probability distribution describes the relative plausibility of all possible parameter values conditional on the priors, model, and data; we will use a Markov Chain Monte Carlo (MCMC) method – the Hamiltonian Monte Carlo (HMC) algorithm – to draw samples from the posterior distribution of the parameter, to therefore approximate the posterior distribution[46]; specifically, we will fit a Bayesian conditional logistic regression model in each multiply imputed dataset (not in the original incomplete dataset), simulate many MCMC draws from their respective posterior distributions (we will run four randomly initialized Markov chains, each for 2,000 iterations, including a warmup period of 1,000 iterations that will be discarded), and finally mix all the MCMC draws together to approximate the posterior distribution[48]; this approach has been suggested to work well with a large number of imputed datasets (e.g., 100)[48]; we will also compare the approximated posterior distributions from each multiply imputed dataset.
- **Markov chain diagnostics**: To check the MCMC sampling quality, we will examine the trace plot, calculate two supplementary diagnostic metrics ($\hat{R}$ statistic and effective sample size [ESS]), and compare posterior distributions approximated from each MCMC chain[37]; in general, it is recommended that $\hat{R} <$

1.1 and ESS $\geq$ 1,000 for all parameters in the model are required to ensure healthy convergence and enough precision[37,49]; for simplicity, we will check only for the first imputed dataset.

- **Summarize and interpret results**: We will use the combined MCMC draws to approximate the posterior probability distribution[48]; we will calculate the *median* as the point estimate, and the equal-tailed interval as the *posterior interval* (PI) (also known as *credible interval*)[50]; for example, a 95% PI includes the 95% *central* portion of the posterior distribution (excluding 2.5% from each tail of the distribution); thus, a 95% PI means that "Given the priors, statistical model, and observed data, we are 95% certain that the parameter lies within this interval"; we will also estimate the probability that the regression coefficient for the hormonal exposure (on log odds ratio scale) is greater or less than 0.

5. Additional Analyses

5.1. Sensitivity analysis for residual confounding

To assess the robustness to (potential) residual confounding of the estimated causal effects of hormonal exposures on the risk of new-onset asthma in women using the WSAS observational data,[2] we will calculate E-value,[51] which indicates how strong residual confounding would have to be to "*explain away*" an observed causal effect. Specifically, E-value represents the minimum strength of association, on the risk ratio (RR) scale, that residual confounding would need to have with both the exposure and the outcome, conditional on the measured variables, to shift the effect estimate to a chosen threshold of scientific importance. In our study, the threshold of RR = 1.0 will be used. A large E-value would indicate that the effect estimate is relatively robust to residual confounding, whereas a small E-value would indicate that the effect estimate is relatively sensitive to residual confounding. *Notes: As noted in VanderWeele et al 2017,*[51] *E-value is a continuous measure, and use of any threshold cutoff is discouraged; a small E-value only implies that the evidence for an effect is weak, but does not mean that there is evidence for no effect.*

5.2. Exploratory subgroup analyses

As explained on page 3 and also illustrated in Figure S3, because women with ever asthma at baseline were excluded in our study design, it would result in selection bias, *only* if hormonal exposures have a causal effect on the risk of developing new-onset asthma in women. That is, selection bias would be anticipated, especially among women of older age groups at baseline, if there exists a causal effect of hormonal exposures on asthma. Specifically, we expect that with age at baseline becoming older, the effect estimate would gradually be biased towards the opposite direction of the true effect. To explore whether there exists this pattern, we will conduct subgroup analyses by age at baseline in 2008 under the Frequentist framework. The age cut-offs used for subgroup analyses will be based on the age distribution in our study sample.

6. Supplementary Tables and Figures

Table S1. Potential common causes of use of hormonal contraceptives or menopausal hormone therapy and new-onset asthma in women

Potential causes for new-onset asthma[a]	Use of HCs[b]	Use of MHT[c]
**Demographic factors**		
Age[52–57]	🔴	🔴
Socioeconomic status[54,55,57–61]	🔴	🔴
Body mass index[54,55,62–65]	🟢	🔴
**Lifestyle factors**		
Diet (e.g., fruit/vegetables, fast food)[66]		🔴
Physical activity[67–69]		🔴
Tobacco smoking[55,65,70]		🔴
Alcohol[55,70]		🔴
**Environmental factors**		
Environmental tobacco smoke[55,65]		🔴
**Hormonal factors**		
Menarche (age at menarche)	🔴	
Gravidity	🟢	
Menopause (age at menopause)[54,59,60]		🔴
**Other factors**		
Gynecological conditions[d,52,54,56,58,59]	🔴	🔴

Abbreviations: HCs, hormonal contraceptives; MHT, menopausal hormone therapy.
[a] The list of factors was based on literature reviews on potential risk factors for new-onset asthma in adults[42,71–73]; factors that were considered not to be causally related to either of the exposures of interest were excluded.
[b] Among all women.
[c] Among menopausal women.
[d] Including endometriosis, polycystic ovarian syndrome, gynecological acne, and hysterectomy with or without oophorectomy.

🔴 The factor is likely to cause the exposure.
🟢 The exposure is likely to cause the factor.

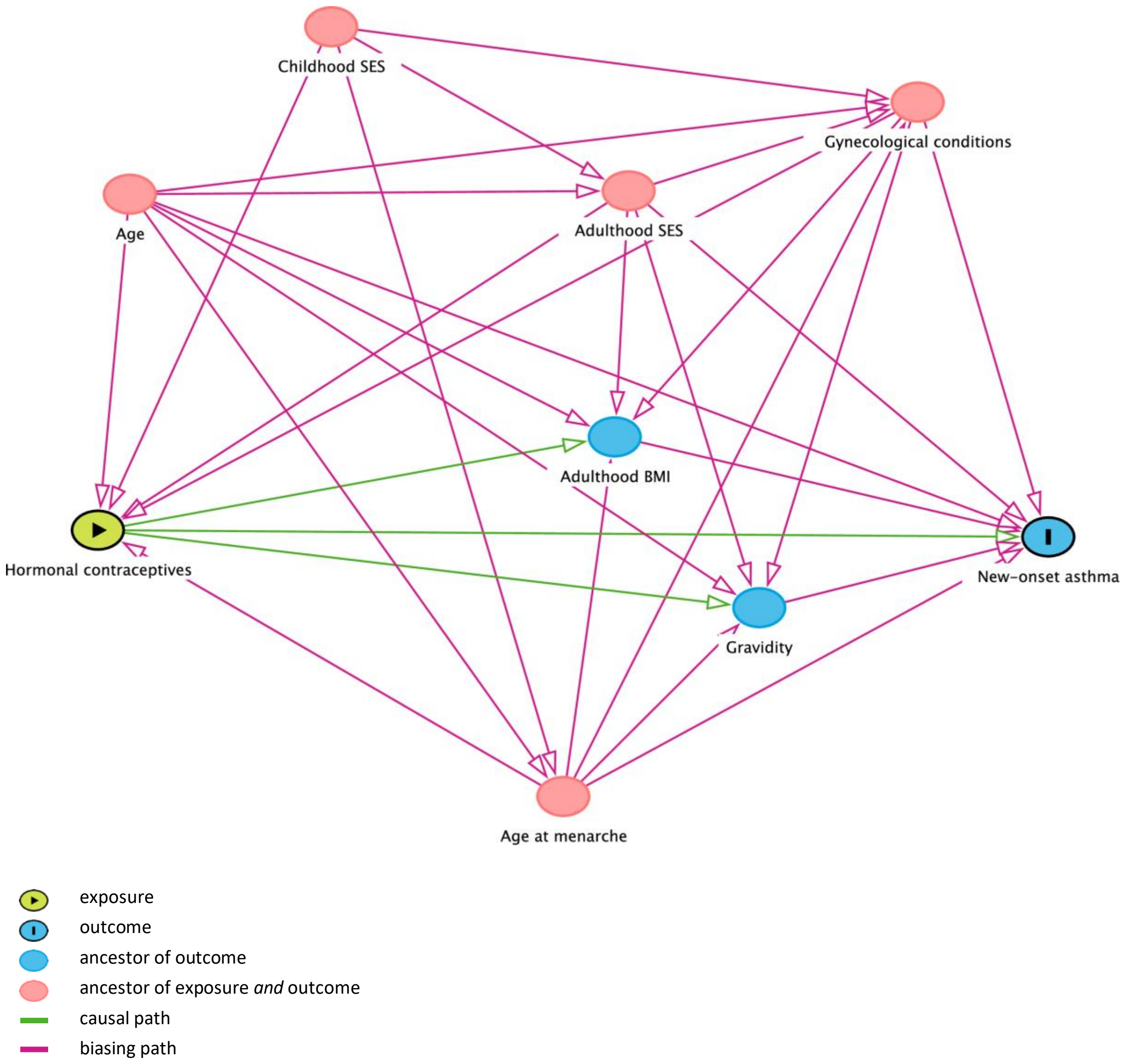

Figure S1. A causal directed acyclic graph for potential common causes of use of hormonal contraceptives and new-onset asthma in women.
Abbreviations: BMI, body mass index; SES, socioeconomic status.
The sufficient set of adjustment variables to eliminate confounding bias for use of hormonal contraceptives and new-onset asthma in women includes age, adulthood socioeconomic status, age at menarche, and gynecological conditions.

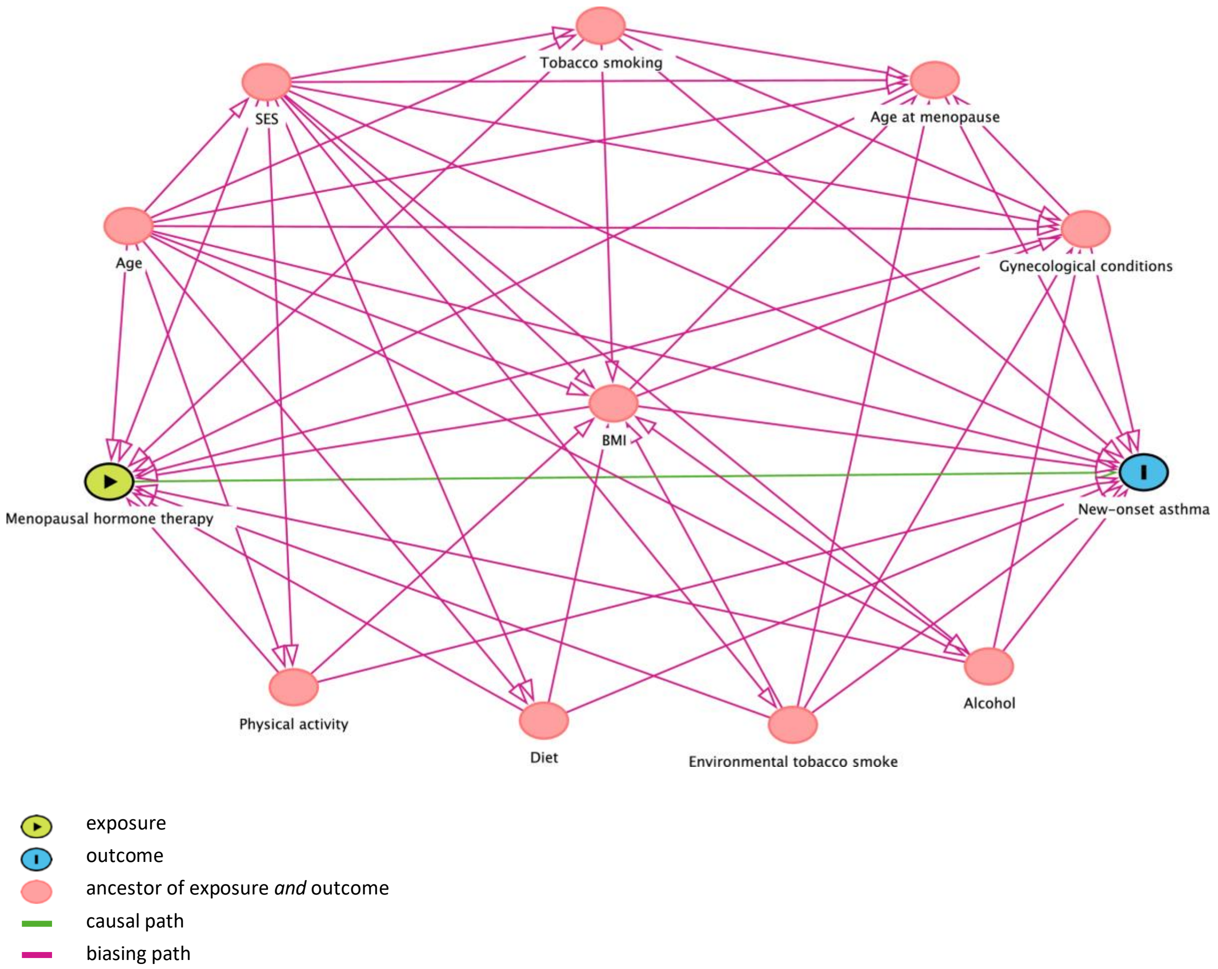

Figure S2. A causal directed acyclic graph for potential common causes of use of menopausal hormone therapy and new-onset asthma in menopausal women.

Abbreviations: BMI, body mass index; SES, socioeconomic status.

The sufficient set of adjustment variables to eliminate confounding bias for use of menopausal hormone therapy and new-onset asthma in menopausal women includes age, body mass index, socioeconomic status, age at menopause, physical activity, tobacco smoking, alcohol, diet, environmental tobacco smoke, and gynecological conditions.

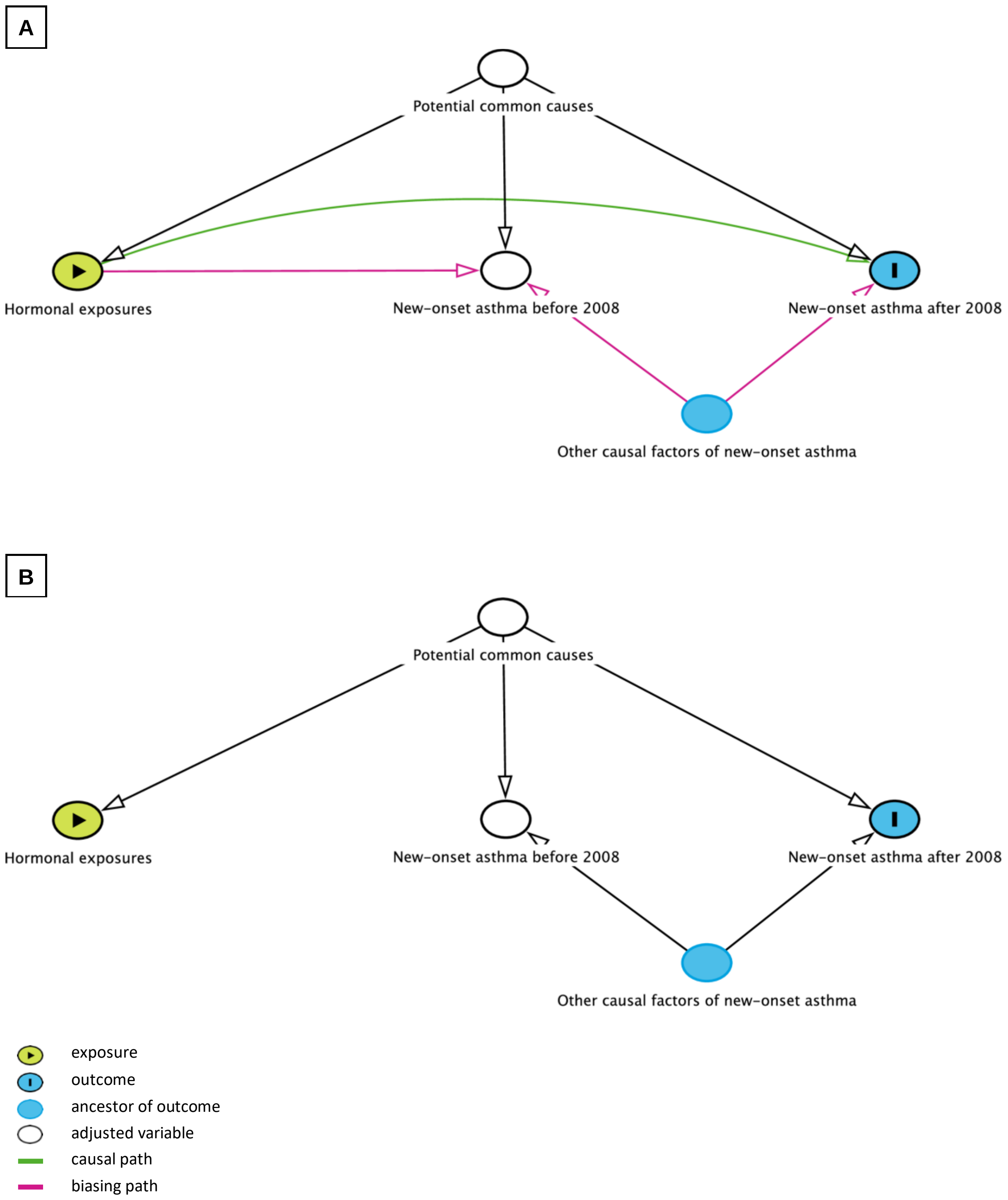

Figure S3. A causal directed acyclic graph for potential selection bias of hormonal exposures and new-onset asthma in women.

Hormonal exposures include ever use of hormonal contraceptives or menopausal hormone therapy. For simplicity, we assumed that the potential common causes of the exposures and the outcome were fully measured and could be controlled for during analysis. A. If the exposures *have* a causal effect on new-onset asthma in women, conditioning on asthma status at baseline in 2008 (i.e., a collider) would introduce selection bias. B. If the exposures do *not* have a causal effect on new-onset asthma in women, conditioning on asthma status in 2008 would *not* introduce selection bias.

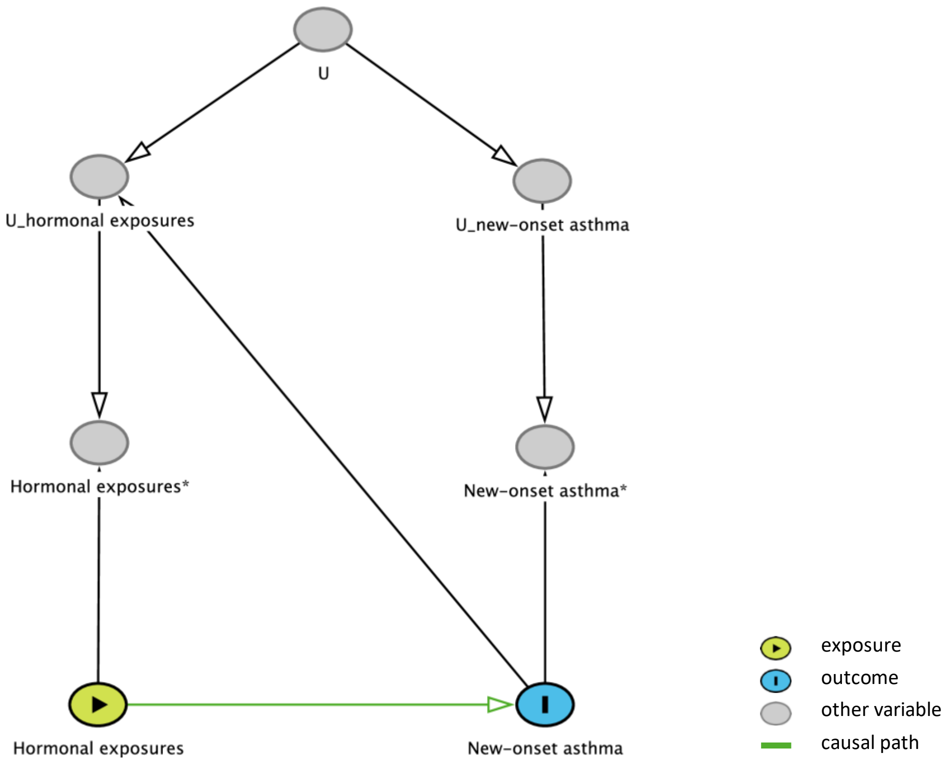

Figure S4. A causal directed acyclic graph for potential measurement bias of hormonal exposures and new-onset asthma in women.

Hormonal exposures include ever use of hormonal contraceptives or menopausal hormone therapy. *U_hormonal exposures*: the measurement error for hormonal exposures; *Hormonal exposures**: the measured hormonal exposures; *U_new-onset asthma*: the measurement error for new-onset asthma; *New-onset asthma**: the measured new-onset asthma. In our study, information on the hormonal exposures and the outcome was obtained retrospectively by questionnaire survey; thus, an individual's ability to recall their medical history (U) may affect the measurement of both hormonal exposures and outcome. In addition, the hormonal exposures were ascertained by recall after the outcome had occurred; thus, the outcome *might* affect the recall of the hormonal exposures (i.e., an arrow from *New-onset asthma* to *U_hormonal exposures*). In summary, we assumed that the potential measurement errors in our study were both *dependent* and *differential*.[74]

eReferences

1. Hernán MA. The C-word: scientific euphemisms do not improve causal inference from observational data. Am J Public Health. 2018;108(5):616-619.
2. Nwaru BI, Ekerljung L, Rådinger M, et al. Cohort profile: the West Sweden Asthma Study (WSAS): a multidisciplinary population-based longitudinal study of asthma, allergy and respiratory conditions in adults. BMJ Open. 2019;9(6):e027808.
3. Hernán MA, Robins JM. Chapter 6: Graphical representation of causal effects. In Causal Inference: What If. Boca Raton: Chapman & Hall/CRC. 2020.
4. Hernán MA, Robins JM. Chapter 7: Confounding. In Causal Inference: What If. Boca Raton: Chapman & Hall/CRC. 2020.
5. Hernán MA, Robins JM. Chapter 8: Selection bias. In Causal Inference: What If. Boca Raton: Chapman & Hall/CRC. 2020.
6. Hernán MA, Hernández-Díaz S, Robins JM. A structural approach to selection bias. Epidemiology. 2004;15(5):615-625.
7. Hernán MA, Robins JM. Chapter 18: Variable selection for causal inference. In Causal Inference: What If. Boca Raton: Chapman & Hall/CRC. 2020.
8. Hernán MA, Hernández-Díaz S, Werler MM, et al. Causal knowledge as a prerequisite for confounding evaluation: an application to birth defects epidemiology. Am J Epidemiol. 2002;155(2):176-184.
9. Suttorp MM, Siegerink B, Jager KJ, et al. Graphical presentation of confounding in directed acyclic graphs. Nephrol Dial Transplant. 2015;30(9):1418-1423.
10. Shrier I, Platt RW. Reducing bias through directed acyclic graphs. BMC Med Res Methodol. 2008;8:70.
11. Williamson EJ, Aitken Z, Lawrie J, et al. Introduction to causal diagrams for confounder selection. Respirology. 2014;19(3):303-311.
12. Pearl J. Causal diagrams for empirical research. Biometrika. 1995;82(4):669-710.
13. Hernán MA, Robins JM. Chapter 22: Target trial emulation. In Causal Inference: What If. Boca Raton: Chapman & Hall/CRC. 2020.
14. Textor J, van der Zander B, Gilthorpe MS, et al. Robust causal inference using directed acyclic graphs: the R package 'dagitty.' Int J Epidemiol. 2016;45(6):1887-1894.
15. Kleinbaum DG, Klein M. Chapter 11: Analysis of matched data using logistic regression. In Logistic Regression: A Self-Learning Text (Third Edition). Springer Science+Business Media, LLC 2010.
16. Rose S, Laan MJ. Why match? Investigating matched case-control study designs with causal effect estimation. Int J Biostat. 2009;5(1):Article 1.
17. White IR, Carlin JB. Bias and efficiency of multiple imputation compared with complete-case analysis for missing covariate values. Stat Med. 2010;29(28):2920-2931.
18. Sterne JA, White IR, Carlin JB, et al. Multiple imputation for missing data in epidemiological and clinical research: potential and pitfalls. BMJ. 2009;338:b2393.
19. White IR, Royston P, Wood AM. Multiple imputation using chained equations: issues and guidance for practice. Stat Med. 2011;30(4):377-399.
20. Rubin DB. Multiple Imputation for Nonresponse in Surveys. John Wiley & Sons, Inc. 1987.
21. van Buuren S. Chapter 5: Analysis of imputed data. In Flexible Imputation of Missing Data (Second Edition). Chapman & Hall/CRC. 2018.
22. Seaman SR, Keogh RH. Handling missing data in matched case-control studies using multiple imputation. Biometrics. 2015;71(4):1150-1159.
23. van Buuren S, Groothuis-Oudshoorn K. mice: Multivariate Imputation by Chained Equations in R. J Stat Softw. 2011;45(3):1-67.
24. Azur MJ, Stuart EA, Frangakis C, et al. Multiple imputation by chained equations: what is it and how does it work? Int J Methods Psychiatr Res. 2011;20(1):40-49.
25. Moons KG, Donders RA, Stijnen T, et al. Using the outcome for imputation of missing predictor values was preferred. J Clin Epidemiol. 2006;59(10):1092-1101.
26. Mustillo S, Kwon S. Auxiliary variables in multiple imputation when data are missing not at random. J Math Sociol. 2015;39(2):73-91.
27. Pearce N. Analysis of matched case-control studies. BMJ. 2016;352:i969.
28. Kleinbaum DG, Klein M. Chapter 4: Maximum likelihood techniques: an overview. In Logistic Regression, Statistics for Biology and Health (Third Edition). Springer Science+Business Media, LLC 2010.

29. Wan F, Colditz GA, Sutcliffe S. Matched versus unmatched analysis of matched case-control studies. Am J Epidemiol. 2021;190(9):1859-1866.
30. Mansournia MA, Hernán MA, Greenland S. Matched designs and causal diagrams. Int J Epidemiol. 2013;42(3):860-869.
31. Brookmeyer R, Liang KY, Linet M. Matched case-control designs and overmatched analyses. Am J Epidemiol. 1986;124(4):693-701.
32. Brenner H, Blettner M. Controlling for continuous confounders in epidemiologic research. Epidemiology. 1997;8(4):429-434.
33. Austin PC, Brunner LJ. Inflation of the type I error rate when a continuous confounding variable is categorized in logistic regression analyses. Stat Med. 2004;23(7):1159-1178.
34. Persson E, Waernbaum I. Estimating a marginal causal odds ratio in a case-control design: analyzing the effect of low birth weight on the risk of type 1 diabetes mellitus. Stat Med. 2013;32(14):2500-2512.
35. McElreath R. Statistical Rethinking: A Bayesian Course with Examples in R and Stan (Second Edition). Chapman & Hall/CRC. 2020.
36. Greenland S. Bayesian perspectives for epidemiological research: I. Foundations and basic methods. Int J Epidemiol. 2006;35(3):765-775.
37. Muth C, Oravecz Z, Gabry J. User-friendly Bayesian regression modeling: a tutorial with rstanarm and shinystan. TQMP. 2018;14(2):99-119.
38. MacLehose RF, Hamra GB. Applications of Bayesian methods to epidemiologic research. Curr Epidemiol Rep. 2014;1(3):103-109.
39. Nicenboim B, Schad D, Vasishth S. Chapter 6: The art and science of prior elicitation. In An Introduction to Bayesian Data Analysis for Cognitive Science. Available from: https://vasishth.github.io/bayescogsci/book/ch-priors.html.
40. Mathur MB, VanderWeele TJ. Robust metrics and sensitivity analyses for meta-analyses of heterogeneous effects. Epidemiology. 2020;31(3):356-358.
41. Pullenayegum EM. An informed reference prior for between-study heterogeneity in meta-analyses of binary outcomes. Stat Med. 2011;30(26):3082-3094.
42. Zhang GQ, Özuygur Ermis SS, Rådinger M, et al. Sex disparities in asthma development and clinical outcomes: implications for treatment strategies. J Asthma Allergy. 2022;15:231-247.
43. McCleary N, Nwaru BI, Nurmatov UB, et al. Endogenous and exogenous sex steroid hormones in asthma and allergy in females: a systematic review and meta-analysis. J Allergy Clin Immunol. 2018;141(4):1510-1513.
44. Zhang GQ, Chen JL, Luo Y, et al. Menopausal hormone therapy and women's health: an umbrella review. PLoS Med. 2021;18(8):e1003731.
45. Depaoli S, Winter SD, Visser M. The importance of prior sensitivity analysis in Bayesian statistics: demonstrations using an interactive Shiny app. Front Psychol. 2020;11:608045.
46. Goodrich B, Gabry J, Ali I, et al. rstanarm: Bayesian applied regression modeling via Stan. R package version 2.21.1. 2020. Available from: https://mc-stan.org/rstanarm.
47. Lemoine NP. Moving beyond noninformative priors: why and how to choose weakly informative priors in Bayesian analyses. Oikos. 2019;128(7):912-928.
48. Zhou X, Reiter JP. A note on Bayesian inference after multiple imputation. Am Stat. 2010;64(2):159-163.
49. Zitzmann S, Hecht M. Going beyond convergence in Bayesian estimation: why precision matters too and how to assess it. Struct Equ Model. 2019;26(4):646-661.
50. Makowski D, Ben-Shachar MS, Lüdecke D. bayestestR: describing effects and their uncertainty, existence and significance within the Bayesian framework. J Open Source Softw. 2019;4(40):1541.
51. VanderWeele TJ, Ding P. Sensitivity analysis in observational research: introducing the E-value. Ann Intern Med. 2017;167(4):268-274.
52. Velentzis LS, Egger S, Banks E, et al. Menopausal hormone therapy: characterising users in an Australian national cross-sectional study. PLoS One. 2021;16(8):e0253725.
53. Costanian C, Edgell H, Ardern CI, et al. Hormone therapy use in the Canadian Longitudinal Study on Aging: a cross-sectional analysis. Menopause. 2018;25(1):46-53.
54. Manzoli L, Di Giovanni P, Del Duca L, et al. Use of hormone replacement therapy in Italian women aged 50-70 years. Maturitas. 2004;49(3):241-251.
55. Nagel G, Lahmann PH, Schulz M, et al. Use of hormone replacement therapy (HRT) among women aged 45-64 years in the German EPIC-cohorts. Maturitas. 2007;56(4):436-446.
56. Skiba MA, Islam RM, Bell RJ, et al. Hormonal contraceptive use in Australian women: who is using what? Aust N Z J Obstet Gynaecol. 2019;59(5):717-724.

57. Al Sheeha M. Awareness and use of contraceptives among saudi women attending primary care centers in Al-qassim, saudi arabia. Int J Health Sci. 2010;4(1):11-21.
58. Costas L, Sequera VG, Quesada P, et al. Hormonal contraception and postmenopausal hormone therapy in Spain: time trends and patterns of use. Menopause. 2015;22(10):1138-1146.
59. Parazzini F. Trends of determinants of hormone therapy use in Italian women attending menopause clinics, 1997-2003. Menopause. 2008;15(1):164-170.
60. Lucas R, Barros H. Life prevalence and determinants of hormone replacement therapy in women living in Porto, Portugal. Maturitas. 2007;57(3):226-232.
61. Lindh I, Hognert H, Milsom I. The changing pattern of contraceptive use and pregnancies in four generations of young women. Acta Obstet Gynecol Scand. 2016;95(11):1264-1272.
62. Endalifer ML, Diress G, Addisu A, et al. The association between combined oral contraceptive use and overweight/obesity: a secondary data analysis of the 2016 Ethiopia Demographic and Health Survey. BMJ Open. 2020;10(12):e039229.
63. San-Juan-Rodriguez A, Bes-Rastrollo M, Martinez-Gonzalez MA, et al. Oral contraceptives use and development of obesity in a Mediterranean cohort: the SUN (Seguimiento Universidad de Navarra) Project. Int J Obes. 2020;44(2):320-329.
64. Ulhaq MZ, Pamungkasari EP, Murti B. Meta Analysis: effect of oral contraceptives on obesity in fertile age women in Asia. Journal of Maternal and Child Health. 2020;5(6):673-682.
65. Anderson DJ, Chung HF, Seib CA, et al. Obesity, smoking, and risk of vasomotor menopausal symptoms: a pooled analysis of eight cohort studies. Am J Obstet Gynecol. 2020;222(5):478.
66. Noll P, Campos CAS, Leone C, et al. Dietary intake and menopausal symptoms in postmenopausal women: a systematic review. Climacteric. 2021;24(2):128-138.
67. Canário AC, Cabral PU, Spyrides MH, et al. The impact of physical activity on menopausal symptoms in middle-aged women. Int J Gynaecol Obstet. 2012;118(1):34-36.
68. McAndrew LM, Napolitano MA, Albrecht A, et al. When, why and for whom there is a relationship between physical activity and menopause symptoms. Maturitas. 2009;64(2):119-125.
69. Dąbrowska-Galas M, Dąbrowska J, Ptaszkowski K, et al. High physical activity level may reduce menopausal symptoms. Medicina (Kaunas). 2019;55(8):466.
70. Sabia S, Fournier A, Mesrine S, et al. Risk factors for onset of menopausal symptoms: results from a large cohort study. Maturitas. 2008;60(2):108-121.
71. Beasley R, Semprini A, Mitchell EA. Risk factors for asthma: is prevention possible? Lancet. 2015;386(9998):1075-1085.
72. Ilmarinen P, Tuomisto LE, Kankaanranta H. Phenotypes, risk factors, and mechanisms of adult-onset asthma. Mediators Inflamm. 2015;2015:514868.
73. Macsali F, Svanes C, Bjørge L, et al. Respiratory health in women: from menarche to menopause. Expert Rev Respir Med. 2012;6(2):187-200.
74. Hernán MA, Robins JM. Chapter 9: Measurement bias. In Causal Inference: What If. Boca Raton: Chapman & Hall/CRC. 2020.
