# Supplementary material for: Exogenous female sex steroid hormones and new-onset asthma in women: a matched case–control study
Source: BMC Med. 2023 Sep 4;21:337. doi: 10.1186/s12916-023-03038-8 (PMC10478448; doi:10.1186/s12916-023-03038-8)
Supplement: Supplementary file 3 — Additional file 3: Section 1. Supplementary methods and results for the Frequentist analysis of hormonal contraceptives and new-onset asthma in women. Table S1. Background characteristics of the responded new-onset asthma cases and matched controls in WSAS in 2009–2016. Figure S1. Age distribution at baseline in 2008 among the responded women (N = 353). Figure S2. Age at first use of hormonal contraceptives in 261 women. Figure S3. Age at asthma diagnosis among 32 cases. Figure S4. Ever use of hormonal contraceptives (compared to never use) and new-onset asthma in women. Figure S5. Comparison between women who had data on the incomplete variables and those who did not with respect to auxiliary variables. Figure S6. Healthy convergence for the incomplete variables. Figure S7. The distribution of use of hormonal contraceptives in the observed data and in the m = 100 imputed datasets. Figure S8. Kernel density estimates for the marginal distributions of the observed data for age at menarche and the m = 100 densities calculated from the imputed data. Figure S9. The distribution of gynecological condition in the observed data and in the m = 100 imputed datasets. Figure S10. The distribution of level of education in the observed data and in the m = 100 imputed datasets. Figure S11. Odds ratios for ever use of hormonal contraceptives (compared to never use) and new-onset asthma among all women (N = 831) across the m = 100 imputed datasets. Section 2. Supplementary methods and results for the Frequentist analysis of menopausal hormone therapy and new-onset asthma in menopausal women. Figure S12. Age at first use of menopausal hormone therapy in 32 women. Figure S13. Age at asthma diagnosis among 13 cases. Figure S14. Ever use of menopausal hormone therapy (compared to never use) and new-onset asthma in menopausal women. Figure S15. Comparison between women who had data on the incomplete variables and those who did not with respect to auxiliary variables. Figure S16. Healthy [file 12916_2023_3038_MOESM3_ESM.pdf]

Additional File 3
Supplementary Methods and Results
# – the *Frequentist* approach

The Additional file 3 contains the supplementary methods and results from the Frequentist analysis for:

Guo-Qiang Zhang*, Rani Basna, Maya B. Mathur, Cecilia Lässer, Roxana Mincheva, Linda Ekerljung, Göran Wennergren, Madeleine Rådinger, Bo Lundbäck, Hannu Kankaanranta, Bright I. Nwaru. Exogenous female sex steroid hormones and new-onset asthma in women: a matched case-control study. 2023.

* Corresponding author
Krefting Research Centre, Department of Internal Medicine and Clinical Nutrition, Institute of Medicine, Sahlgrenska Academy, University of Gothenburg, Gothenburg, Sweden
E-mail: guo-qiang.zhang@gu.se

[The supplementary Tables and Figures are attached at the end of the document, and hyperlinked within the text to allow for easy navigation between the text and the supplementary Tables and Figures.]

Table of Contents

HORMONAL CONTRACEPTIVES AND NEW-ONSET ASTHMA IN WOMEN 3

1. PECOS COMPONENTS 3
2. ADJUSTMENT VARIABLES 3
3. COMPLETE-CASE ANALYSIS 4
4. MULTIPLE IMPUTATION ANALYSIS 4
4.1. "INCOMPLETE" DATASET 4
4.2. SELECT PREDICTORS FOR IMPUTATION MODEL 4
4.3. VALIDATE IMPUTATIONS 4
4.4. FREQUENTIST CONDITIONAL LOGISTIC REGRESSION 5

MENOPAUSAL HORMONE THERAPY AND NEW-ONSET ASTHMA IN MENOPAUSAL WOMEN 6

1. PECOS COMPONENTS 6
2. ADJUSTMENT VARIABLES 6
3. COMPLETE-CASE ANALYSIS 7
4. MULTIPLE IMPUTATION ANALYSIS 7
4.1. "INCOMPLETE" DATASET 7
4.2. SELECT PREDICTORS FOR IMPUTATION MODEL 7
4.3. VALIDATE IMPUTATIONS 7
4.4. FREQUENTIST CONDITIONAL LOGISTIC REGRESSION 7

SUPPLEMENTARY TABLES AND FIGURES 8

1. HORMONAL CONTRACEPTIVES AND NEW-ONSET ASTHMA IN WOMEN 10
2. MENOPAUSAL HORMONE THERAPY AND NEW-ONSET ASTHMA IN MENOPAUSAL WOMEN 20

EREFERENCES 34

Hormonal Contraceptives and New-Onset Asthma in Women

1. PECOS Components

- **Population**: a total of 353 women, including 72 new-onset asthma cases and 281 matched controls; the median age at baseline was 46 years (range: 19–74 years); the age distribution is shown in Figure S1; the background characteristics of the responded cases and matched controls are presented in Table S1.
- **Exposure**: ever use of hormonal contraceptives; 266 women ever used hormonal contraceptives, while 75 never used (12 had missing data); out of the 266 women, 239 used pills, 114 used vaginal rings, and eight used injections; the median age when starting hormonal contraceptives was 18 years (range: 13–49 years; 261 women, five had missing data); all of them had started hormonal contraceptives by the year 2016 (i.e., the end of follow-up in WSAS); the distribution for age of starting hormonal contraceptives is illustrated in Figure S2.
- **Comparator**: never use of hormonal contraceptives.
- **Outcome**: new-onset asthma after use of hormonal contraceptives; the mean age of asthma diagnosis was 44 years (range: 23–68 years; 32 cases, 40 had missing data); among the 32 cases who had data on age at asthma diagnosis, 28 were in the exposure group and developed asthma after they had started using hormonal contraceptives; the remaining four cases did not ever use hormonal contraceptives (i.e., in the comparator group) and developed asthma after their corresponding exposure group had started using hormonal contraceptives; the distribution for age at asthma diagnosis is shown in Figure S3.
- **Study design**: a matched case-control design.

2. Adjustment Variables

The variables that need to be adjusted for to eliminate confounding include age, adulthood socioeconomic status (SES), age at menarche, and gynecological conditions (see Additional file 2: Figure S1). The definitions of the adjusted variables are as below:

- Age: exact age in years in 2008.
- Adulthood SES: we used place of residence in 2008 and level of education in 2016 as proxies for adulthood SES; place of residence was categorized into Gothenburg area and outside Gothenburg; level of education was categorized into less than high school, high school, and tertiary level (i.e., university or other corresponding post high school level).
- Age at menarche: age in years at the first menstrual period based on the Women's Questionnaire survey in 2018–2020.
- Gynecological conditions: including endometriosis, polycystic ovarian syndrome, gynecological acne, and hysterectomy with or without oophorectomy, based on the Women's Questionnaire survey in 2018–2020; categorized into "Yes" and "No".
- Tobacco smoking: categorized into never smoker, former smoker and current smoker, based on the smoking status in 2016; because smoking was one of the matching variables, smoking was *unavoidably* adjusted for when adjusting for the matching sets during the analyses.

3. Complete-Case Analysis

Frequentist conditional logistic regression was used to adjust for confounding variables. The analyses were restricted only to women who had complete data (N = 315). Exploratory subgroup analyses were conducted by baseline age. The results are presented in Figure S4. The E-value for the point estimate among all women was 3.43.

4. Multiple Imputation Analysis

4.1. “Incomplete” dataset

In total, among the 6,295 women who had never had asthma in 2008 in WSAS cohort, 114 developed new-onset asthma during the period 2009–2016 and were invited to the Women’s Questionnaire survey in 2018–2020. Seventy-two of the 114 cases (63.2%) responded and were matched with 602 controls by age, smoking and place of residence (≈ 1:10) (Figure 1). The choice of a relatively high number of controls per case was to account for potential non-response among the matched controls. For the 42 cases who did not respond to the survey, 13 had identical values for the matching variables as the matched case-control sets. Thus, they were added to the corresponding matched sets. For the remaining 29 cases, each was matched by matching variables to four controls.[1] In stratum where the number of controls in the population was equal to or less than four, all controls were selected for that stratum. Finally, the matching resulted in an “incomplete” dataset (due to missingness in some key non-matching variables) comprising 114 cases and 717 controls. The median number of controls per case was seven (range: 3–10). Table 1 summarizes background characteristics for all the cases and matched controls.

4.2. Select predictors for imputation model

We included all variables (including outcome variable) that were in the final analytic model as predictors in the imputation model (see Additional file 2: Section 4.1 for justifications). These consisted of age, smoking, place of residence, asthma, use of hormonal contraceptives, age at menarche, gynecological conditions, and level of education. In addition, we selected auxiliary variables for the incomplete variables (use of hormonal contraceptives, age at menarche, gynecological conditions, and level of education) in the analysis model, including occupation, body mass index (BMI), and physical exercise. All auxiliary variables either correlated with the incomplete variables and/or predicted the missingness of the incomplete variables. Figure S5 presents the comparison between individuals who had data on the incomplete variables and those who did not with regards to the auxiliary variables.

4.3. Validate imputations

- Assess convergence: the convergence for use of hormonal contraceptives, age at menarche, gynecological conditions, and level of education is shown in Figure S6.
- Visualize distributions of imputed data: the distributions of the imputed values for the incomplete variables are shown from Figure S7 to Figure S10.

4.4. Frequentist conditional logistic regression

The analyses were conducted among all women (N = 831) and by different age groups. Figure S11 illustrates the range of odds ratios for ever use of hormonal contraceptives (compared to never use) and new-onset asthma among all women across the m = 100 imputed datasets. The overall results and subgroup analyses by baseline age are presented in Figure 2A.

Menopausal Hormone Therapy and New-Onset Asthma in Menopausal Women

1. PECOS Components

- **Population**: a total of 185 women aged ≥ 45 years at baseline in 2008, including 35 new-onset asthma cases and 150 matched controls; the median age at baseline was 54 years (range: 45–74 years); the age distribution is shown in Figure S1.
- **Exposure**: ever use of menopausal hormone therapy (MHT); 37 women ever used MHT, while 146 never used (two had missing data); when starting using MHT, 30 women had hot flashes and/or night sweats, six had palpitations, five had problems with memory or concentration, and one had joint aches; the median age of starting MHT was 52 years (range: 38–64 years; 34 women, three had missing data); all of them except four controls had started MHT by the year 2016 (i.e., the end of follow-up in WSAS); thus, the four controls were taken as having never used MHT; the distribution for age of starting MHT is illustrated in Figure S12.
- **Comparator**: never use of MHT.
- **Outcome**: new-onset asthma after use of MHT; among the 35 new-onset asthma cases, 13 reported age at asthma diagnosis (median: 56 years, range: 46–68 years; 22 had missing data; Figure S13); among the 13 cases, one was in the exposure group and developed asthma after they had started using MHT; the remaining 12 cases did not ever use MHT (i.e., in the comparator group) and developed asthma after their corresponding exposure group had started using MHT.
- **Study design**: a matched case-control design.

2. Adjustment Variables

The variables that need to be adjusted for to eliminate confounding include age, SES, tobacco smoking, age at menopause, BMI, physical activity, environmental tobacco smoke, gynecological conditions, alcohol, and diet (see Additional file 2: Figure S2). In our study, we had data on all the variables except alcohol and diet. The definitions of the adjusted variables are as below:

- Age: exact age in years in 2008.
- Adulthood SES: we used place of residence in 2008 and level of education in 2016 as proxies for adulthood SES; place of residence was categorized into Gothenburg area and outside Gothenburg; level of education was categorized into less than high school, high school, and tertiary level (i.e., university or other corresponding post high school level).
- BMI: defined as weight (kg) divided by the square of height (m), based on the 2008 survey data.
- Tobacco smoking: categorized into never smoker, former smoker and current smoker, based on the smoking status in 2016.
- Environmental tobacco smoke: ever heavily exposed to environmental tobacco smoke at work or at home, based on the 2016 survey data.
- Age at menopause: age in years at the last menstrual period, including natural and surgical menopause, based on the Women's Questionnaire survey in 2018–2020.

- Physical activity: number of hours per week for physical exercise, based on the 2008 survey data; categorized into never, 0.5 hour, one hour, two to three hours, and ≥ 4 hours.
- Gynecological conditions: including endometriosis, polycystic ovarian syndrome, gynecological acne, and hysterectomy with or without oophorectomy, based on the Women's Questionnaire survey in 2018–2020; categorized into "Yes" and "No".

3. Complete-Case Analysis

Frequentist conditional logistic regression was used to adjust for confounding variables. The analyses were restricted only to women who had complete data (N = 135). Exploratory subgroup analyses were conducted by baseline age. The results are presented in Figure S14. The E-value for the point estimate among all women was 1.49.

4. Multiple Imputation Analysis

4.1. "Incomplete" dataset

The "incomplete" dataset is described on page 4. In brief, it consisted of 54 cases and 355 matched controls aged ≥ 45 years at baseline in 2008. The median number of controls per case was eight (range: 3–10).

4.2. Select predictors for imputation model

We included all variables (including outcome variable) that were in the final analytic model as predictors in the imputation model (see Additional file 2: Section 4.1 for justifications). These consisted of age, place of residence, level of education, asthma, BMI, tobacco smoking, environmental tobacco smoke, use of MHT, age at menopause, physical exercise, and gynecological conditions. In addition, we selected auxiliary variables for the incomplete variables (level of education, BMI, environmental tobacco smoke, use of MHT, age at menopause, physical exercise, and gynecological conditions) in the analysis model, including occupation and hypertension. All auxiliary variables either correlated with the incomplete variables and/or predicted the missingness of the incomplete variables. Figure S15 presents the comparison between individuals who had data on the incomplete variables and those who did not with regards to the auxiliary variables.

4.3. Validate imputations

- Assess convergence: the convergence for level of education, BMI, environmental tobacco smoke, use of MHT, age at menopause, physical exercise, and gynecological conditions is shown in Figure S16.
- Visualize distributions of imputed data: the distributions of the imputed values for the incomplete variables are shown from Figure S17 to Figure S23.

4.4. Frequentist conditional logistic regression

The analyses were conducted among all menopausal women (N = 409) and by different age groups. Figure S24 illustrates the range of odds ratios for ever use of MHT (compared to never use) and new-onset asthma among all women across the m = 100 imputed datasets. The overall results and subgroup analyses by baseline age are presented in Figure 3A.

Supplementary Tables and Figures

Table S1. Background characteristics of the responded new-onset asthma cases and matched controls in WSAS in 2009–2016

Characteristics[a]	Cases (N = 72) n (%)	Controls (N = 281) n (%)	*P* value[b]
Age (y), mean (SD)	44.5 (14.6)	45.2 (13.6)	0.724
BMI (kg/m^2)			0.669
<25	50 (69.4)	185 (65.8)	
25–29.9	13 (18.1)	64 (22.8)	
≥30	8 (11.1)	28 (10.0)	
Missing	1 (1.4)	4 (1.4)	
Smoking status			0.522
Never smoker	41 (56.9)	151 (53.7)	
Former smoker	26 (36.1)	117 (41.6)	
Current smoker	5 (6.9)	13 (4.6)	
Place of residence			0.588
Gothenburg	42 (58.3)	175 (62.3)	
Outside Gothenburg	30 (41.7)	106 (37.7)	
Level of education			0.616
Less than high school	6 (8.3)	35 (12.5)	
High school	21 (29.2)	87 (31.0)	
Tertiary level	44 (61.1)	159 (56.6)	
Missing	1 (1.4)	–	
Use of hormonal contraceptives			0.077
No	10 (13.9)	65 (23.1)	
Yes	62 (86.1)	204 (72.6)	
Missing	–	12 (4.3)	
Use of MHT[c]			0.464
No	27 (77.1)	123 (82.0)	
Yes	8 (22.9)	25 (16.7)	
Missing	–	2 (1.3)	

Abbreviations: BMI, body mass index; MHT, menopausal hormone therapy; SD, standard deviation.

[a] Age, body mass index and place of residence were based on the 2008 questionnaire survey; smoking status and level of education were based on the 2016 questionnaire survey; the hormonal exposures were based on the Women's Questionnaire survey in 2018–2020.

[b] Student's *t*-test was used for continuous variables, and Fisher's exact test for categorical variables.

[c] Among 185 women aged ≥45 years at baseline in 2008.

1. Hormonal Contraceptives and New-Onset Asthma in Women

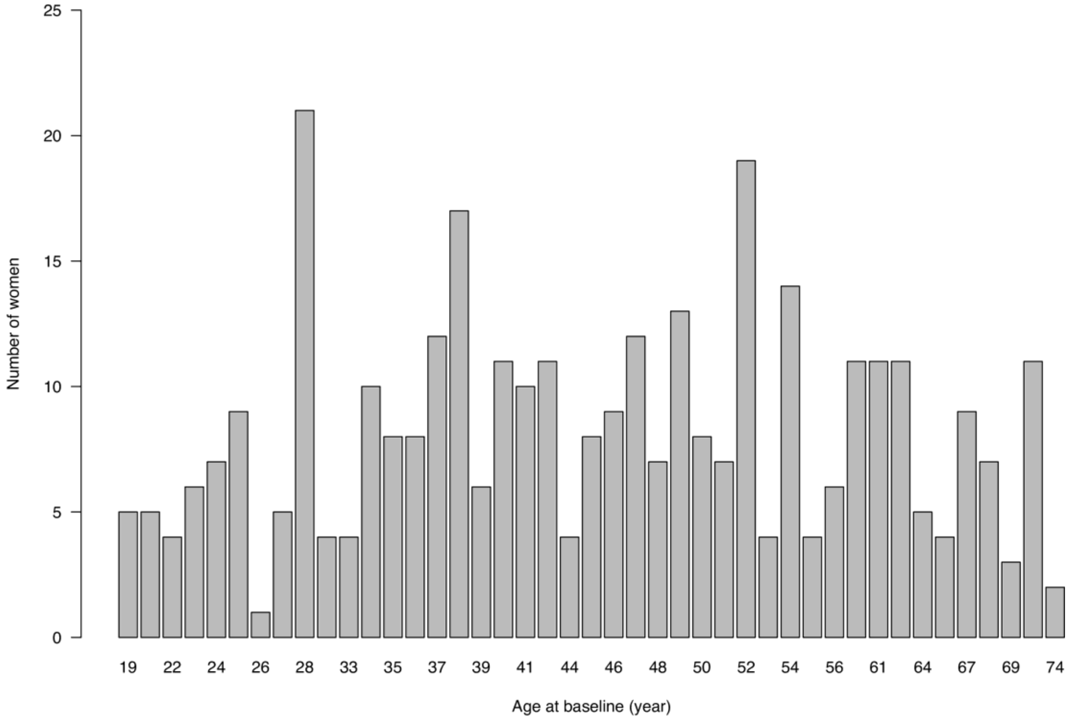

Figure S1. Age distribution at baseline in 2008 among the responded women (N = 353).

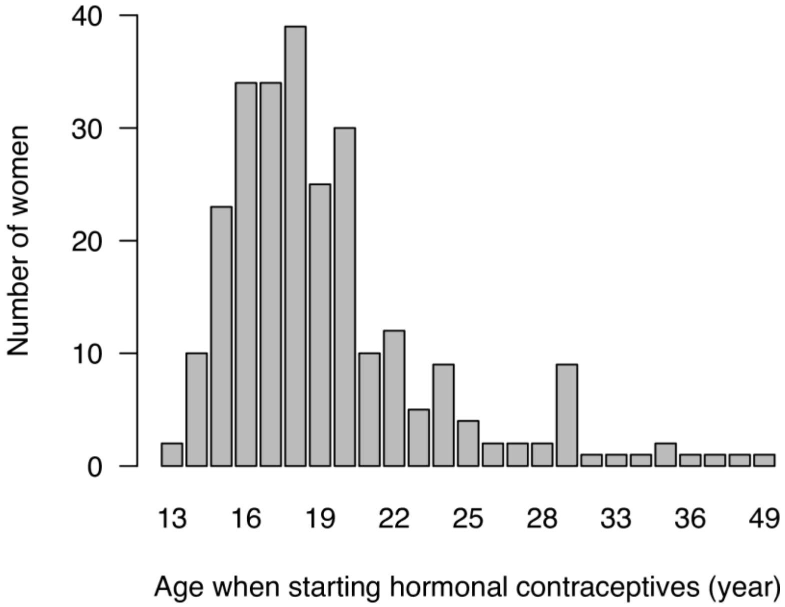

Figure S2. Age at first use of hormonal contraceptives in 261 women (five women had missing data).

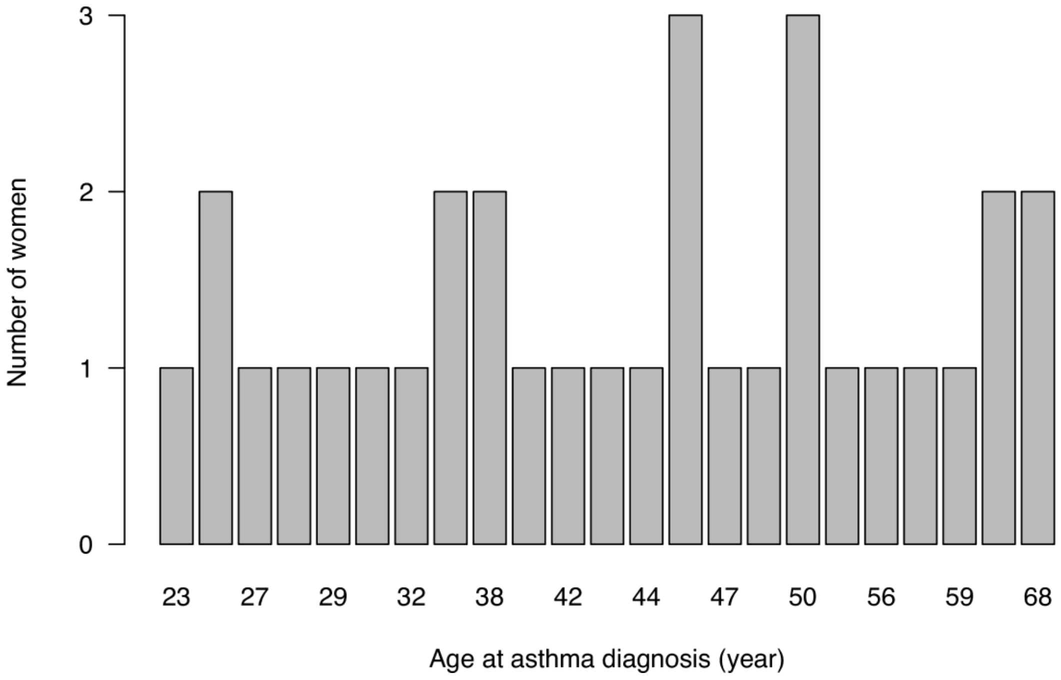

Figure S3. Age at asthma diagnosis among 32 cases (40 cases had missing data).

Age Group[a]	No. of Cases /Controls	Odds Ratio (95% CI)[b]
**Above an age cut-off**		
≥ 25 years	62/234	1.98 (0.88, 4.48)
≥ 35 years	50/193	2.67 (1.07, 6.64)
≥ 45 years	33/132	2.89 (0.95, 8.80)
≥ 55 years	17/53	3.36 (0.67, 16.84)
≥ 65 years	8/20	2.97 (0.27, 32.83)
**Below an age cut-off**		
≤ 25 years	8/20	Not estimable
≤ 35 years	20/59	0.67 (0.13, 3.51)
≤ 45 years	37/121	1.27 (0.38, 4.24)
≤ 55 years	52/197	1.28 (0.51, 3.18)
≤ 65 years	61/228	1.82 (0.76, 4.31)
**All women**	**68/247**	**2.01 (0.89, 4.51)**

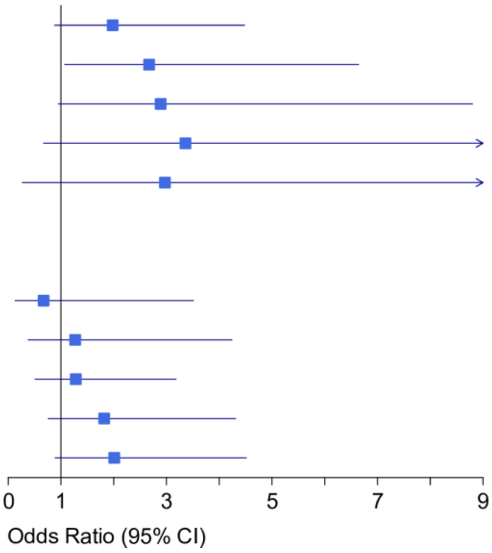

Figure S4. Ever use of hormonal contraceptives (compared to never use) and new-onset asthma in women.

The results were based on complete-case analysis of 315 women (excluding 38 women with missing data).
Abbreviations: CI, confidence interval.

[a] Age at baseline in 2008.

[b] Adjusted for age, place of residence, level of education, age at menarche, gynecological conditions, and tobacco smoking, using Frequentist conditional logistic regression.

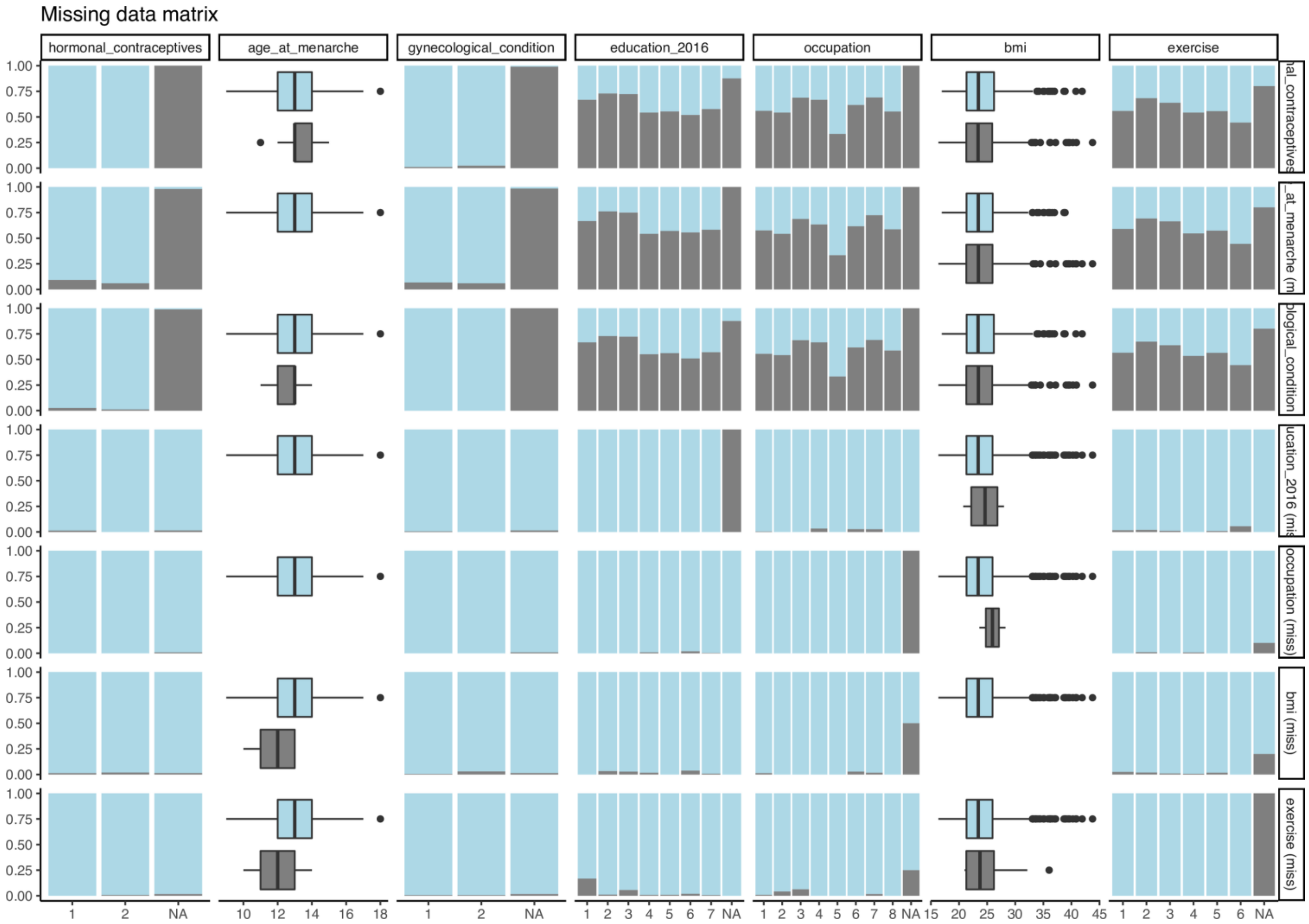

Figure S5. Comparison between women who had data on the incomplete variables and those who did not with respect to auxiliary variables.

Use of hormonal contraceptives, the first row; age at menarche, the second row; gynecological conditions, the third row; level of education, the fourth row. Auxiliary variables include occupation, body mass index, and physical exercise.

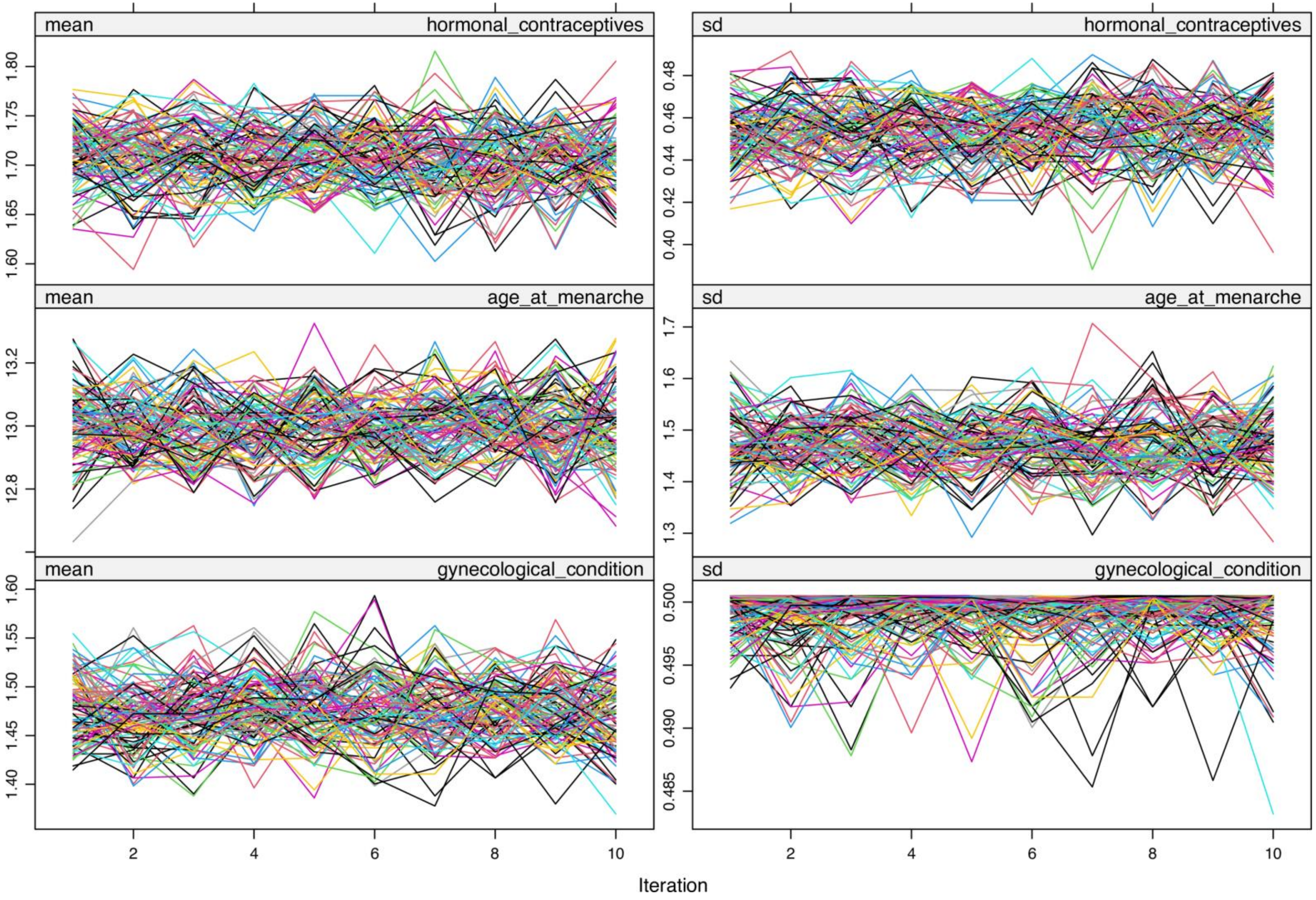

Figure S6. Healthy convergence for the incomplete variables.

Use of hormonal contraceptives, the first row; age at menarche, the second row; gynecological conditions, the third row; level of education, the fourth row.

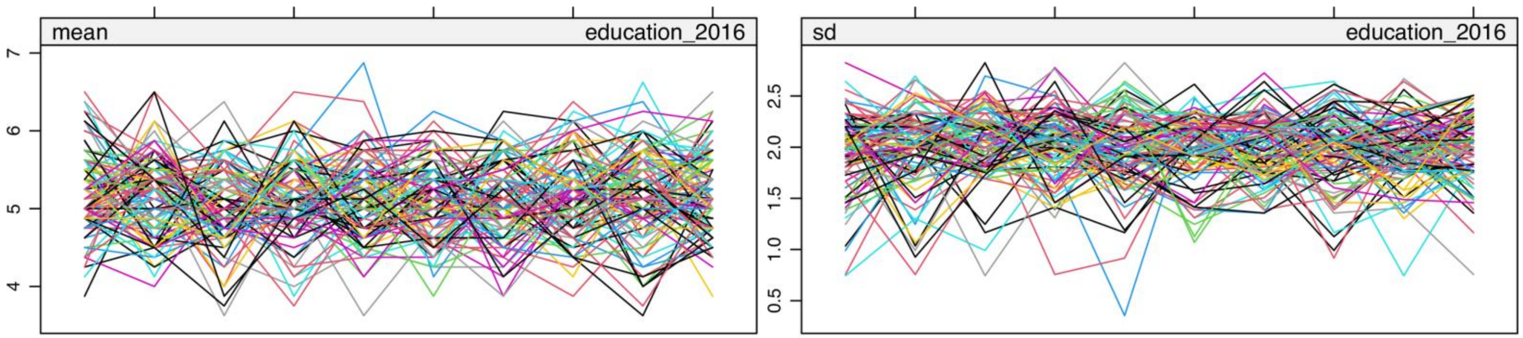

Figure S6. Healthy convergence for the incomplete variables (continued).

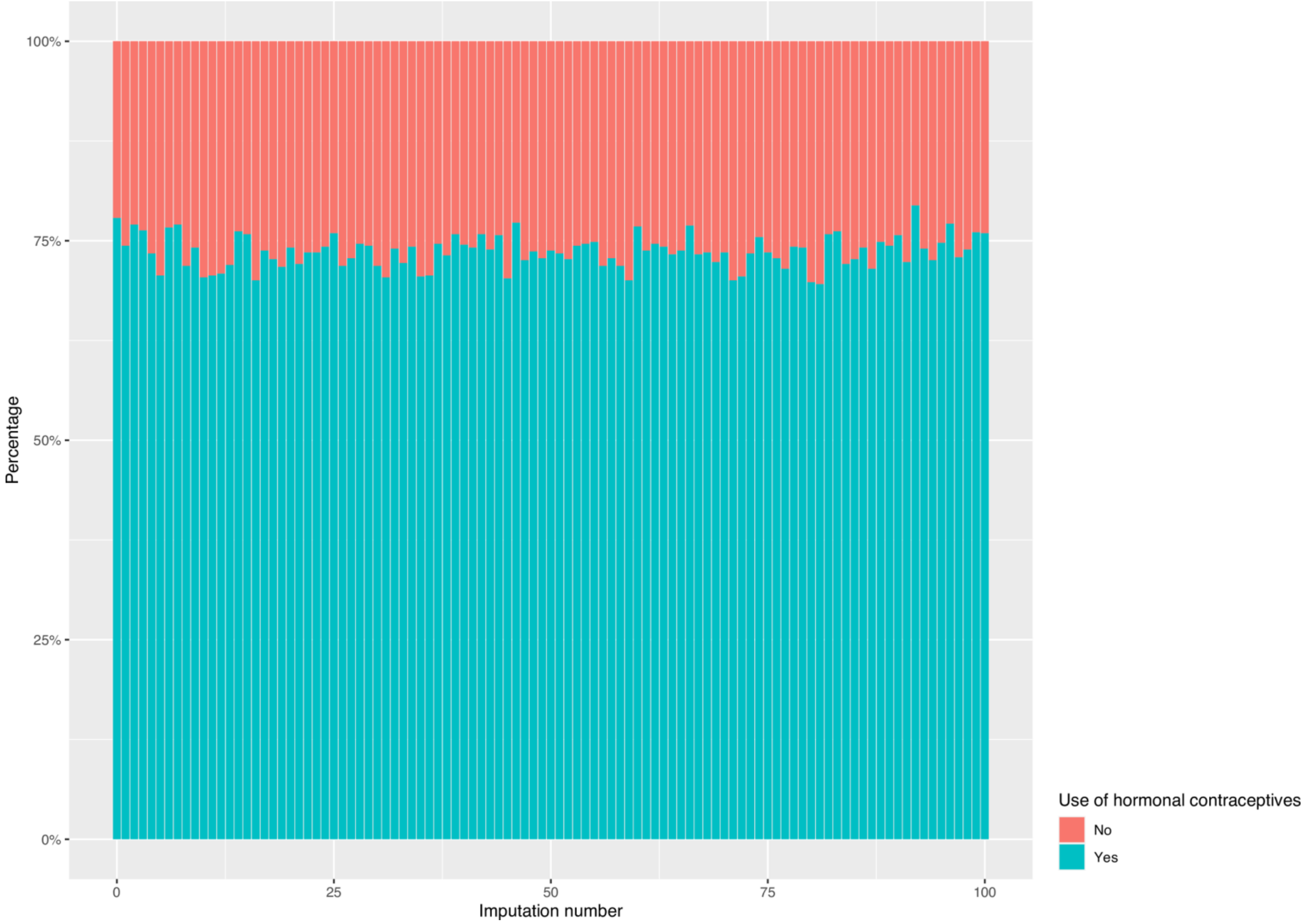

Figure S7. The distribution of use of hormonal contraceptives in the observed data and in the m = 100 imputed datasets.
Observed data (N = 343), imputation number = 0; imputed data (N = 831), imputation number from 1 to 100.

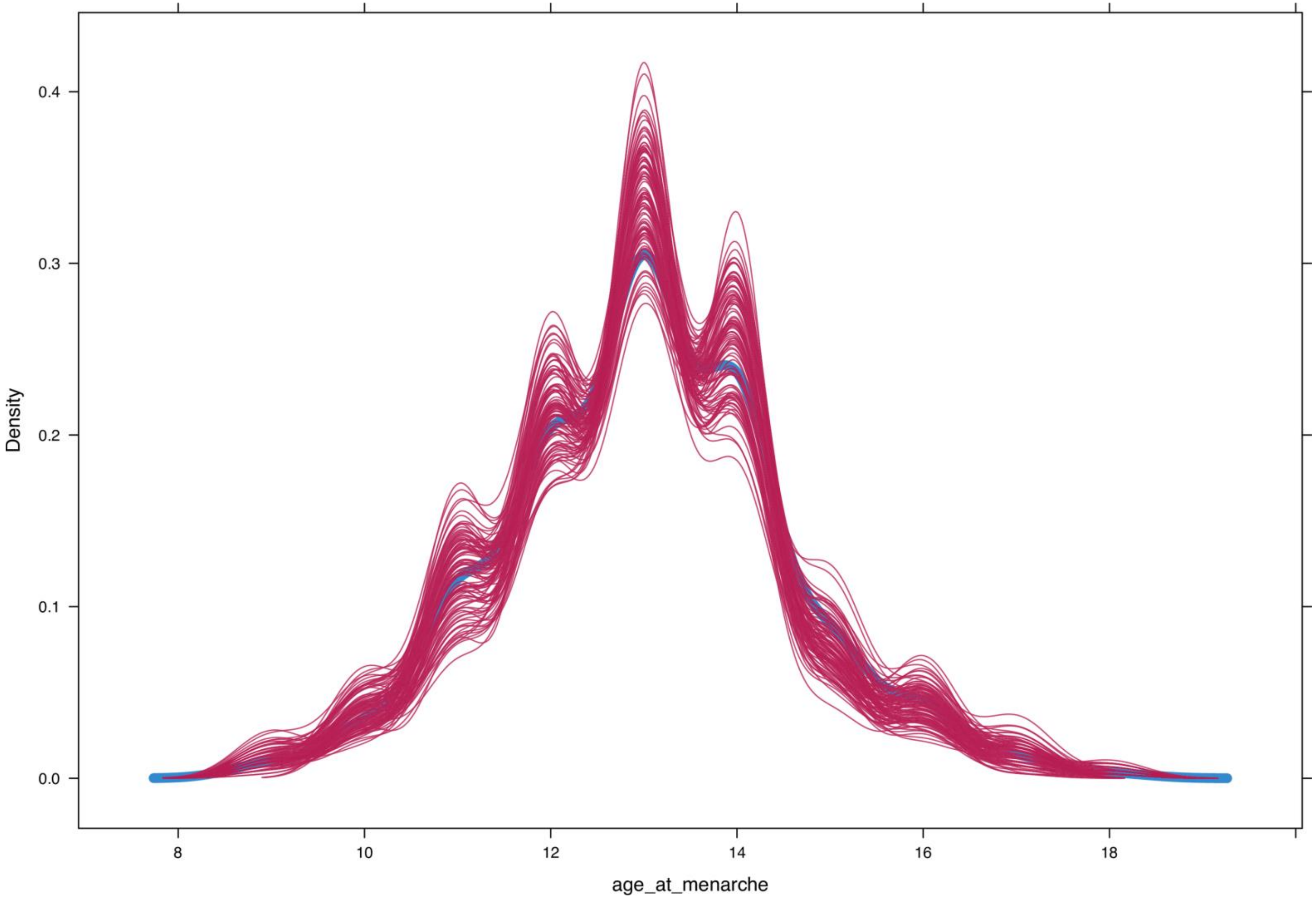

Figure S8. Kernel density estimates for the marginal distributions of the observed data for age at menarche and the m = 100 densities calculated from the imputed data.

Observed data (N = 330), thick blue line; imputed data (N = 831), thin red lines.

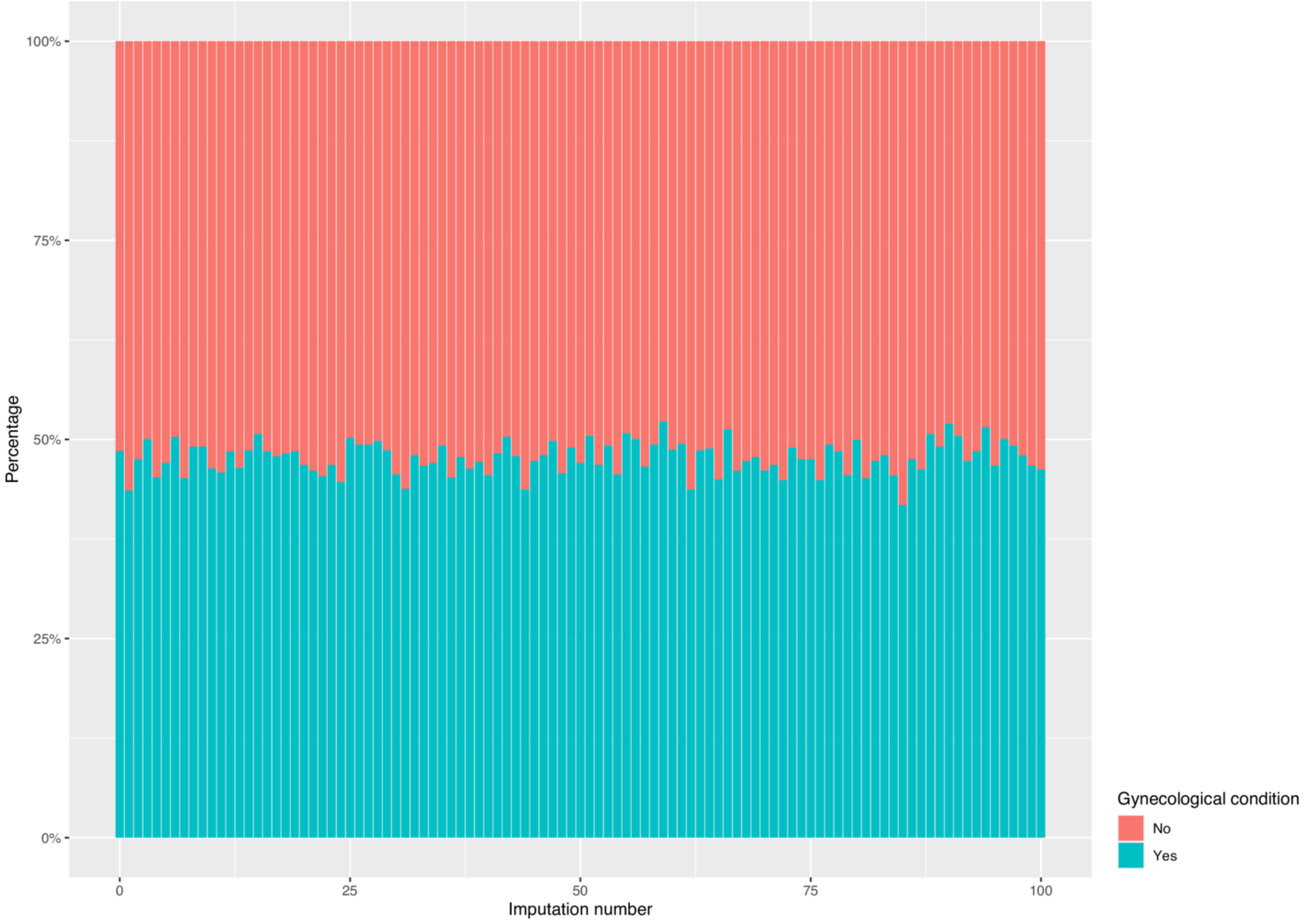

Figure S9. The distribution of gynecological condition in the observed data and in the m = 100 imputed datasets.
Observed data (N = 344), imputation number = 0; imputed data (N = 831), imputation number from 1 to 100.

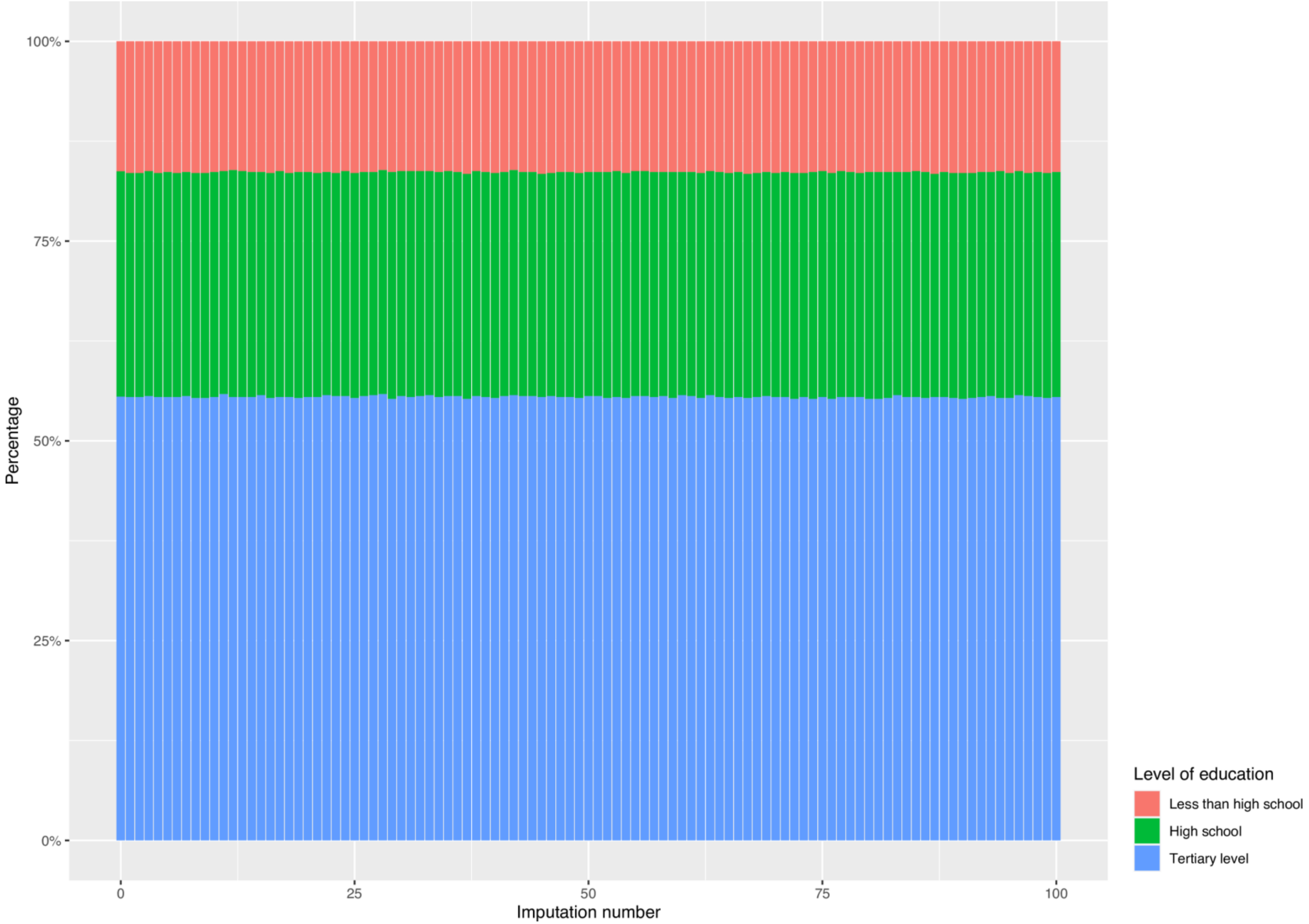

Figure S10. The distribution of level of education in the observed data and in the m = 100 imputed datasets.
Observed data (N = 823), imputation number = 0; imputed data (N = 831), imputation number from 1 to 100.

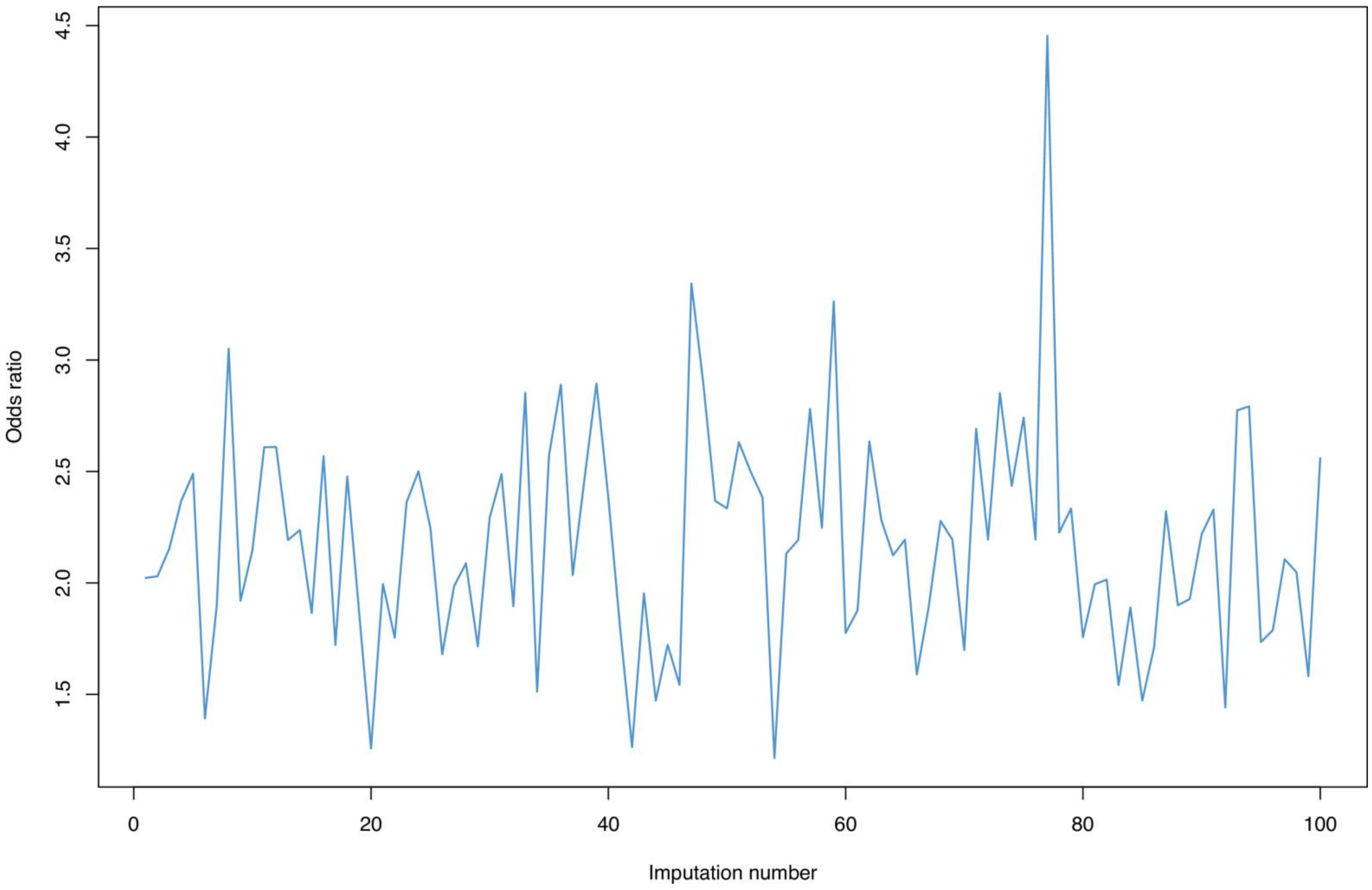

Figure S11. Odds ratios for ever use of hormonal contraceptives (compared to never use) and new-onset asthma among all women (N = 831) across the m = 100 imputed datasets.

Adjusted for age, place of residence, level of education, age at menarche, gynecological conditions, and tobacco smoking, using Frequentist conditional logistic regression.

2. Menopausal Hormone Therapy and New-Onset Asthma in Menopausal Women

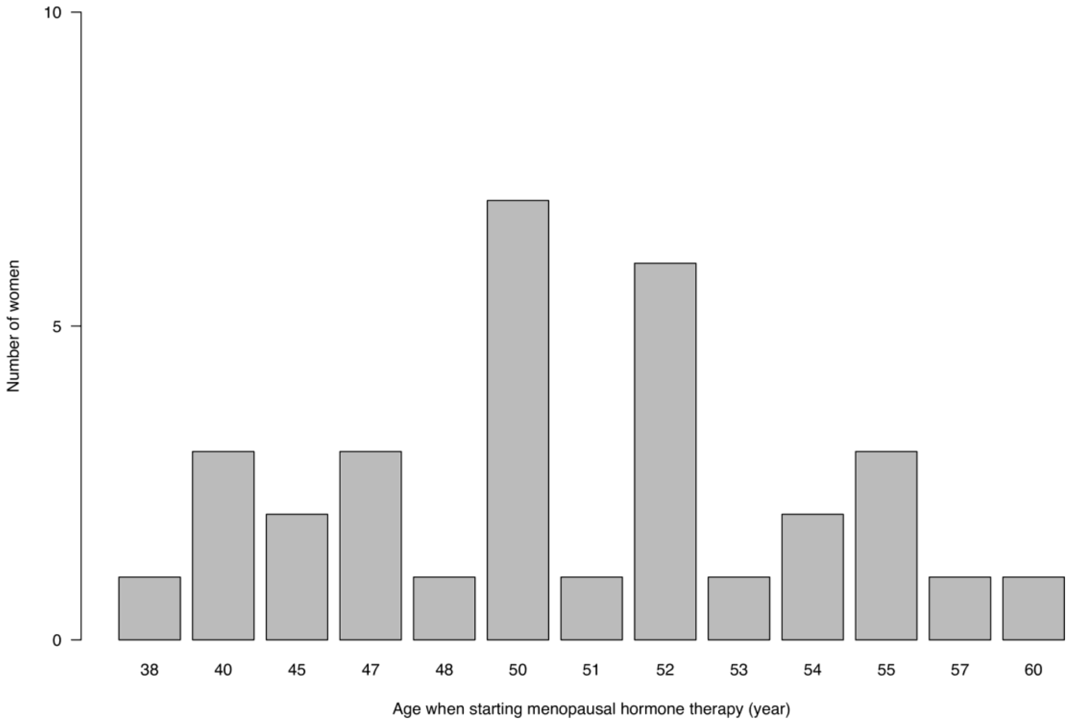

Figure S12. Age at first use of menopausal hormone therapy in 32 women (one woman had missing data).

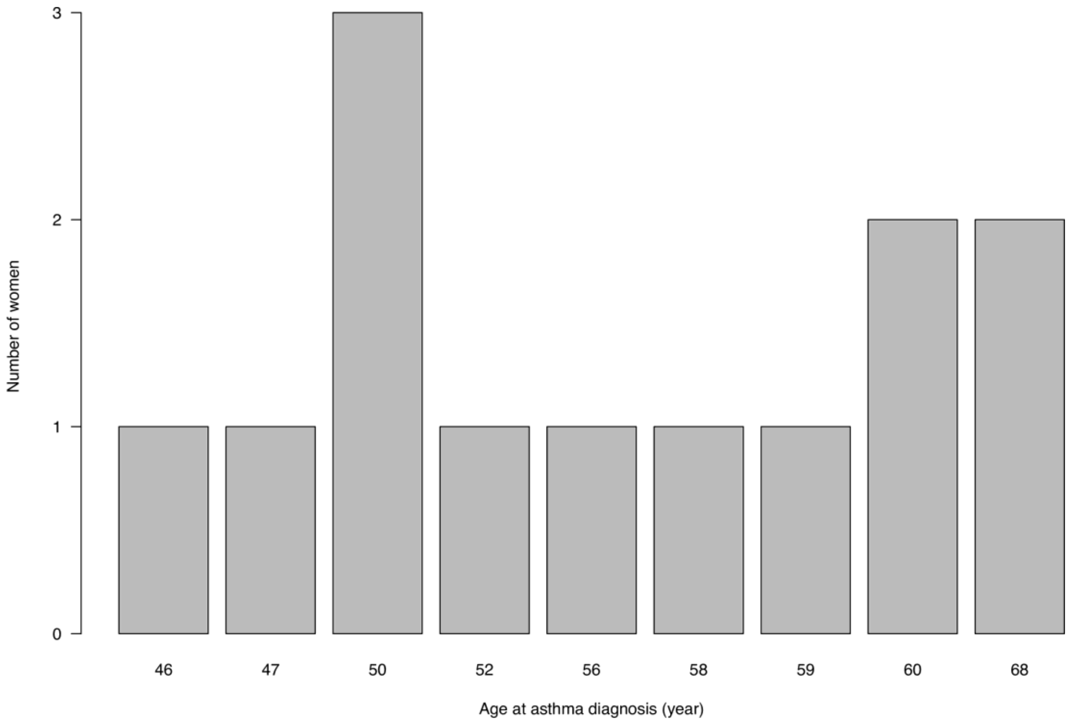

Figure S13. Age at asthma diagnosis among 13 cases (22 cases had missing data).

Age Group[a]	No. of Cases /Controls	Odds Ratio (95% CI)[b]
**Above an age cut-off**		
≥ 50 years	18/83	0.87 (0.18, 4.14)
≥ 55 years	12/52	0.38 (0.02, 7.95)
≥ 60 years	9/39	0.66 (0.01, 34.45)
≥ 65 years	5/21	Not estimable
**Below an age cut-off**		
≤ 50 years	4/33	Not estimable
≤ 55 years	10/64	4.55 (0.43, 47.64)
≤ 60 years	13/74	2.29 (0.36, 14.58)
≤ 65 years	17/93	1.11 (0.19, 6.48)
**All women**	**22/113**	**1.12 (0.26, 4.82)**

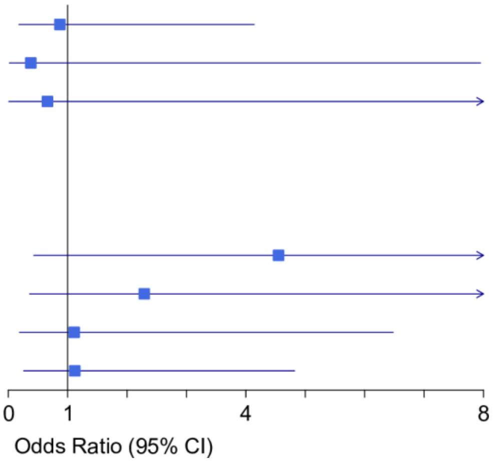

Figure S14. Ever use of menopausal hormone therapy (compared to never use) and new-onset asthma in menopausal women.

The results were based on complete-case analysis of 135 women aged ≥ 45 years in 2008 (excluding 50 women with missing data). Abbreviations: CI, confidence interval.

[a] Age at baseline in 2008.

[b] Adjusted for age, place of residence, level of education, body mass index, tobacco smoking, environmental tobacco smoke, age at menopause, physical exercise, and gynecological conditions, using Frequentist conditional logistic regression.

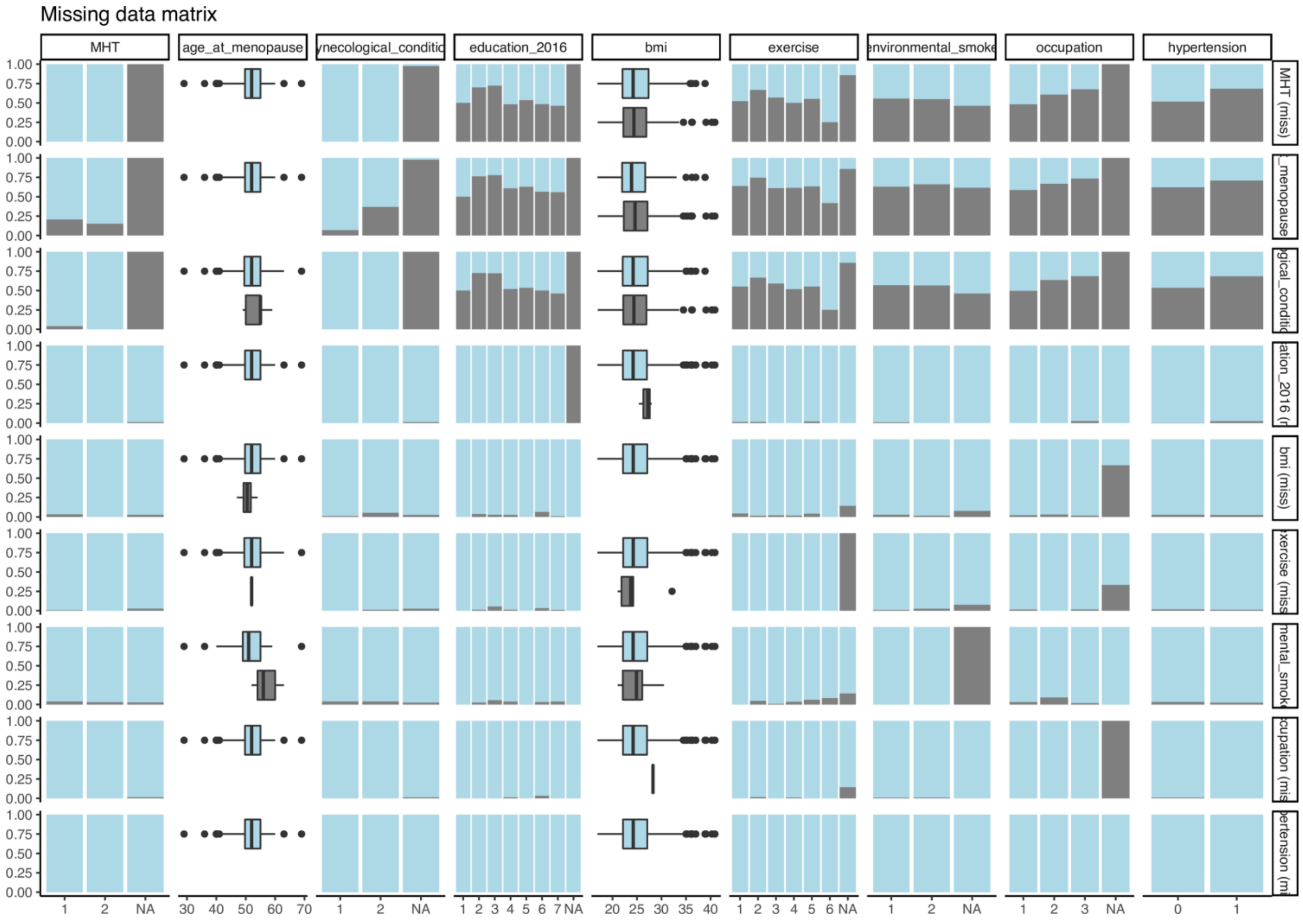

Figure S15. Comparison between women who had data on the incomplete variables and those who did not with respect to auxiliary variables.

Menopausal hormone therapy, the first row; age at menopause, the second row; gynecological conditions, the third row; level of education, the fourth row; body mass index, the fifth row; physical exercise, the sixth row; environmental tobacco smoke, the seventh row. Auxiliary variables include occupation and hypertension.

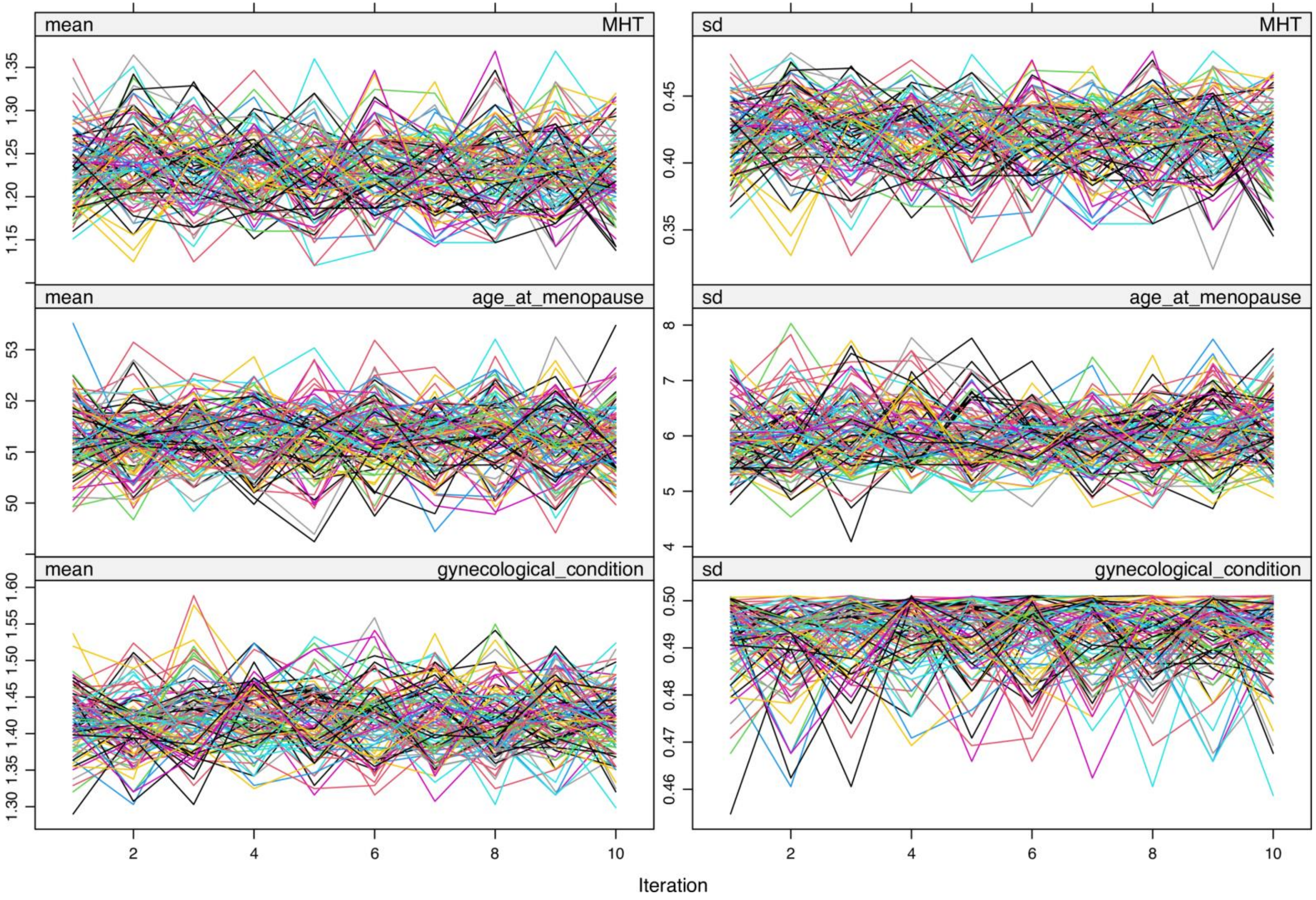

Figure S16. Healthy convergence for the incomplete variables.
Menopausal hormone therapy, the first row; age at menopause, the second row; gynecological conditions, the third row; physical exercise, the fourth row; body mass index, the fifth row; level of education, the sixth row; environmental tobacco smoke, the seventh row.

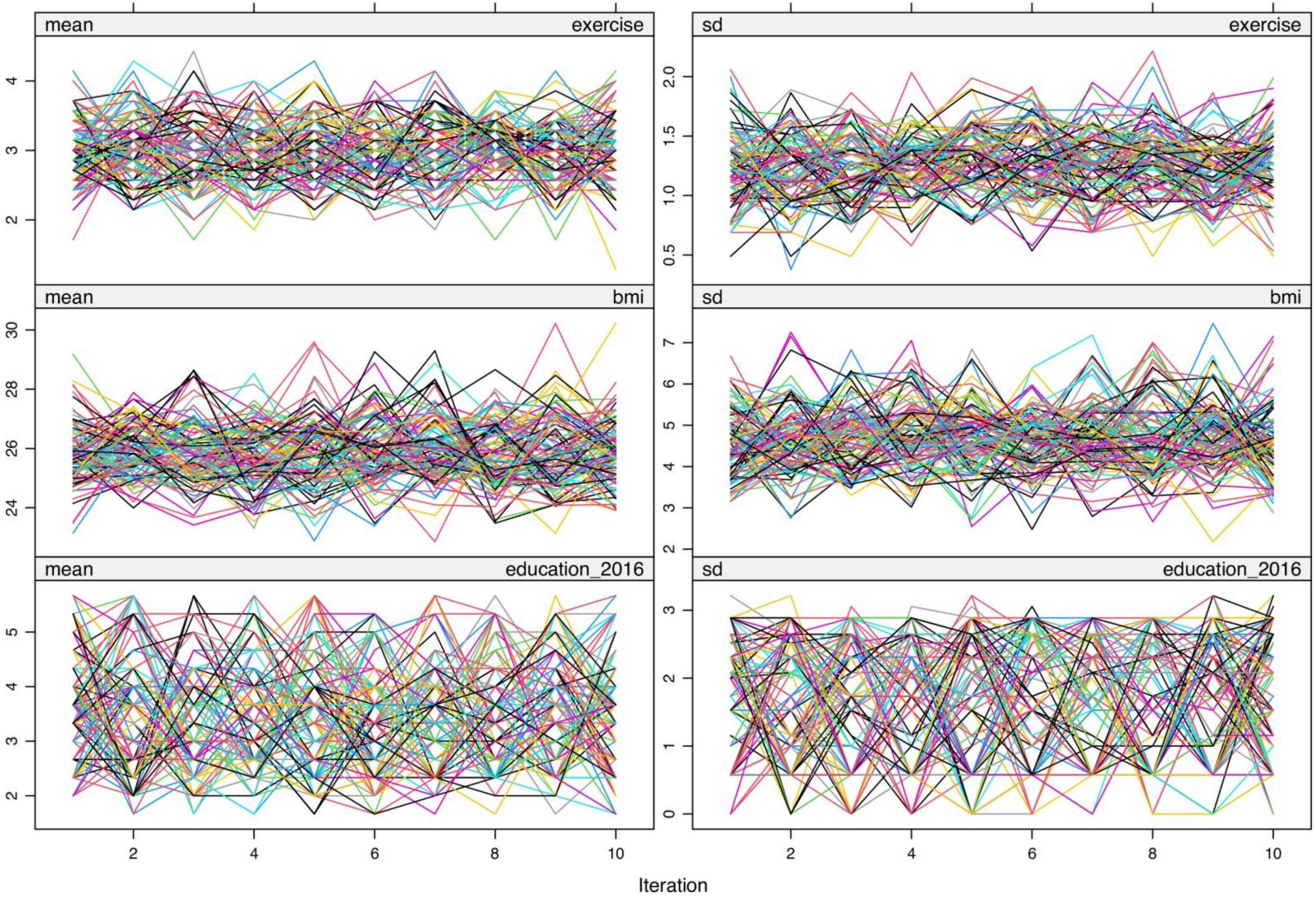

Figure S16. Healthy convergence for the incomplete variables (continued).

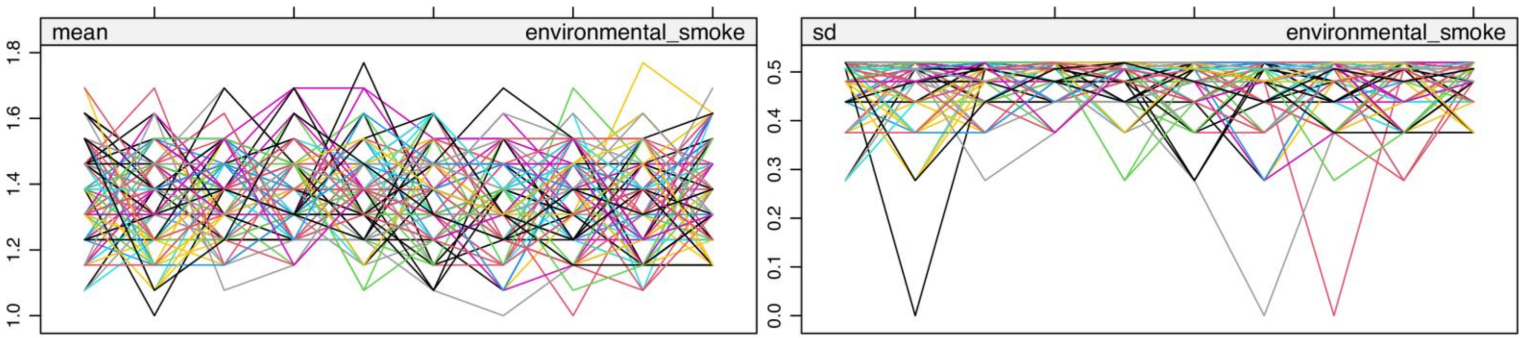

Figure S16. Healthy convergence for the incomplete variables (continued).

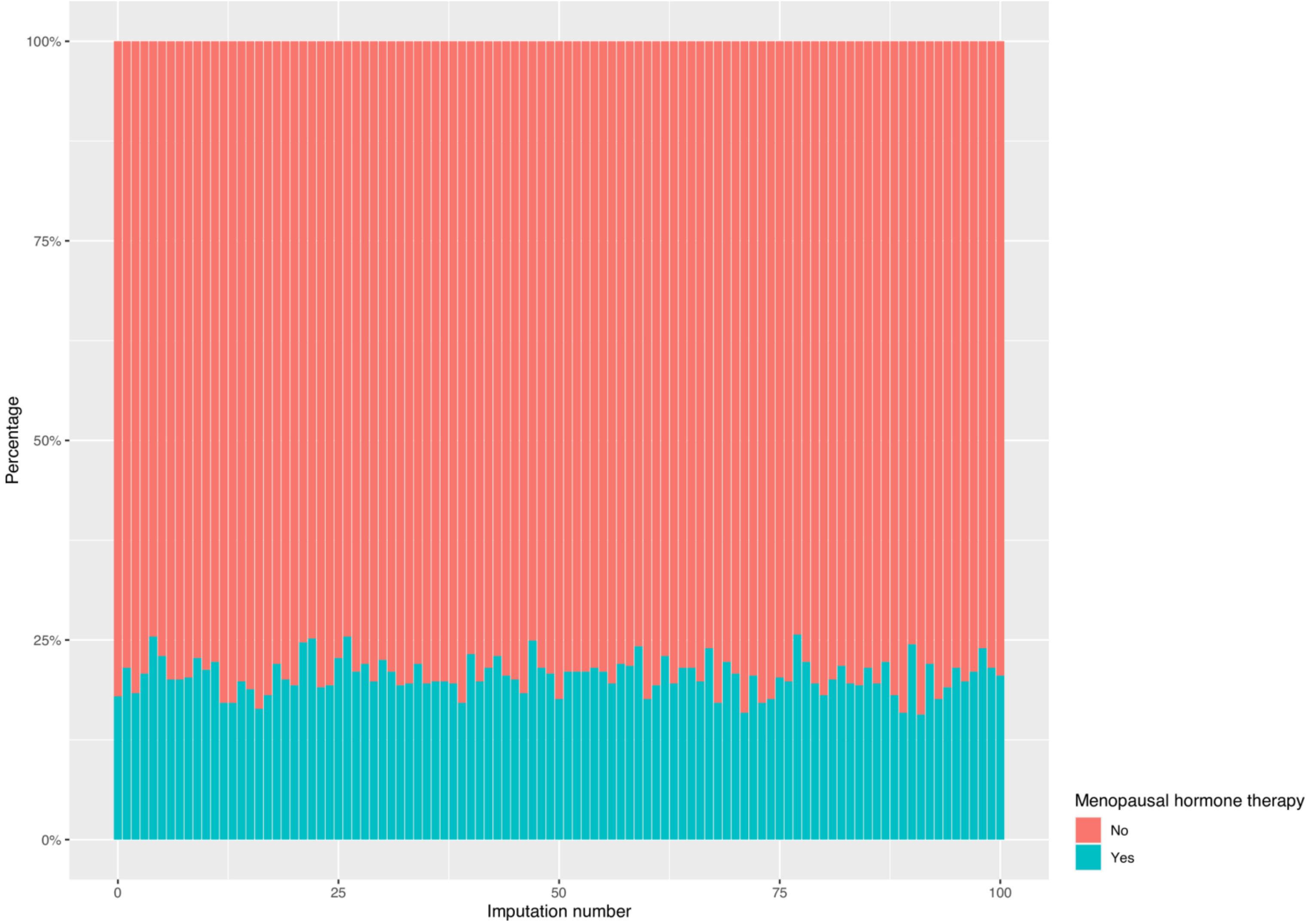

Figure S17. The distribution of use of menopausal hormone therapy in the observed data and in the m = 100 imputed datasets.
Observed data (N = 184), imputation number = 0; imputed data (N = 409), imputation number from 1 to 100.

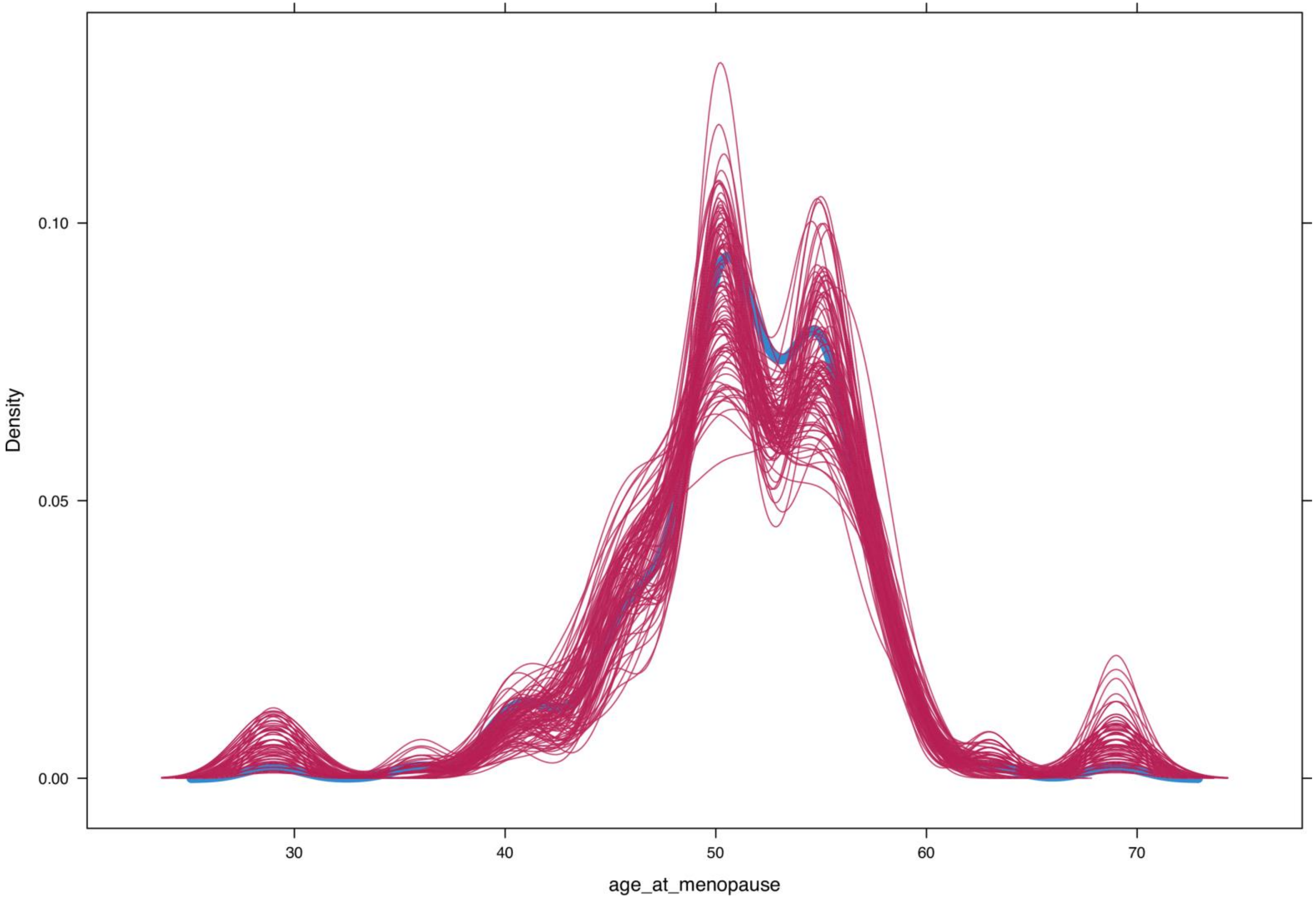

Figure S18. Kernel density estimates for the marginal distributions of the observed data for age at menopause and the m = 100 densities calculated from the imputed data.

Observed data (N = 148), thick blue line; imputed data (N = 409), thin red lines.

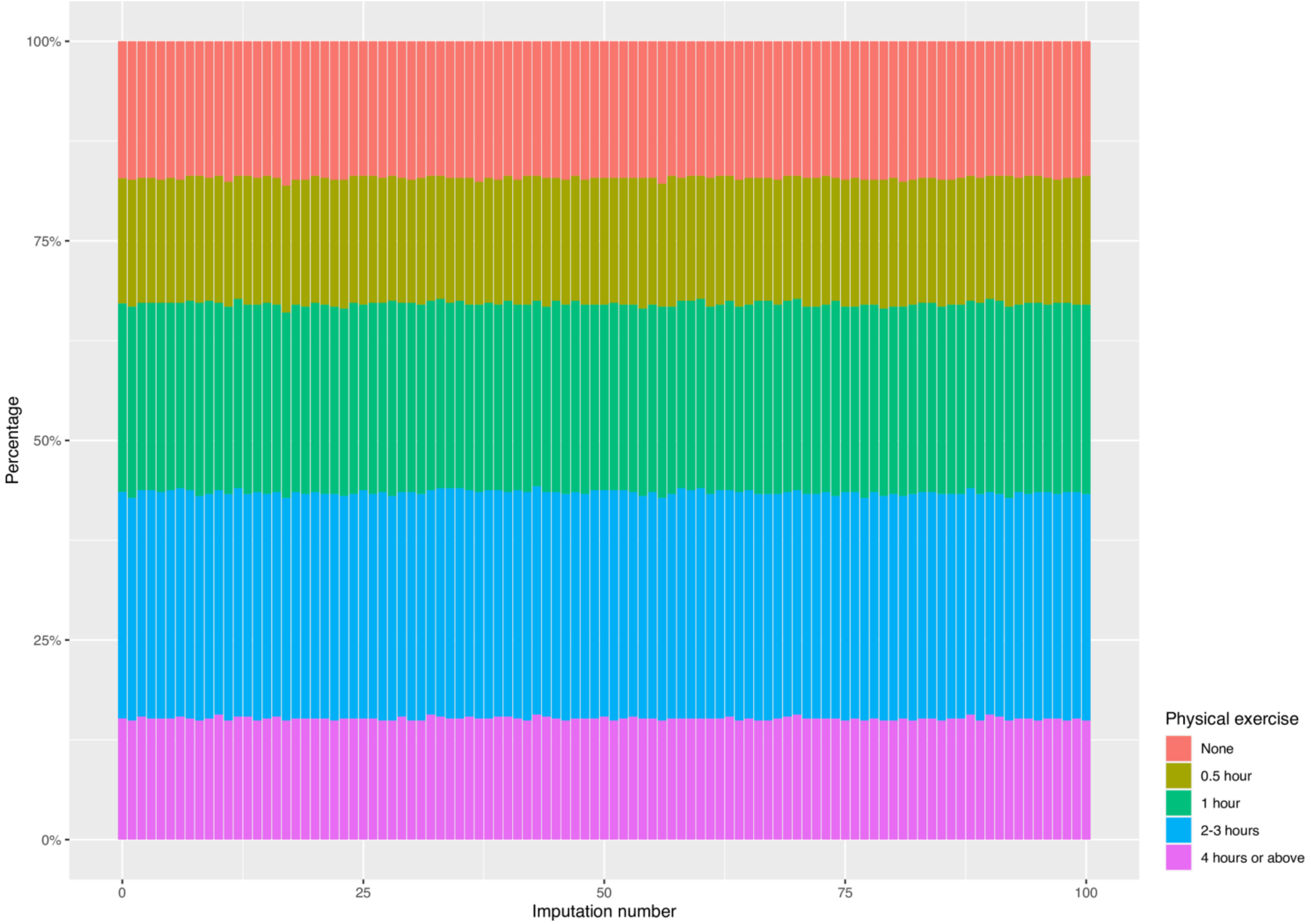

Figure S19. The distribution of physical exercise in the observed data and in the m = 100 imputed datasets.
Observed data (N = 402), imputation number = 0; imputed data (N = 409), imputation number from 1 to 100.

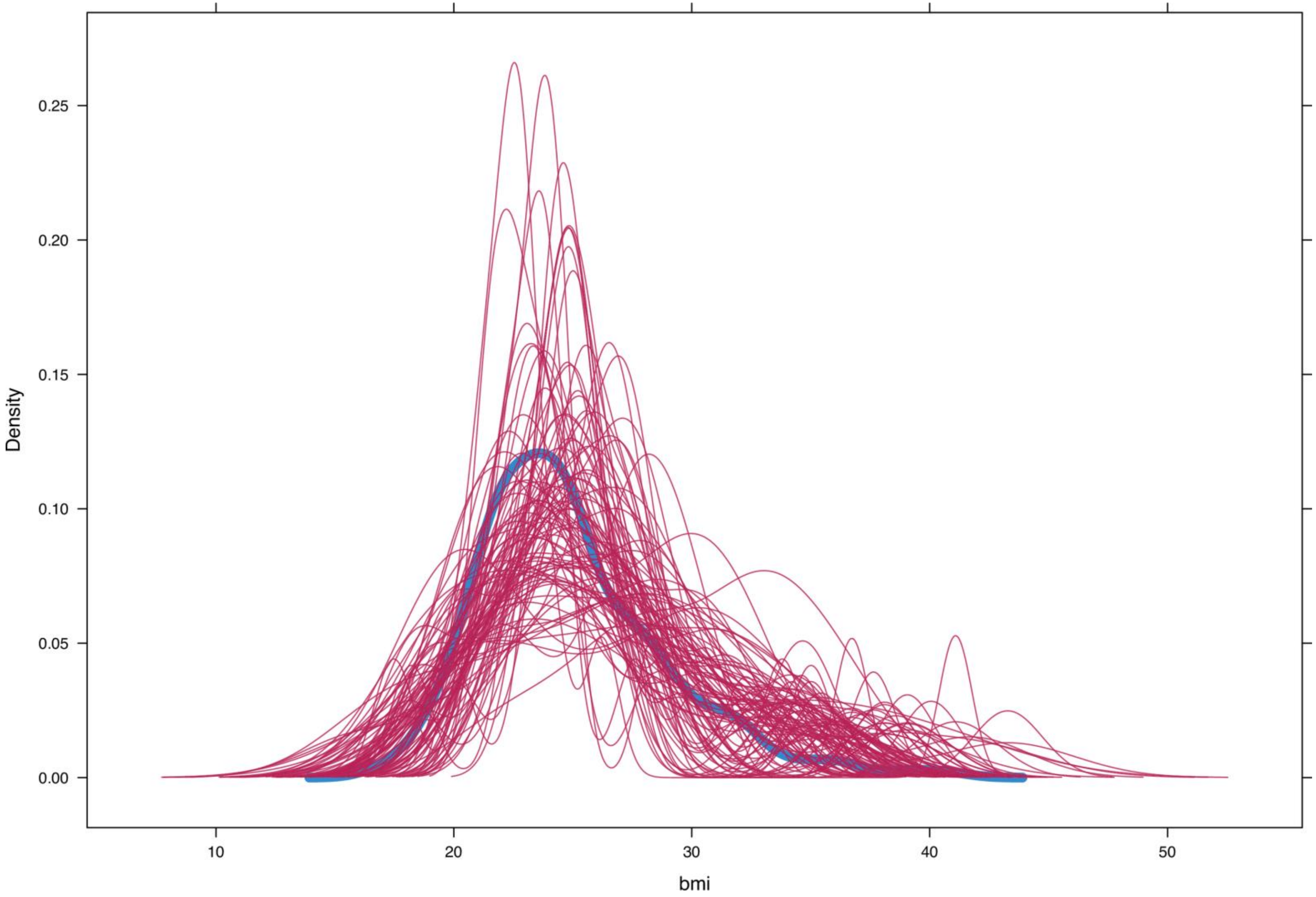

Figure S20. Kernel density estimates for the marginal distributions of the observed data for body mass index and the m = 100 densities calculated from the imputed data.

Observed data (N = 398), thick blue line; imputed data (N = 409), thin red lines.

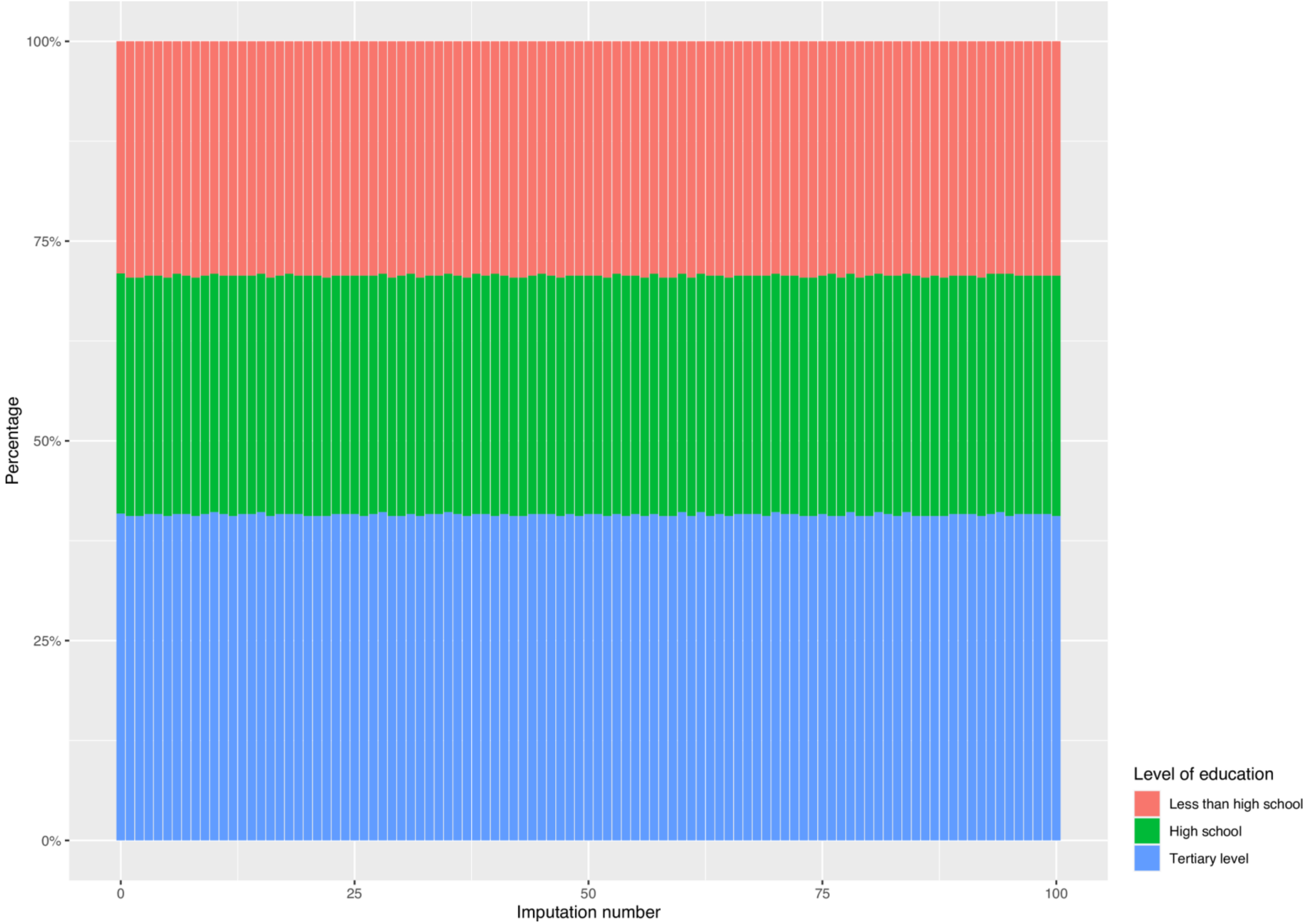

Figure S21. The distribution of level of education in the observed data and in the m = 100 imputed datasets.
Observed data (N = 406), imputation number = 0; imputed data (N = 409), imputation number from 1 to 100.

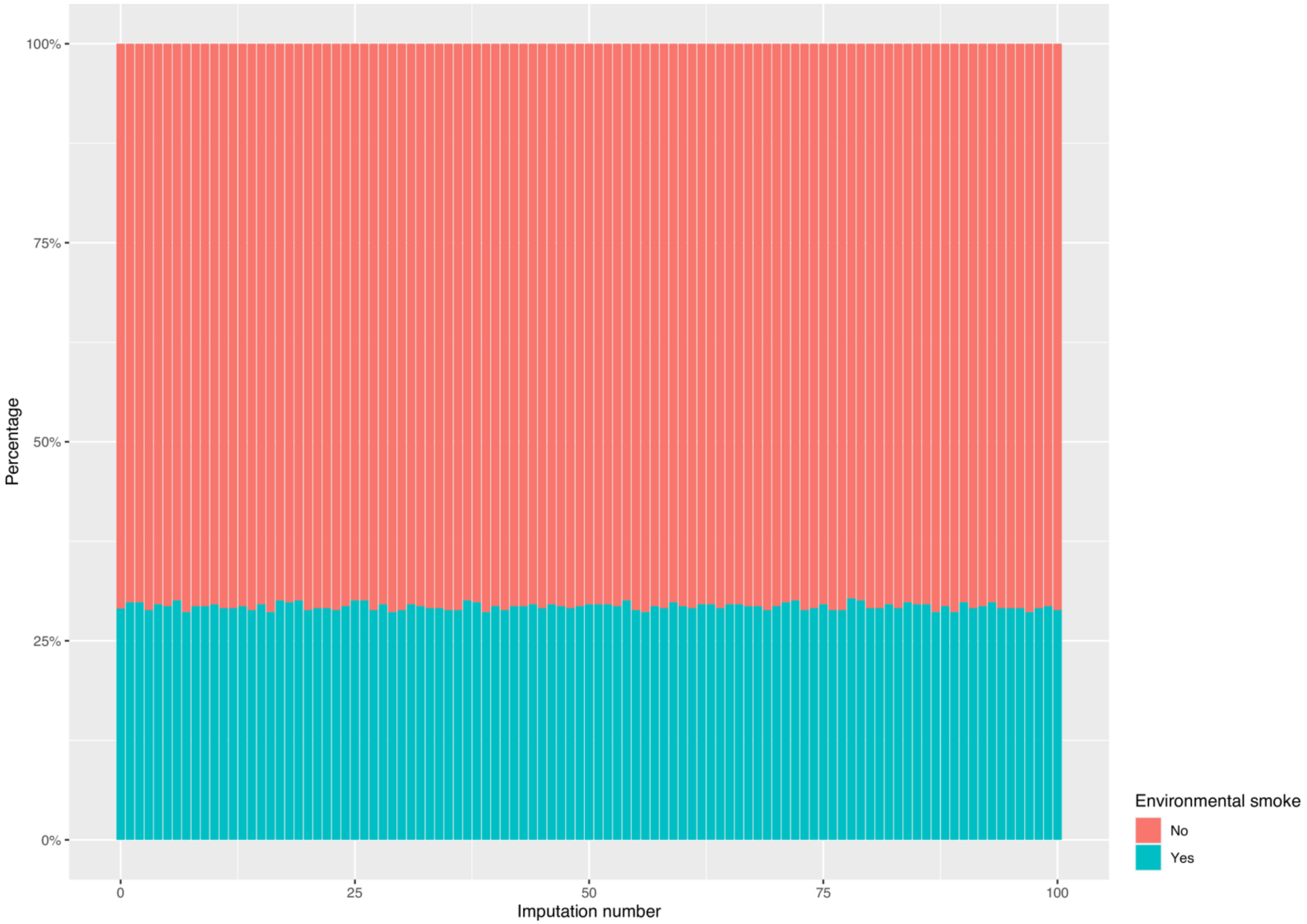

Figure S22. The distribution of environmental smoke in the observed data and in the m = 100 imputed datasets.
Observed data (N = 396), imputation number = 0; imputed data (N = 409), imputation number from 1 to 100.

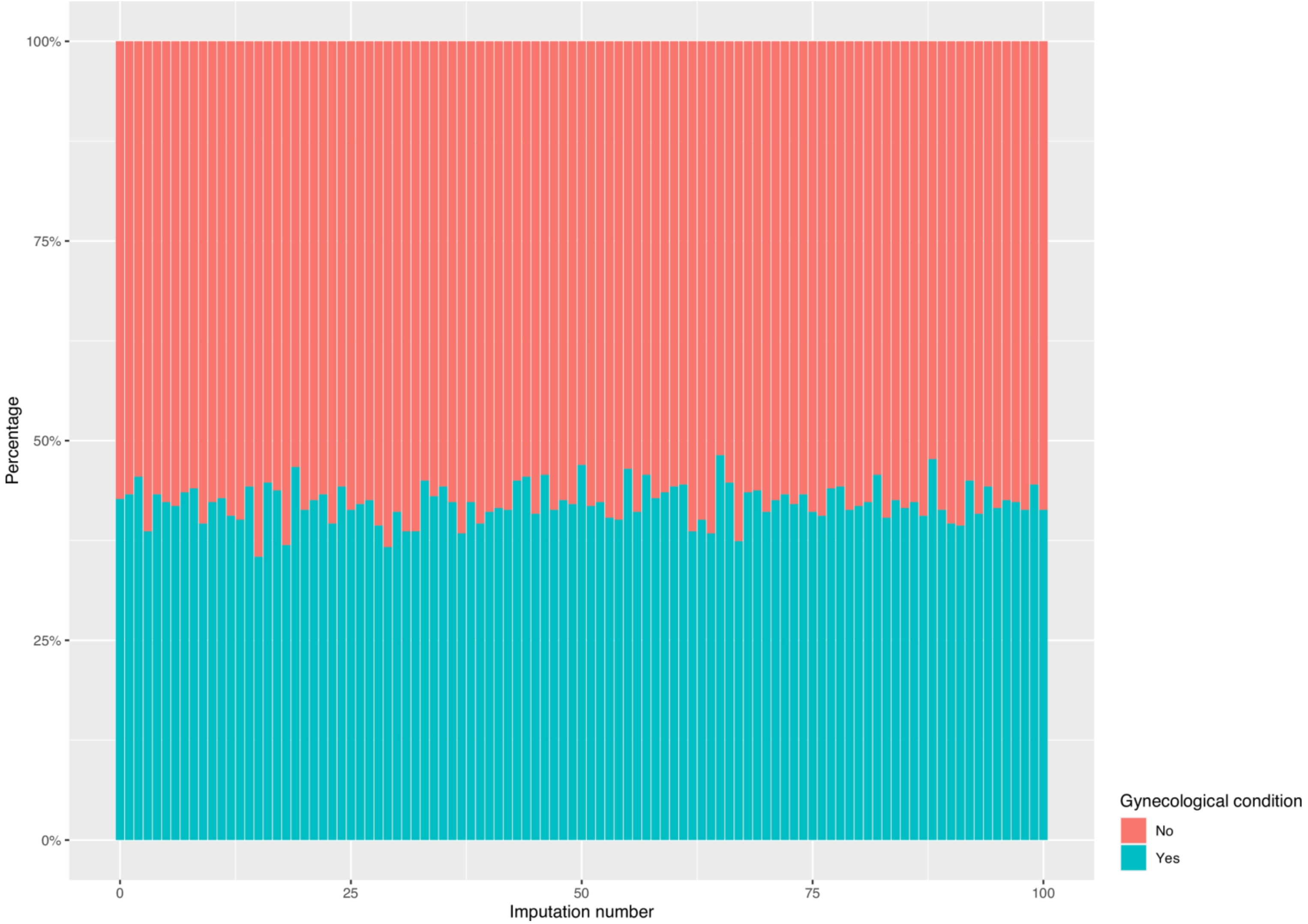

Figure S23. The distribution of gynecological condition in the observed data and in the m = 100 imputed datasets.
Observed data (N = 178), imputation number = 0; imputed data (N = 409), imputation number from 1 to 100.

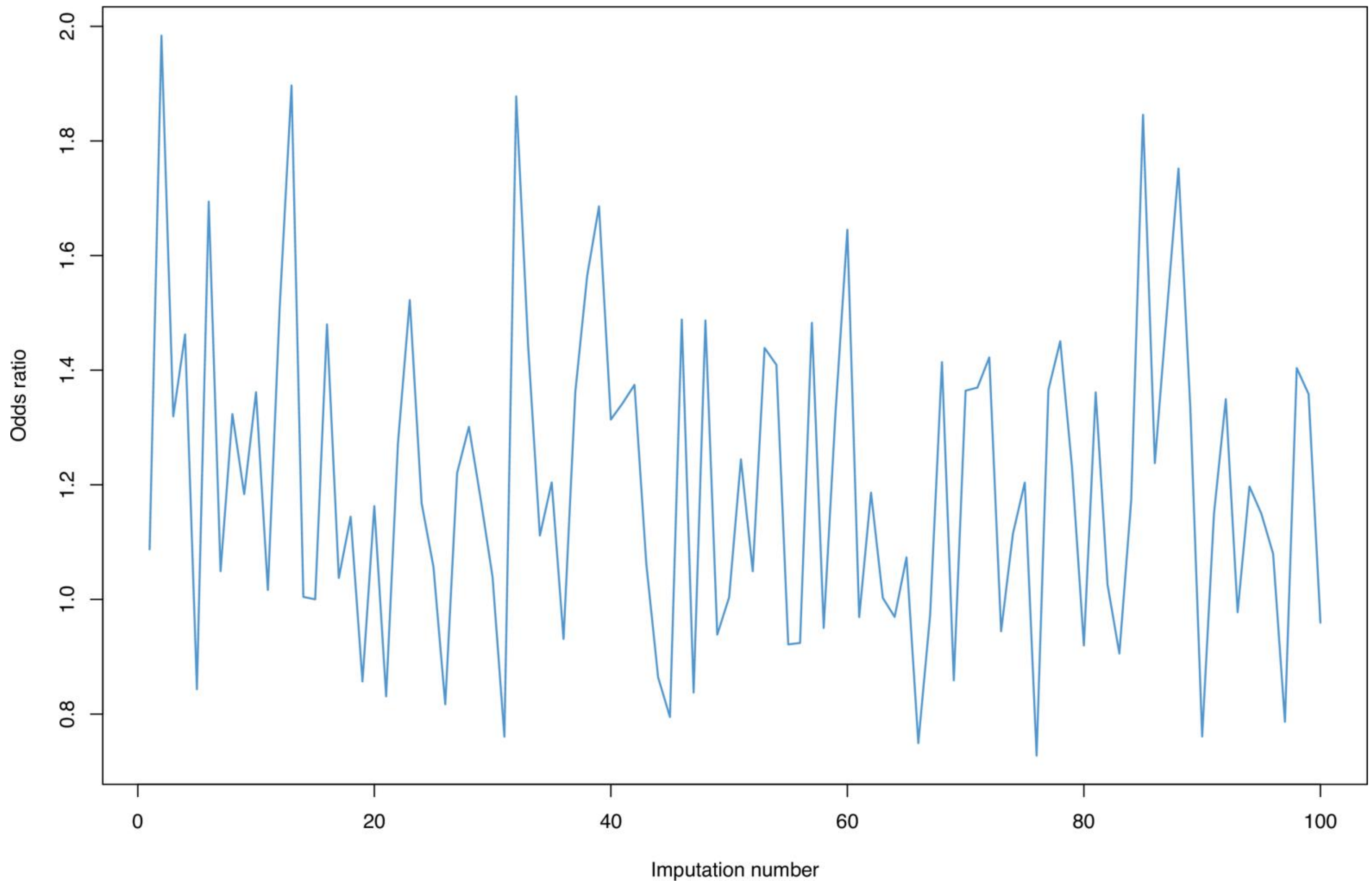

Figure S24. Odds ratios for ever use of menopausal hormone therapy (compared to never use) and new-onset asthma among all menopausal women (N = 409) across the m = 100 imputed datasets.

Adjusted for age, place of residence, level of education, body mass index, tobacco smoking, environmental tobacco smoke, age at menopause, physical exercise, and gynecological conditions, using Frequentist conditional logistic regression.

eReferences

1. Chapter 8. Case-control and cross sectional studies. Accessed June 1, 2022. https://www.bmj.com/about-bmj/resources-readers/publications/epidemiology-uninitiated/8-case-control-and-cross-sectional.
